# Quality assessment of gene repertoire annotations with OMArk

**DOI:** 10.1038/s41587-024-02147-w

**Published:** 2024-02-21

**Authors:** Yannis Nevers, Alex Warwick Vesztrocy, Victor Rossier, Clément-Marie Train, Adrian Altenhoff, Christophe Dessimoz, Natasha M. Glover

**Affiliations:** 1 Department of Computational Biology, University of Lausanne, Lausanne, Switzerland; 2 Swiss Institute of Bioinformatics, Lausanne, Switzerland; 3 Department of Ecology and Evolution, University of Lausanne, Lausanne, Switzerland; 4 Department of Computer Science, ETH Zurich, Zurich, Switzerland

**Keywords:** Quality control, Molecular evolution, Sequence annotation

## Abstract

In the era of biodiversity genomics, it is crucial to ensure that annotations of protein-coding gene repertoires are accurate. State-of-the-art tools to assess genome annotations measure the completeness of a gene repertoire but are blind to other errors, such as gene overprediction or contamination. We introduce OMArk, a software package that relies on fast, alignment-free sequence comparisons between a query proteome and precomputed gene families across the tree of life. OMArk assesses not only the completeness but also the consistency of the gene repertoire as a whole relative to closely related species and reports likely contamination events. Analysis of 1,805 UniProt Eukaryotic Reference Proteomes with OMArk demonstrated strong evidence of contamination in 73 proteomes and identified error propagation in avian gene annotation resulting from the use of a fragmented zebra finch proteome as a reference. This study illustrates the importance of comparing and prioritizing proteomes based on their quality measures.

## Abstract (web summary)

A new tool checks the quality of gene annotations in genome sequences.

## Main

Sequencing many species from diverse taxa will drastically improve comparative genomics methods and our ability to elucidate when and how genes and species evolved^1^, provided the data truly reflect biological reality. This process necessitates rigorous quality control. Robust quality standards for genome assembly have been defined by sequencing initiatives, but improved metrics for genomic features, especially protein-coding genes, are needed^2^. These standards should assess gene repertoire completeness, accuracy of gene models, absence of misannotated non-coding sequences and contamination. A few methods, based on conserved gene markers, can be used to measure the completeness of a gene repertoire (for example BUSCO^3,4^, EukCC^5^, DOGMA^6^ and CheckM^7^) and to some extent contamination from other species (for example EukCC and CheckM). Other quality indicators, such as the UniProt Complete Proteome Detector, flag annotations with an unexpected number of protein-coding genes^8^. However, no existing methods estimate the extent of spurious annotation, which is common in publicly available genomes^9^.

**Fig. 1:**
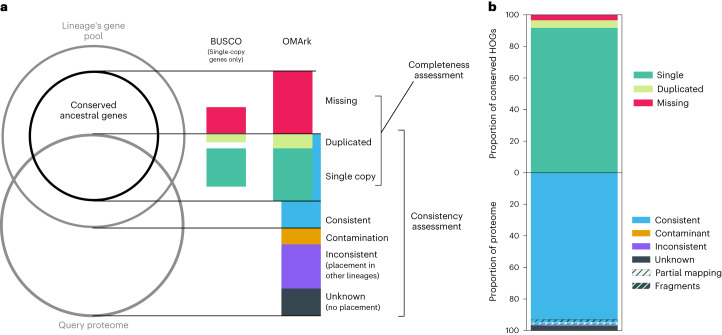
Summary of OMArk proteome quality statistics. **a**, Schematic overview of the OMArk concept and output. OMArk provides two main quality assessment categories: completeness assessment and consistency assessment. Completeness assessment is based on the overlap of the query proteome with a conserved ancestral gene set of the species’ lineage. OMArk classifies genes in the query proteome that are found in a single copy or multiple copies (duplicated) or missing. Completeness assessment is similar to methods like BUSCO but also considers conserved genes that are in multiple copies. Consistency assessment is based on the proportion of query proteins placed in gene families of the correct lineage (consistent), gene families of an incorrect lineage (either randomly (Inconsistent) or to specific species (contamination)) and placed in no gene families at all (unknown). **b**, An example of OMArk’s graphical output for the model organism zebrafish (*Danio rerio*). The top of the stacked bar plot represents the completeness assessment and shows genes that are found in a single copy (dark green; here: 91.78%), duplicated (light green, 4.85%) or missing (red, 3.37%). The lower part of the bar plot represents the consistency assessment and shows taxonomically consistent genes (blue, 96.31%), taxonomically inconsistent genes (violet, 0.65%), contaminants (orange; none in this example) or genes with no detected homology (unknown; black, 3.04%). All categories annotated with hashes correspond to the proportion of partial mappings (black hashes) and fragmented genes (white hashes). Source data

We present OMArk, a method for eukaryotic proteome quality assessment. OMArk rapidly places query protein sequences into known gene families and compares them to the expected families of the species’ lineage. OMArk outputs multiple complementary quality statistics for the query proteome (Fig. 1a). First, it estimates the completeness, based on the proportion of expected conserved ancestral genes present. This is similar to BUSCO but also considers conserved multicopy genes. Second, OMArk estimates the taxonomic consistency (i.e., the proportion of protein sequences placed into known gene families from the same lineage). Sequences placed into gene families from other taxa or not placed at all may be contaminant or erroneous sequences. Thus, OMArk assesses proteome quality by evaluating not only what is expected to be there but also what is not expected to be there—contamination and dubious proteins. This feature, to our knowledge, is not fully provided by any existing methods. We demonstrate OMArk’s accuracy in estimating multiple quality metrics on proteomes with artificially introduced errors and in real-use cases.

## Results

### Software overview

OMArk is available as an open source command-line tool and a web server. The command-line tool is distributed as a python package on Anaconda, PyPI and GitHub (https://github.com/DessimozLab/OMArk). In addition to a query proteome, it needs only a precomputed OMAmer database, which is available for download from the OMA browser^10^.

The web server (https://omark.omabrowser.org) lets users upload a FASTA file of their proteome of interest and visualize or download the results once the computation is done, typically within 35 min for a proteome of 20,000 sequences. Additionally, users can interactively browse and compare precomputed OMArk results for over 8,000 annotation sets from the National Center for Biotechnology Information (NCBI), Ensembl and UniProt.

#### Query protein placement

**Fig. 2:**
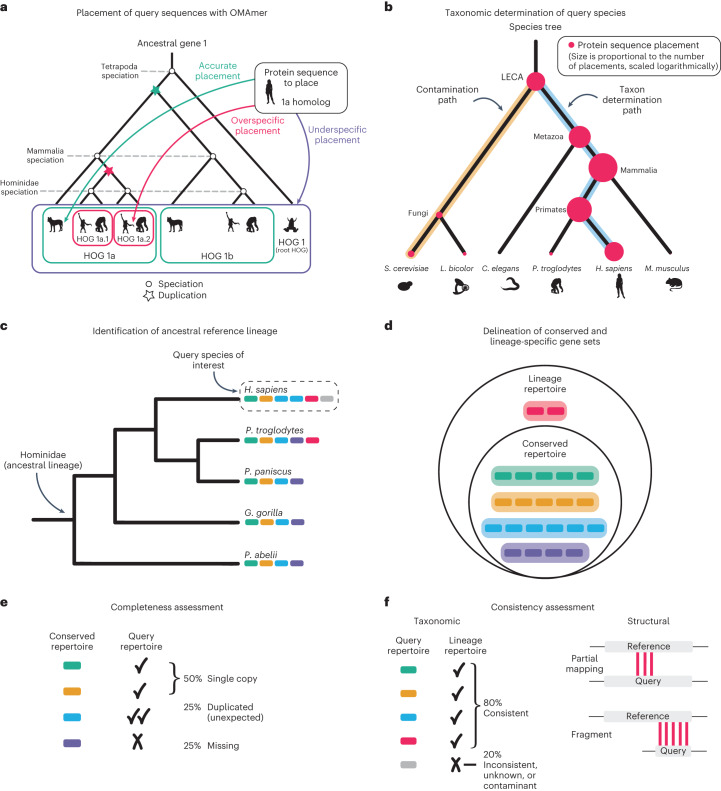
Overview of the OMArk methodology. **a**, Sequences from the query proteome are placed into known HOGs using the *k*-mer-based fast-mapping method OMAmer. Shown is a gene tree with nested gene families (HOGs), delineated by speciation and duplication events. OMAmer provides accurate placement of protein sequences in their correct subfamily. **b**, The specific taxon of the query species is automatically determined by OMArk. Here, the species tree is shown, with protein placements represented by red dots. The size of the dots is logarithmically proportional to the number of placements in a typical scenario but simplified for this schema. The path to the query taxon (blue) is inferred based on the maximal number of placements, and the path(s) to contaminant taxa (gold) are determined as those with more placements than expected by chance. **c**, OMArk defines the ancestral reference lineage for a given query species as the most recent taxonomic level, including the species, and that is represented by at least five species in the OMA database. Here, a species tree is shown with colored bars representing individual genes. **d**, The conserved and lineage-specific gene sets. The conserved repertoire contains all the HOGs defined at the reference ancestral level that cover at least 80% of the species in the clade. These are gene families inferred to be present since the common ancestor. The lineage repertoire is a superset of the conserved repertoire, with the addition of genes that originated later in the lineage and are still present in at least one species in the OMA database. In the repertoires, genes from the different species are grouped into their HOGs. **e**, OMArk assesses completeness by comparing the conserved ancestral repertoire to the query protein sequences and classifying them as single copy, duplicated or missing. **f**, OMArk assesses consistency by comparing the query protein sequences to the lineage repertoire and classifying them as taxonomically consistent, inconsistent, unknown or contaminant. OMArk also assesses gene model structure by classifying query proteins as partial mapping or fragment. Shapes of species shown in **a** and **b** reprinted from Phylopic (www.phylopic.org). Silhouettes of *Homo sapiens* and *Canis familiaris dingo* by T. M. Keesey (public domain), *Pongo abelii* by Gareth Monger (CC-BY 3.0), *Pan troglodytes* by J. Lawley (public domain) and *Xenopus laevis* by Ian Quigley (CC-BY 3.0). Silhouettes of *Saccharomyces cerevisiae* by W. Decature (public domain), *Laccaria* by R. Percudani (public domain), *Caenorhabditis elegans* by J. Warner (public domain) and *Mus musculus* by S. Miranda-Rottman (CC-BY 3.0). Source data

OMArk takes as input a proteome FASTA file in which each gene is represented by at least one protein sequence. OMArk starts with OMAmer^11^, a fast *k*-mer-based method that assigns proteins to gene families and subfamilies (Fig. 2a), represented as hierarchical orthologous groups (HOGs)^12^. These gene families are predefined in the OMA database^13^ using over 2,500 species but could in principle be used with other databases using the HOG concept.

#### Species identification

To infer the species composition of the query proteome, OMArk tracks the protein placement into gene families and their taxa of origin (Fig. 2b). Ideally, a species’ proteome will have placements only into gene families from its ancestral lineage. For example, human genes will have originated at the common ancestor of primates, mammals and vertebrates, but not rodents. OMArk starts from this assumption and identifies paths in the species tree where placements are overrepresented, and it then selects the most recently emerged clade as the inferred taxon. If multiple paths are overrepresented, OMArk reports the most populated as the main taxon and all others as contaminants.

#### Ancestral reference lineage identification

Based on the main taxon placement, or a user-specified taxonomic identifier, OMArk selects an ancestral lineage: the most recent taxon that contains the species of interest and at least five species in the OMA database (Fig. 2c). The selected ancestral lineage is provided in OMArk’s output.

#### Completeness assessment

OMArk selects all gene families that were present in the common ancestor of the ancestral lineage and still are present in at least 80% of its extant species (conserved repertoire; Fig. 2d). The presence of these gene families serves as a proxy for its proteome completeness. OMArk reports the number of selected gene families, their identifiers and the proportion of the conserved gene families that are found in the query proteome as a single copy or duplicated (multiple copies) or are missing (Fig. 2e). An incomplete proteome would have a high proportion of missing gene families.

Contrary to BUSCO, the conserved genes are not necessarily expected to exist in single copies in extant genomes, although they were likely a single gene in the lineage’s ancestor. Thus, duplicated genes are classified as ‘expected’ if they correspond to a known duplication that occurred after the ancestral lineage’s speciation or ‘unexpected’ otherwise. If the ancestral lineage has a lower ploidy level than the query species due to subsequent whole-genome duplication (WGD; for example, ancestral diploid compared to a tetraploid), then the query proteome will appear as massively duplicated. Users should interpret the results in the context of their query species’ ploidy.

#### Consistency assessment

The main advantage of OMArk is that it evaluates the consistency of all the genes in the query proteome compared to what is known for its lineage, both taxonomically and structurally.

Taxonomic consistency classifies query proteins based on their taxonomic origin by comparing them to the lineage’s known gene families (lineage repertoire; Fig. 2d). Proteins fitting this lineage repertoire are classified as consistent, whereas those that fit outside are classified as either inconsistent or contaminant (Fig. 2f). The contaminant category contains all inconsistent placements that are closer to a contaminant species than to the main species, as determined by the species identification step. Proteins with no gene family assignment are classified as unknown.

Structural consistency classifies query proteins based on sequence feature comparisons with their assigned gene family. Proteins only sharing *k*-mers with their gene families over part of their sequence are labeled partial mappings, whereas proteins with lengths less than half their gene family’s median length are labeled fragments (Fig. 2f).

Taxonomic and structural consistency are complementary parts of the consistency assessment performed over the whole proteome and help identify annotation errors, a feature lacking in most quality assessment methods. A proteome with a high proportion of consistent proteins indicates more reliable annotation. Conversely, a high proportion of partial mappings and fragments indicates potential gene model inaccuracies. Inconsistent proteins suggest either gene families not previously identified in the target clade or, if they are primarily partial or fragments, sequences with biased composition. Similarly, unknown proteins may be sequences without close homologs or annotation errors. Thus, not all proteins classified as inconsistent or unknown are necessarily errors, but an unusually high proportion may indicate a systematic error in the annotation.

An example of the OMArk output for the *Danio rerio* proteome shows it has a high completeness (96.6%) and consistency (96.3%), as expected for a well-curated model species (Fig. 1b).

### Validation on simulated proteomes

To evaluate OMArk’s ability to provide accurate quality assessment, we simulated cases of genome incompleteness, erroneous sequences, gene fragmentation or fusion, and cross-species contamination. We used two datasets of eukaryotic proteomes (Supplementary Table 1): a dataset comprising nine model species known for their high quality (model dataset) and a dataset including 16 species representing eukaryotic diversity and absent from the reference OMA database (representative dataset).

#### Simulated incompleteness

**Fig. 3:**
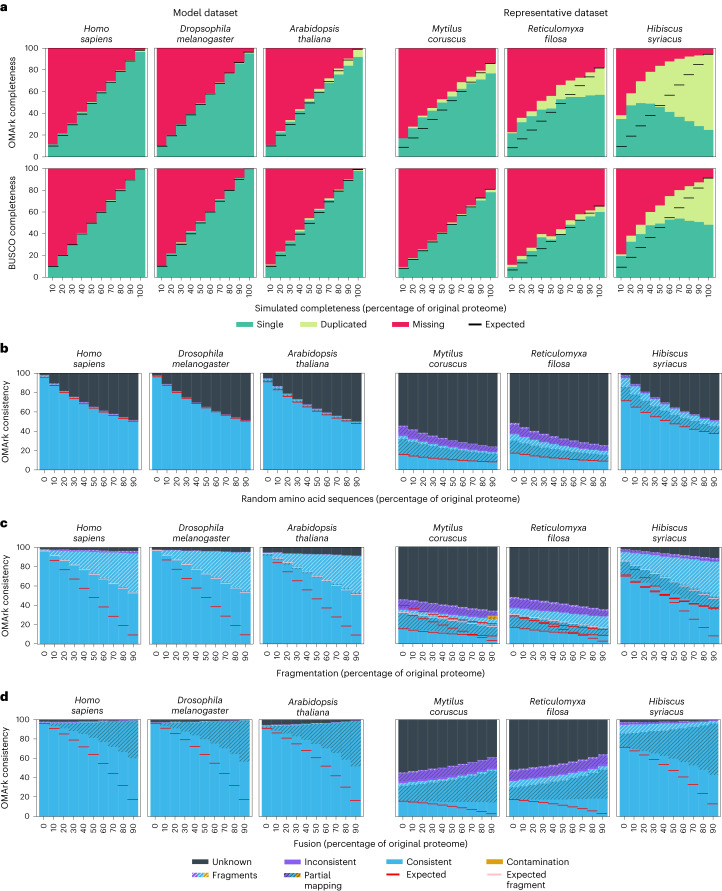
OMArk results for simulated proteomes. **a**–**d**, Three example species of the model dataset (left) and the representative dataset (right) are shown for each simulation. Each simulated error in panels a–d was applied to 10%–90% of the proteome (*x* axis). **a**, Simulated incompleteness. OMArk (top) and BUSCO (bottom) results for the datasets. Colors represent the part of the conserved gene set found in a single copy (green) or duplicated (light green) or are missing (red). The simulated completeness corresponds to the percentage of the genome that has been randomly selected in each simulation. Horizontal black lines show the expected completeness (that is, the measured completeness for the source proteome). **b**, Erroneous sequence simulation. Colors represent proteins which map to the correct lineage (consistent, blue), to another lineage (Inconsistent, violet) or have no homologs (unknown, black). Hashes indicate structural inconsistency relative to the gene family (either partial mapping (black hashes) or fragmented genes (white hashes)). The appended error (*x* axis) corresponds to the quantity of erroneous sequences that was added to the proteome as a percentage of its original protein number. Horizontal red lines indicate the expected number of structural and taxonomically consistent genes, considering the proportion in the source proteome and the known introduced error. **c**, Fragmented sequence simulation. The *x* axis corresponds to the percent of the proteome that has been fragmented. The pool of artificially fragmented genes are cut randomly to be between 10% and 90% of the original length of the protein. Horizontal red lines indicate the expected number of nonfragmented taxonomically consistent genes, considering the proportion in the source proteome and the known fragment rates; horizontal pink lines indicate this proportion if half of the fragments are detected. **d**, Fused sequence simulation. The *x* axis corresponds to the percent of the proteome that has been fused. Pairs of proteins are selected randomly and appended together to simulate fusion. The fused protein gets added to the proteome while the original proteins get removed. Horizontal red lines indicate the expected number of structural and taxonomically consistent genes, considering the proportion in the source proteome that have been fused. Ancestral lineages for the six shown species are *Homo sapiens*, Hominidae; *Drosophila melanogaster*, melanogaster subdivision; *Arabidopsis thaliana*, Brassicaceae; *Mytilus coruscus*, Lophotrochozoa; *Reticulomyxa filosa*, SAR (Stramenopiles-Alveolata-Rhizaria) supergroup; and *Hibiscus syriacus*, Malvaceae. Source data

For each proteome in the datasets, we simulated incompleteness by removing varying percentages (10%–90%) of random proteins. OMArk’s results closely approximate the simulated completeness in most cases, although it tends to overestimate it (Fig. 3a and Supplementary Figs. 1 and 2). The error margin is lower in the model dataset (+2.3% on average) than in the representative dataset (+9.9% on average). For both datasets, OMArk’s performance is similar to BUSCO’s, but BUSCO overestimates completeness by a slightly smaller margin (+2.1% and +6.1% on average for the model and representative datasets, respectively; Supplementary Figs. 1 and 2).

Both methods overestimate completeness in species with a high number of duplicated genes. This effect is expected, as reporting them as missing requires all copies to be absent. This trend is more pronounced in OMArk, because OMArk does not require conserved genes to be in a single copy in extant species, resulting in a more inclusive set of conserved gene families. Thus, because OMArk reports more duplicates, it overestimates completeness more than BUSCO. This trend is observed in both datasets but especially the representative dataset, as these proteomes have a higher average proportion of duplicated genes (8.4% for the representative dataset versus 2% for the model dataset).

This high level of detected duplication in the representative dataset can be explained by the selected ancestral lineages, which are more distantly related than those selected in the model dataset. Thus, ancestral gene families in the representative dataset may have had more time to undergo duplication. Furthermore, WGD events that occurred after the ancestral lineage can lead to high levels of reported duplication in OMArk and BUSCO. A striking example is the *Hibiscus syriacus* proteome, where OMArk reports nearly 70% of the genes as duplicates. These results are due to *H. syriacus* being a tetraploid, having undergone two WGD events after the last Malvaceae common ancestor^14^. Because the Malvaceae clade was selected as the ancestral lineage by OMArk, the higher number of duplicates corresponds to the genes that were retained as two copies or more after the WGD.

#### Simulated erroneous sequences

We simulated erroneous sequences by adding randomly generated sequences, from 10% to 90% of the proteome, to each proteome in the model and representative datasets. As a result, there was a corresponding increase in the proportion of Unknown proteins, given that these added sequences lacked detectable homologs (Fig. 3b). In all simulations, OMArk detected the expected proportion of taxonomically and structurally consistent genes, indicating that this category accurately represents the proportion of high-confidence coding sequences. Results were similar whether the sequences were generated from random nucleotides or designed to resemble the target species’ proteins (Supplementary Results: Simulation Results and Supplementary Figs. 3–6).

#### Simulated fragmentation

We simulated fragmented proteomes by randomly selecting sequences and then randomly removing between 10% and 90% of their length, ranging from 10% to 90% of the proteome. OMArk identified an increasing proportion of fragmented, taxonomically consistent proteins, reaching up to half the known number of fragmented sequences. This result is expected, as OMArk only identifies fragments that are less than half the gene family’s median protein length and thus will not detect fragments that are 51% to 90% of the original protein size. Given the modified expected fragmentation detection rate (only half the simulated fragments), there is only a slight underestimation of consistent, nonfragmented proteins: 0.6% for the model dataset and 1.8% for the representative dataset (Fig. 3c and Supplementary Figs. 7 and 8). We also detected a slight increase in unknown proteins, possibly because these fragments are too short to be detected as homologs of existing genes.

#### Simulated fusion

We simulated cases of fused protein-coding genes by merging pairs of randomly selected proteins, ranging from 10% to 90% of the proteome, and added them to the proteomes while removing the original proteins. We expected that OMArk would associate these fused proteins to one of the existing HOGs but as a partial match, as only part of the sequence would be in common with the HOG. However, the increase in partial mappings as the proportion of fused genes rises was less than expected. The proportion of structurally and taxonomically consistent genes was on average 17.6% higher than expected for the model dataset and 13% higher than expected for the representative dataset (Fig. 3d and Supplementary Figs. 9 and 10).

#### Simulated contamination

We simulated contamination by introducing sequences from bacteria, fungi, microbial eukaryotes or humans to the model and representative datasets. OMArk accurately identified the taxonomic origin of the contaminant, though its sensitivity varied, especially with a low number of contaminant proteins. For bacterial and fungal sources, contamination became detectable with as few as ten contaminant proteins, corresponding to ~10 kbp contaminant bacterial DNA or ~25 kbp fungal DNA. Contamination was reliably detected at 50 or more contaminant proteins (~50 kbp bacterial DNA, ~125 kbp fungal DNA). However, for other eukaryotic species, precise contamination detection required at least 100 to 200 contaminant proteins (~200–700 kbp free-living unicellular eukaryote DNA). OMArk missed contamination when the contaminant had no close relative in OMA or was too closely related to the contaminated species (Supplementary Table 2; Supplementary Results: Contamination simulation). Specifically, OMArk only detected human sequence contamination in vertebrates at high levels (1,000 proteins; ~150 Mpb human DNA) and not at all in mammals.

### OMArk results for 1,805 eukaryotic reference proteomes

**Fig. 4:**
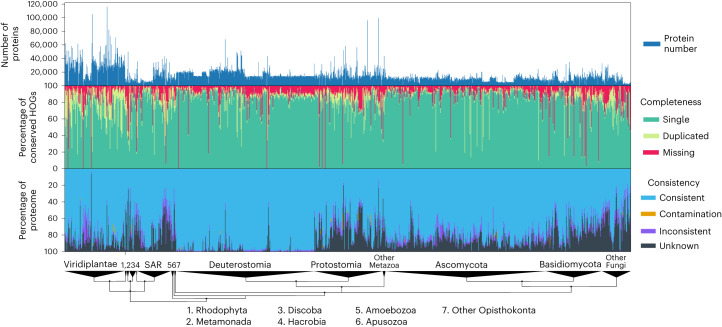
OMArk results on 1,805 eukaryotic UniProt reference proteomes. Bar graphs of the number of canonical proteins in each proteome (top). Completeness assessment showing the proportion of conserved genes (green) in the proteome, with a breakdown among single-copy (light green), duplicated (dark green) and missing (red) genes in all proteomes (middle). Consistency assessment showing the proportion of accurately mapped proteins (consistent; blue), incorrectly placed proteins (Inconsistent; purple), contaminant proteins (orange) and proteins with no mapping (unknown; black) (bottom). Genomes are ranked by taxonomy, with major eukaryotic taxa shown on a taxonomic tree at the bottom. Source data

Comparing protein-coding gene annotations between closely related species, including one ‘gold standard,’ is essential to assess annotation quality^2^. Thus, we ran OMArk on a set of 1,805 Eukaryotic UniProt proteomes to serve as a reference dataset (Fig. 4 and Supplementary Table 3). We provide quality assessments for major clades and detailed analyses of specific proteomes with low-quality results in Supplementary Results: Results on UniProt Reference Proteomes. All results can be visualized on the OMArk web server (https://omark.omabrowser.org) and compared to those of closely related species.

### OMArk and BUSCO comparison

**Fig. 5:**
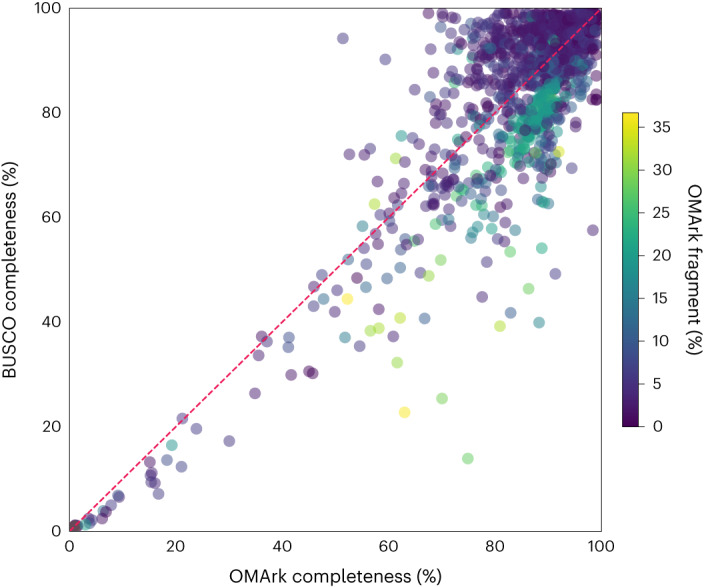
Comparison of mapped proteins between OMArk and BUSCO. Each point on the scatterplot is one of the 1,805 Eukaryotic UniProt Reference Proteomes assessed by both methods. The *x* axis is the percentage of the conserved set of ancestral genes found in the query proteome by OMArk. The *y* axis is the percentage of BUSCO genes found in the query proteome by BUSCO. Both completeness scores include duplicated and fragmented proteins. Proteomes are colored by the percentage of fragments found in the proteome, as determined by OMArk. Source data

We compared OMArk and BUSCO for assessing completeness for the 1,805 Eukaryotic UniProt Reference Proteomes. We define completeness as the total percentage of conserved genes from either BUSCO or OMArk that are classified as single copy, duplicated copies or fragments in the query proteome (that is, not missing). Note that this differs from BUSCO’s definition of completeness, which does not include fragments. OMArk and BUSCO yield similar results overall, with a Pearson correlation of 0.86 for completeness across the 1,805 proteomes (Fig. 5). Disparities are expected, as OMArk considers both single-copy and multicopy genes, whereas BUSCO is restricted to single-copy genes. For 57% of the proteomes, BUSCO versus OMArk completeness differed by 5% or less. Where the difference was larger, proteomes considered more complete by OMArk typically exhibited more fragments, indicating OMArk’s ability to identify fragmented proteins without categorizing them as missing.

The proteome’s lineage also influenced the disparity in completeness scores between BUSCO and OMArk. Certain BUSCO lineages, such as Liliopsida and Stramenopiles, were often deemed as more complete by BUSCO, whereas lineages such as Aves and Nematoda tended to be deemed as more complete by OMArk (Supplementary Fig. 14). This bias may stem from the number of ancestral genes assessed, as fewer BUSCO genes or conserved HOGs generally resulted in higher BUSCO completeness. Conversely, a higher number of BUSCO genes or conserved HOGs resulted in higher OMArk completeness. Additionally, when OMArk deemed a proteome as more complete, the OMA database typically had fewer species in the relevant clade than for proteomes where BUSCO estimated a higher completeness (Supplementary Table 7). Thus, the lineage and consequently the number of conserved genes used for assessment affects completeness in both BUSCO and OMArk. A larger set of conserved genes and more species in the lineage of interest likely lead to more accurate completeness assessments.

Runtime comparison over the same set of proteomes showed OMArk is generally faster in terms of total CPU time, with an average of 9.2 min per proteome for OMArk versus 25.2 min per proteome for BUSCO for all 1,805 proteomes. BUSCO’s runtime largely depends on the number of BUSCO genes used in the assessment, whereas OMArk’s runtime depends mainly on the number of proteins in the query proteome.

These results highlight the biases inherent in each tool. Ultimately, we advise to use both software packages to obtain the most informative gene repertoire quality assessment. More comparisons are detailed in the Supplementary Results: Comparison with BUSCO on UniProt Reference Proteomes.

### Contamination in public databases

OMArk detected 124 contamination events across 79 of 1,805 proteomes, some with multiple contaminating species (list in Supplementary Table 4). Two of them, *Ricinus communis* and *Lupinus albus*, were found to be contaminated by ten and seven species, respectively (mostly bacteria and one fungus), indicating that extreme cases of contamination persist in public databases. We independently verified each contamination case using BLAST and BlobToolKit Viewer (Supplementary Table 4) and confirmed 117 (93.6%) of the contamination events in 73 species.

### Error propagation in some avian proteomes

We detected widespread presence of fragmented genes in the 234 avian species from the UniProt Reference Proteomes (median proportion of taxonomically consistent fragments: 18.3%, standard deviation: 4.8%). However, this was not observed in well-studied birds such as chicken (*Gallus gallus*; proportion of taxonomically consistent fragments: 2.4%; Supplementary Fig. 18). The proportions of fragments depended mainly on the source of the proteome. Most of the highly fragmented proteomes originated from the same source, the Bird 10 K consortium annotation pipeline^15^, and tended to have fragments in the same gene families, suggesting systematic bias (Supplementary Figs. 19 and 20; Supplementary Results: Analysis of avian proteomes). Annotations for these genomes were performed using, among other sources of evidence, homology from the Ensembl 85 (ref. ^16^) annotation of zebra finch^15^ (*Taeniopygia guttata*; taeGut3.2.4 assembly). OMArk also detected a high proportion of fragments in this older version of the zebra finch proteome (proportion of taxonomically consistent fragments: 20.3%), but not in the latest version (0.5% of taxonomically consistent fragments; Ensembl 99 + ; bTaeGut1_v1.p assembly). Furthermore, a high proportion of genes fragmented in the Bird 10 K proteomes were also fragments in the older zebra finch proteome (Supplementary Fig. 21). These results suggest fragments in these bird proteomes likely result from propagation from the fragmented taeGut3.2.4 proteome.

### Selection of high-quality proteomes among close species

**Fig. 6:**
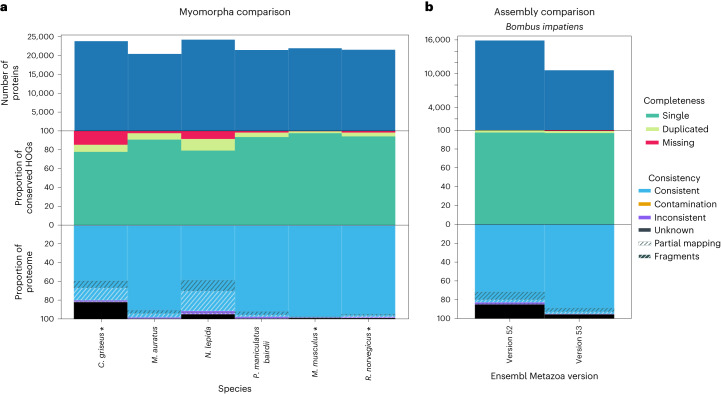
OMArk comparisons between closely related species within a clade or assembly versions. **a**, OMArk results for different Myomorpha proteomes. The species currently in OMA which contributed to the ancestral reference lineage gene families are shown with an asterisk. Mouse (*Mus musculus*) and the Norwegian rat (*Rattus norvegicus*) stand out as high-quality annotations. **b**, OMArk results for *Bombus impatiens*, comparing two different versions of the assembly. The newest assembly (version 53) shows an improvement in gene set consistency, with a slight decrease in completeness, although the number of proteins substantialy decreased. Source data

OMArk’s quality assessment depends on the selected ancestral lineage. Thus, a best practice is to compare the results to species sharing the same ancestral lineage. We illustrate this by comparing the OMArk results of a model species, *Mus musculus*, with its close relatives within the Myomorpha clade, a group of mouse-like rodents (Fig. 6a). As expected, the well-curated species *Mus musculus* and *Rattus norvegicus* scored best, both in completeness and consistency. Several other species in the clade exhibited noticeable quality issues, despite being in the OMA database and contributing to the ancestral reference HOGs (for example, *Cricetulus griseus*). We observed similar patterns for other model organisms consistently ranking as the best proteomes in their clade (detailed in Supplementary Results: Comparison of proteomes from closely related species; Supplementary Figs. 22–30).

These results demonstrate OMArk’s ability to identify the best-quality proteome in any clade of interest, which is useful for selecting representative genomes and for improving annotation of nonmodel species.

### Assembly and annotation comparisons

OMArk can be used to compare gene repertoires from different assemblies or annotations of the same species, aiding in benchmarking annotation methods or gauging improvement in gene repertoire completeness and consistency over time. To illustrate, we ran OMArk on newer versus older assemblies or annotations for species with documented changes between the Ensembl Metazoa releases 53 and 54^17^. This corresponds to 11 protostome species with annotations on different assembly versions and seven nematode species with different annotations on the same assembly (Supplementary Table 5).

When comparing OMArk results across different annotation versions of the same assembly, we observed minor changes (less than 1% for most metrics), likely due to incremental annotation updates affecting few genes. Nevertheless, we still detected a trend toward fewer duplicated genes and more consistent genes (Supplementary Results: Assembly and annotation comparisons).

Comparing annotations on different assemblies, OMArk detects noticeable improvement in completeness and/or in structurally and taxonomically consistent genes for all but one species, but not always in both (Supplementary Results: Assembly and annotation comparisons; Supplementary Table 9). For instance, *B. impatiens* and *Acyrthosiphon pisum* showed a slight decline in completeness (−1.21% and −0.73%, respectively) but a large rise in taxonomically and structurally consistent genes (+17.34% and +21.06%, respectively; Fig. 6b). In contrast, *Crassostrae gigas* exhibited an increase in completeness (+4.38%) and a decrease in consistency (−9.16%).

OMArk also detected the removal or decrease in contamination for three species (*Schistosoma mansoni*, *A. pisum* and *Glossina fuscipes*), as well as new contamination introduced in *Teleopsis dalmanni’*s latest assembly. Most of the observed changes had no clear correlation with improvement in assembly quality metrics, except the proportion of fragmented genes decreasing with a higher N50 (Pearson correlation: 0.85, *P* value: 0.002). Our results indicate that new assemblies generally improved gene set quality, changed contamination status and reduced fragmented gene models due to higher assembly contiguity. However, these new assemblies were not necessarily annotated in the same way, making it difficult to discern whether observed changes are due to improved assemblies or to improvements in annotation procedures.

Finally, we compared 1,200 pairs of protein-coding gene annotations, each pair including one annotation from Ensembl and the other from the NCBI (GenBank and RefSeq), both derived from the same assembly. We analyzed the differences in OMArk and BUSCO results for all these pairs of annotations (Supplementary Tables 6 and 8 and Supplementary Fig. 32). NCBI proteomes generally exhibited higher completeness (+1.39%), fewer proteins with no known homologs (−0.64% unknown) and fewer structurally inconsistent proteins (−0.18% partial mapping and −0.64% fragments). Conversely, Ensembl proteomes exhibited a slightly lower taxonomic inconsistency (−0.09%).

Because OMArk’s underlying OMA database predominantly sources its proteomes from Ensembl (74% of Eukaryotic proteomes, Supplementary Fig. 31), we hypothesized this might introduce a bias. We tested this by comparing results on a subset of annotation pairs from species in OMA sourced from Ensembl to the rest of dataset. In this subset, proteomes from Ensembl had fewer detected fragments (−0.27%), fewer partial mapping proteins (−0.28%) and fewer taxonomically inconsistent proteins (−0.28%) than NCBI proteomes. These differences confirm that OMArk is slightly biased due to the reference proteomes’ origin. Thus, NCBI proteomes may appear slightly worse than they actually are, not necessarily due to quality issues but due to discrepancies in gene models predictions compared to Ensembl. However, the quantitative impact of such bias is minimal and unlikely to obscure any major annotation quality issues.

Overall, our findings highlight OMArk as a valuable tool for tracking improvements in genome assembly and annotation. By analyzing other metrics beyond completeness, OMArk can detect changes toward overall better gene sets, even when the completeness decreases. Furthermore, OMArk is effective for comparing different methods or sources of annotation, although users should note that minor differences between proteomes could be attributed to a bias induced by OMArk’s reference proteomes.

## Discussion

OMArk, leveraging the OMA database and *k*-mer-based fast gene family placement, evaluates the quality of protein-coding gene annotations. Our results on simulated incomplete genomes and on real proteomes demonstrates OMArk’s completeness measure is comparable to BUSCO. This finding is not surprising, as both methods assess the presence or absence of near universally conserved genes in a lineage. However, there are several key differences. OMArk not only focuses on single-copy conserved genes but also includes gene families that underwent duplication. Second, BUSCO uses hidden Markov model profiles to map query genes to their conserved gene families, a method more accurate but slower than the *k*-mer mapping exploited by OMArk. Finally, OMArk does not rely on a prespecified dataset of conserved genes but automatically chooses them depending on the query species’ taxonomic lineage.

OMArk assesses proteome consistency using a broader selection of orthologous groups than conserved ones, and proteins placed into gene families that are taxonomically consistent with the species of interest can be more confidently considered as true coding genes. Moreover, we can assess the quality of their gene structures by comparing to known sequences in the same family. However, there are a few caveats when interpreting OMArk results: gene consistency with the same lineage is expected only in species with predominantly vertical gene family inheritance and if the chosen family is well sampled and of good quality in our reference database.

Like most orthology databases, OMA, OMArk’s reference database, has uneven taxonomic sampling. For instance, mammals are overrepresented relative to total biodiversity, whereas free-living unicellular eukaryotes are underrepresented. OMA is actively maintained and has a release cycle of under a year, focusing on improving coverage for underrepresented species while including only high-quality data. Consequently, OMArk’s resolution is expected to improve as more diverse genomes are included. When choosing a reference lineage, OMArk selects the most specific clade with a sufficient number of species. However, an excessively broad clade may lack accuracy (most genes being consistent and few genes needed for completeness), whereas an excessively narrow clade may not be generalizable. OMArk issues warnings for ancestral lineages at the genus level or below and the phylum level and above and allows users to select the taxonomic rank for an ancestral lineage. We recommend that users be mindful of OMA’s species coverage for the ancestral lineage and interpret the results critically in this context.

OMArk’s completeness and consistency metric assume that proteomes in OMA accurately reflect the ‘real’ gene content of the species in the clade, which may not always hold true beyond a few highly curated species. Any proteome will likely carry bias from its annotation method. Such a bias impacts OMArk because many eukaryotic proteomes in OMA were downloaded from Ensembl. Although these are high-quality annotations, OMArk consequently is slightly biased towards Ensembl’s and similar gene prediction pipelines. Comparisons with NCBI show that some proteins not predicted by these pipelines will appear as inconsistent, and other valid gene structure predictions may be classified as partially mapping or fragmented. Although this effect is minor, users should be careful of such bias when comparing annotations. Finally, OMArk reports possible contamination, which should help genome annotators to flag contamination cases and reassess their genomic data. However, users should be aware of a few caveats. OMArk has a low sensitivity to contamination from human sequences or from eukaryotes from lowly sampled clades, and it is limited to coding regions. Furthermore, OMArk cannot discriminate between contamination and recent horizontal gene transfer. Using the list of potential contaminants, annotators can identify the corresponding contigs in the genome assembly for validation. Nevertheless, we recommend using assembly-level dedicated methods^18^ such as BlobToolKit^19^ to perform in-depth analysis and correction of genome assemblies.

OMArk provides a comprehensive proteome quality assessment, aiding annotators in improving gene annotation and enabling users to select high-quality proteomes for their investigations. We hope OMArk will help improve the quality of existing and newly produced gene sets, advancing the field of genomic research.

## Methods

### OMAmer placement

The OMAmer database used in this study was generated from the November 2022 release of the OMA database^10^. Placements were made with OMAmer version 2.0.0, using default parameters. Root HOGs (that is gene families) with five or less proteins and a species coverage (the proportion of species in the clade with a gene in the HOG) lower than 0.5 were excluded, as they are most likely spurious.

### Overview of the OMArk algorithm

All analyses shown here were performed with OMArk version 0.3. The OMArk software takes the following as minimum input: 1) the output of the OMAmer placement for a whole proteome, whereby proteins of these proteomes are placed in HOGs, and 2) the path to the corresponding OMAmer database.

Optionally, OMArk can take the NCBI taxonomy ID of the proteome’s species which will be used to select its ancestral lineage; otherwise, its taxa will be inferred automatically (see 'Automatic species identification and contamination assessment' below). The FASTA file of the query proteome is also an optional input, which may be used to generate output FASTA files for inconsistent, contaminant and unknown proteins. Finally, if the proteome contains multiple isoforms per gene, an additional option (-i) allows the user to provide a comma-separated file where all protein identifiers corresponding to a single gene are written on the same line. Only one isoform will be selected for completeness and consistency assessment, based on the OMAmer placement score as detailed in the section ‘Isoform selection’ below.

#### Isoform selection

If the target proteome contains more than one protein by gene, and an isoform file was provided by the user, OMArk will automatically select the sequence with the best match in the OMAmer database. This selection is based on the hit’s ‘family *P*’ (from OMAmer), which represents the negative natural logarithm of the *P* value of having as many or more *k*-mers in common under a binomial distribution. This helps ensure that gene model comparison will happen between similar isoforms. OMArk selects the isoform with the lowest *P* value as the isoform of reference. The list of selected isoforms is then provided in OMArk’s output in a file with the suffix _selected_isoform.txt.

#### Automatic species identification and contamination assessment

The taxonomic distribution of HOGs in the query proteome can be used to automatically detect the species from which they come from. OMArk does this by using the nonredundant list of HOGs in which proteins of the query proteomes were placed and extracting the taxonomic level where each HOG is defined (that is, the taxonomic node after emergence or duplication of the gene family). This step is used to obtain the number of mappings to each clade in the tree of life, which we call clade occurrence *N*.

To reduce noise due to incorrect mapping, which is more common in broad clades with a large number of HOGs, we divide the clade occurrence by the number of total HOGs defined at each level to obtain a normalized clade occurrence *N*. In the presence of only one species and no noise, we assume the most likely placement would be the clade with the highest normalized clade occurrence, with all its parent clades having an equal or lower normalized occurrence count.

The OMArk algorithm uses this assumption and implements a few corrections to account for noise in HOG placement and allow for more than one species in the proteome, in case of contamination. First, all clades with an occurrence of more than two are used to construct a simplified taxonomic tree containing only branches leading to these clades. The tree structure itself is derived from the OMA underlying taxonomy, which used until now the NCBI taxonomy^20^.

The OMArk algorithm for species identification is a recursive postorder traversing function. At each leaf, it returns the leaf clade as likely placement, with occurrence *N*_leaf_ and normalized occurrence *N*′_leaf_ of the taxonomic level. At each node, it compares the occurrence scores of the current node to the most likely placements of its children. To be considered relevant, a child’s placement has to satisfy:

- ${N}_{{{\mathrm {child}}}} > {N}_{{{\mathrm {node}}}} \times {D}_{{{\mathrm {node}}},{{\mathrm {child}}}}$, where *D*_*x,y*_ represents the proportion of HOGs defined in clade *x* that have a child defined in clade *y*. A high value represents a high duplication rate in the branch leading from *x* to y. This condition controls for high duplication numbers in the branch leading to some lineages (for example, ancestral WGD), which favor overspecific placement into those clades.
- ${N^{\prime}_{\mathrm {child}}} > {N^{\prime}_{\mathrm {node}}} \times \vert {S}_{{{\mathrm {child}}}} \vert / \vert {S}_{{{\mathrm {node}}}} \vert$, where | *S*_*x*_*|* is the number of species in clade *x*. This condition controls for sampling imbalance in lineages that favor overspecific placement in larger clades.

If only one child is considered relevant, it is returned as the most likely taxon. If more than one is considered relevant, all are returned as likely taxa. If no child is considered relevant, only the current node is returned as likely taxon. After traversal, this module outputs a list of independent clades which have more hits than expected by chance.

For each clade with more placements than expected, we select all proteins that can be unambiguously attributed to these clades (that is, all proteins that map to a HOG defined at a node in the subtree leading to its first common ancestor with any other clade in the list). The clade with the most proteins is considered as the most likely main taxon and the other as contaminants. In the case when OMArk detects multiple possible contaminant species for a protein based on its placement, it will report the protein as ‘ambiguous contaminant’ sequences. This feature possibly overestimates the proportion of contaminant sequences, especially in presence of spurious hits (that would otherwise be in the inconsistent category), but ensures most of the contaminants are included in the category.

#### Completeness assessment

The completeness assessment measures the proportion of HOGs that are expected to be conserved in the species’ lineage. This assessment is done by first selecting the ancestral lineage of the species, defined as the most recent taxonomic level including the species and represented by at least five species in the OMA database. Then, OMArk defines the ancestral ‘conserved repertoire’ of the query species: all the HOGs defined at this ancestral level that cover more than 80% of the species in the clade.

Because a HOG at the selected taxonomic level represents a single ancestral gene, conserved HOGs are classified as one of the following:

- Single copy if one protein in the query proteome maps to it. To be robust to minor errors in phylogenetic placement, a single underspecific hit (placement in a parent HOG; Fig. 2a) or a single overspecific hit (placement in a child HOG) is sufficient to consider a conserved HOG as single copy.
- Duplicated if more than one query protein maps to it. A duplicated, conserved HOG is further classified as unexpected if multiple proteins are all placed into the ancestral HOG itself (that is, no evidence of such duplication exists in the OMA database) or expected if the proteins were placed into subfamilies of the HOG (that is, the duplication event is documented in the database).
- Missing if no proteins in the query genome are placed into it.

#### Consistency assessment

The consistency assessment evaluates the query proteome quality, again depending on the placement of its proteins into HOGs and the taxonomic level at which these HOGs are defined. Here, OMArk uses a ‘lineage repertoire’ of the query species: all the HOGs from the conserved ancestral repertoire plus those that originated later on and are still present in at least one species of the lineage. It uses this lineage repertoire to classify proteins as:

- Unknown proteins are those that were not placed into existing HOGs. They correspond to either errors in the annotation or to gene families with no detectable counterpart in OMA (due to falling in sparsely sampled clades or being a novel protein).
- Consistent proteins are those that were placed into a HOG consistent with the reference lineage: the HOG has a representative of at least one species from the lineage, whether it was present in the common ancestor of the reference lineage or emerged in its descendants.
- Contaminant proteins are those that map to a lineage of another species which has been detected as a likely contaminant by the contaminant detection module of OMArk (see ‘Automatic species identification and contamination assessment’ in Methods).
- Inconsistent proteins are those that were placed into HOGs from other parts of the tree of life and for which there is no evidence the gene families existed in the selected lineage or in any contaminant lineage. They are likely to correspond to gene families that were not observed in those species before or to be incorrect protein sequences.

For the proteins that map to existing HOGs, an additional characterization is provided:

- Partial mappings are proteins from which less than 80% of the sequence overlaps with their target root HOG, that is, at least 20% of the sequence at the extremity of the protein has no *k*-mer in common with the root HOG.
- Fragments are sequences that are not partial hits but whose length is <50% of the median length of sequences in the HOG it was placed to.

### Acquisition of proteome data

Reference proteomes were downloaded from UniProtKB^8^ on 10 February 2022 (version April 2021). Assembly data for this dataset were kindly provided by the UniProt Reference Proteome team and are available in Supplementary Table 3. Ensembl Metazoa proteomes were downloaded from their ftp website from version 52, 53 or 54 of the database (version number is reported in Supplementary Table 5). Data for the comparison of Ensembl and NCBI annotations were downloaded separately for each database. Ensembl proteomes were downloaded from Ensembl FTP for version 110 of the Main Ensembl website and version 57 of Ensembl Plants, Ensembl Metazoa, Ensembl Fungi and Ensembl Protists. NCBI proteomes were downloaded via the NCBI Datasets python API in August 2023, requesting genomes with annotation and downloading GFF and proteins files. Isoform files were generated for the Ensembl proteomes using the gene information in FASTA header: NCBI isoforms files were created using the gene and protein information in the corresponding GFF files.

### Generation of simulated proteomes

Two datasets were used to assess the effect of introducing errors into proteomes on OMArk quality scores. These two datasets of real proteomes were used as the basis of the simulation: model proteomes and taxonomically representative proteomes. Model proteomes correspond to model eukaryotic species whose proteomes are assumed to be of high quality. Representative proteomes were selected under several criteria: they represent the major eukaryotic taxonomic divisions (two of each when possible), they must not be present nor have species of the genus represented in the OMA database (to avoid circularity) and they must have the best score possible for the aspect of OMArk quality measures (mainly, few missing genes and a higher proportion of consistency from other species of their division). Both lists of species are available in Supplementary Table 1.

These source proteomes were manipulated in six ways, each simulating a case of spurious annotation:

- *Missing genes.* For each proteome, only a fraction of the proteins were kept at random. This was repeated independently ten times with different proportions of the proteome kept, from 10% to 90% by increments of 10%.
- *Erroneous sequences*. Errors in gene annotation were simulated from randomly generated nucleic sequences, from an equiprobable distribution of each base (25% of chance to draw A, T, G and C). The sequences were generated by increments of three, representing codons in the open reading frame, until a stop codon appeared. The resulting sequences were then translated into proteins and kept if their length was more than 20 amino acids. These sequences, independently generated in each simulation, were then added to each proteome proportionally to the original number of proteins in it, from 10% to 90% by increments of 10%.
- *Amino acid distribution-aware erroneous sequences*. More realistic erroneous sequences were generated by computing the empirical distribution of amino acid in all proteins of the source proteome (treating stop codons as an additional amino acid) then sampling random characters from this distribution. This generated proteins with similar amino acid distribution and average length as the proteins in the target proteomes. These sequences, independently generated in each simulation, were then added to each proteome proportionally to the original number of proteins in it, from 10% to 90% by increments of 10%.
- *Fragmented sequences*. We simulated fragments in the proteome by selecting random proteins in it, and removing part of the sequences randomly from between 10% and 90% of its length. The part was removed from either the C-terminal or N-terminal end, randomly with equal probability of each. This was repeated with different sequences until a target proportion of the proteome size. This process was done independently ten times for each proteome, for 10% to 90% of the proteome, by increments of 10%.
- *Fused sequences*. We simulated fused protein sequences by randomly selecting pairs of proteins in the proteome and appending them to one another: Before the simulated fusion, we removed between 0% to 20% of one protein at the C-terminal end and 0% to 20% of the other at the N-terminal end. The merged protein was added to the proteome while the two original proteomes were removed. This process was repeated until a target proportion of the proteome size was reached. This step was done independently ten times for each proteome, for 10% to 90% of the proteome, by increments of 10%.
- *Contamination*. A list of eukaryotic and bacterial proteomes, either from common contaminants in genomic data or microscopic species from a variety of clades were selected as contaminant proteomes. Then, a fixed number of proteins (10, 20, 50, 100, 200, 500 and 1,000) were drawn randomly without replacement independently from the contaminant proteomes and added to the complete source proteomes.

### BUSCO comparisons

BUSCO^3^ v.5.2.2 was run on UniProt Reference Proteomes and simulated data, using the odb10 version of the BUSCO dataset of the most specific lineage possible covering the target proteome and with default options. The corresponding dataset is available in Supplementary Table 3. The summarized result for the BUSCO run was then extracted from the summary file.

### Avian proteome fragment analysis

To compare fragmented sets between all avian UniProt Reference Proteomes, we first queried the OMArk results for the proteins classified as lineage consistent fragments and then used the OMAmer placement file to obtain the gene families to which they were associated. To avoid biasing the comparisons in the presence of duplication, we associated each gene name to their whole gene family identifier (root HOG) rather than to their subfamilies. The overlap between fragmented gene sets between two species was computed by directly comparing sets of their associated gene families using the following formula, for two sets *A* and *B*: $\frac{\vert A \cap B \vert}{\min(\vert A \vert,\vert B \vert)}$. The denominator was chosen to be the cardinality of the smallest set in order to not underestimate overlap in the smallest sets.

The zebra finch proteome was downloaded from the Ensembl archive for version 85 of the database. Overlap with the zebra finch taxonomically consistent fragment set was done as above but using the cardinality of the target proteome’s fragmented gene family set as the denominator.

### Comparisons of NCBI and Ensembl proteomes

OMAmer and OMArk were run using FASTA and isoform files as input, along with the NCBI taxid of the proteome. We ran BUSCO with a FASTA file and the odb10 version of the BUSCO dataset, selecting the most specific lineage possible that covers the target proteome, and applied default parameters. Proteomes were matched to an assembly using either metadata downloaded from NCBI or downloaded from a species information file available on the Ensembl website. For NCBI and Ensembl proteomes with matching assemblies, we made pairwise comparisons for each OMArk value in the completeness and consistency assessment. When a species had an available proteome in OMA sourced from Ensembl, as per the OMA November 2022 release information, we marked these accordingly to assess bias in OMArk results.

### Runtimes of BUSCO and OMArk

BUSCO v5.2.2 and the OMArk pipeline (OMAmer v2.0.0 and OMArk v0.3.0) were run on the 1,805 UniProt Reference proteomes using a Snakemake pipeline^21^ and a Slurm scheduler. BUSCO and OMAmer were run with default parameters. We launched OMArk with a taxonomic identifier and an optional FASTA file. We ran BUSCO in offline mode with the required lineage folders available locally. All software was configured to run in serial mode, using only one thread. We obtained the job performance, including CPU time for each software on each proteome, from the Slurm scheduler efficiency report with the ‘seff’ command on each Slurm job. All computation was performed on the UNIL high-performance computer Curnagl, a 96-node cluster based on AMD Zen2/3 CPUs. 15 GB memory was requested for OMAmer; 10 GB for OMArk and 25 GB for BUSCO, which was enough to avoid any out-of-memory errors. All data were read and written on an 150 TB SSD-based scratch system.

### Additional analysis

The additional analyses were performed in Python (v. 3.9.5) within a Jupyter Notebook. Plots were created using the matplotlib (version 3.4.2)^22^ and the Seaborn^23^ (v0.11.2) libraries. Notebooks used for this paper are available in the associated Zenodo archive. The Notebook “Human_missing_genes.ipynb” is for investigating human genes deemed as missing and the Notebook “blobtoolkit_contamination_check.ipynb” is for validating OMArk contamination results with BlobToolkit. Other companion Notebooks are available on OMArk GitHub repository, in the utils folder. “Contextualize_OMA.ipynb” allows investigation of OMArk’s missing and fragmented genes using OMA public data (sequences and synteny) and provides instructions to perform assembly completeness assessment. The Notebook “Explore_Data.ipynb” allows visualization of many OMArk results at once.

### Reporting summary

Further information on research design is available in the Nature Portfolio Reporting Summary linked to this article.

## Online content

Any methods, additional references, Nature Portfolio reporting summaries, source data, extended data, supplementary information, acknowledgements, peer review information; details of author contributions and competing interests; and statements of data and code availability are available at 10.1038/s41587-024-02147-w.

## Supplementary information

Supplementary Information Supplementary Analyses. Supplementary Tables 7–9 and Supplementary Figures 1–56.

Reporting Summary

Supplementary Tables Supplementary Tables 1–6.

## Source data

Source Data Figs. 1–6 Table 1. Source data Fig. 1. OMArk results for *Danio rerio*. Table 2. Source Data Fig. 3. OMArk results for simulation of six species (full results in Supplementary Table 1. Table 3. Source Data Fig. 4. OMArk results for 1,805 UniProt species (full results in Supplementary Table 3). Table 4. Source Data Fig. 5. OMArk and BUSCO results and fragments proportion (full results in Supplementary Table 3). Table 5. Source Data Fig. 6.OMArk results for Myomorpha UniProt proteomes and for *Bombus impatiens* Ensembl Metazoa proteomes (full results in Supplementary Tables 3 and 5).

## Data Availability

UniProt Reference proteomes were downloaded from UniProtKB on 1 February 2022 (version 04/2021) through their ftp server. Ensembl Metazoa proteomes were downloaded from their ftp website from version 52, 53 and 54. NCBI proteomes were downloaded in August 2023 through the NCBI datasets python library, and proteomes from Ensembl 110 and Ensembl databases 57 were downloaded through their respective ftp websites. All datasets used and generated during the study and Supplementary Table files are made available through Zenodo (10.5281/zenodo.10034236)^24^. Precomputed results for UniProt, GenBank and Ensembl are made available through the OMArk web server (https://omark.omabrowser.org). Source data are provided with this paper.

## Code availability

OMArk is available on GitHub (https://github.com/DessimozLab/OMArk) and as a python package on PyPI and Anaconda. OMArk version 0.3.0 and OMAmer version 2.0.0 that were used for all analyses are available on Zenodo (10.5281/zenodo.10474466)^25^.
