# Supplementary material for: Quality assessment of gene repertoire annotations with OMArk
Source: Nat Biotechnol. 2024 Feb 21;43(1):124–33. doi: 10.1038/s41587-024-02147-w (PMC11738984; doi:10.1038/s41587-024-02147-w)
Supplement: Supplementary file 1 — Supplementary Analyses. Supplementary Tables 7–9 and Supplementary Figures 1–56. [file 41587_2024_2147_MOESM1_ESM.pdf]

nature biotechnology

Article https://doi.org/10.1038/s41587-024-02147-w

Quality assessment of gene repertoire annotations with OMArk

In the format provided by the authors and unedited

Supplementary Tables **2**
Supplementary Results **3**
Simulation results 3
Incompleteness simulation 3
Erroneous sequences simulation 6
Fragmented sequences simulation 9
Fused sequences simulation 10
Contamination simulation 12
Results on UniProt Reference Proteomes 13
Global results 13
Contamination detection and validation 15
BLAST cross-validation 16
BlobToolkit cross-validation 16
Comparison with BUSCO on UniProt Reference Proteomes 16
OMArk runtime assessment and comparison with BUSCO 20
Case studies of proteomes with outlier OMArk values 22
Analysis of avian proteomes 23
Comparison of proteomes from closely related species 27
Human and Hominidae proteomes 27
Mouse and Myomorpha 29
Gallus gallus and Galloanserea 30
Xenopus and Amphibia 31
Zebrafish and Otophysi 32
Drosophila melanogaster and melanogaster subdivision 33
Caenorhabditis elegans and the Caenorhabditis genus 34
Saccharomyces cerevisiae and Saccharomycetacea 35
Arabidopsis thaliana and Brassicaceae 36
Impact of the source of OMA’s proteomes on OMArk results 37
Global comparison of Ensembl and NCBI annotations 37
Danio rerio’s case studies 40
Assembly and annotation comparisons 42
Assembly comparisons 42
Annotation comparisons 56
Investigation of human missing genes 64
Supplementary references **67**

Supplementary Tables

The Supplementary Tables are available at Zenodo (10.5281/zenodo.10034236).

Supplementary Table 1. **Source proteomes used for simulations**. List of the proteomes used as a source proteome in the simulation, divided by dataset. The “Model” dataset corresponds to common model organisms. The “Representative” dataset corresponds to species from across the eukaryotic taxonomy, with two species per each major lineage. Also given is data about the ancestral lineage used in OMArk for each proteome and the number of conserved HOGs, as well as equivalent data in BUSCO.

Supplementary Table 2. **Contamination detection by OMArk on simulated contaminated proteomes**. The Summary (top) indicates the percentage of proteomes in which contamination was detected for the Model and Representative datasets, by contaminant species. Each table at the bottom of it, one for each degree of contamination, details for each simulated proteome what contaminant was detected by OMArk, and recapitulates the proportion of the dataset where the detection is accurate - as in the summary sheet.

Supplementary Table 3. Global OMArk and BUSCO results on the UniProt Reference Proteome data. Each row represents a proteome, its OMArk statistics, and BUSCO statistics. All of OMArk and BUSCO statistics are given in percentage. Assembly length, N50 and date of submission are also given.

Supplementary Table 4. **Detected contamination events and validation**. List of all contaminations detected in the 1805 UniProt Reference proteomes: the proteome and species in which it was detected, the contamination source clade, and the corresponding number of unambiguously associated proteins. Also included are the BLAST and BlobToolKit validation results of detected contaminants.

Supplementary Table 5. **OMArk results for Ensembl Metazoa proteomes with recent changes in assembly or annotation**. Each row represents a proteome, its version number, and its OMArk statistics. All of OMArk statistics are given in percentage. Assembly length, N50, and date of submission are also given.

Supplementary Table 6. Comparison of OMArk results for Ensembl and NCBI proteomes that share the same assembly. Each row represents one assembly, with the source of annotations for each database, and the corresponding OMArk statistics. All of OMArk statistics are given in percentage. Assembly length, N50, and date of submission are also given.

Supplementary Results

Simulation results

In order to evaluate the ability of OMArk to provide accurate quality assessment, we simulated cases of genome incompleteness, presence of erroneous sequences (including spurious sequences, fusions and fragmentations), and cross-species contamination on two datasets of eukaryotic proteomes. The first dataset is composed of 9 model species' proteomes expected to be of high quality due to extensive curation (**Model dataset**). The second dataset is composed of 16 proteomes representing the diversity of Eukaryotes with no presence in the original OMA database, which mirrors the expected use case (**Representative dataset**).

Incompleteness simulation

For our sets of 25 complete proteomes, we removed proteins at random from each proteome to simulate from 10% to 90% completeness, by increments of 10%. Results for all simulated proteomes are shown in Supplementary Figure 1 (Model dataset) and Supplementary Figure 2 (Representative dataset). Given that source proteomes, especially from the Representative dataset, do not all have 100% completeness to start with, we consider the expected completeness as the measured completeness of the source proteome multiplied by the simulated completeness. For instance, a source proteome that is 90% complete would have an expected completeness of 9% for the 10% simulation.

For the Model dataset, both OMArk and BUSCO [1] were able to accurately assess completeness, although with a slight overestimation (+2.3% on average and +2.1% on average, respectively; Supplementary Figure 1). Generally, OMArk reports more duplicated genes than BUSCO. This is expected, as OMArk uses a set of conserved genes expected to be in single-copy in the ancestral repertoire, but not necessarily in single-copy in most species, contrary to BUSCO. There were a few differences between methods, depending on the species. For example, BUSCO gives, on average, a close estimate of completeness for *Caenorhabditis elegans* (+0.5%), while OMArk overestimates it by a higher margin (+3.2%). The opposite relationship was observed for *Danio rerio* (+2.5% for OMArk, +4.6% for BUSCO).

For the Representative dataset, the overall accuracy was worse for both methods , although BUSCO overestimated completeness by a smaller margin than OMArk (overestimation of +9.9% for OMArk, +6.1% for BUSCO; Supplementary Figure 2). For all but 4 proteomes, even though slightly overestimated, the detected completeness scales in a linear manner with the actual completeness. However, for *Fistulifera solaris*, *Panicum miliaceum*, *Stentor coeruleus* and *Hibiscus syriacus,* the detected completeness is always noticeably overestimated and grows logarithmically with the actual completeness. This pattern may be explained by the high degree of duplication in these species due to polyploidy [2–5]. Thus, two or more genes would need to be lost for an ortholog to be considered as missing. Although OMArk is especially sensitive to this effect, BUSCO is also affected to some extent. These results point to a limitation of using "core gene sets"-based methods like OMArk and BUSCO for completeness evaluation in polyploid species. When excluding these outlier species, the average overestimate is +5.8% for OMArk and +3.14% for BUSCO for the representative dataset.

Overall, OMArk can reliably evaluate proteome completeness on simulated datasets, in most cases providing a realistic estimate. For these simulations, the estimates are close to, but slightly

worse than, the state-of-the-art BUSCO. In particular, OMArk is more sensitive to overestimation in the presence of many duplications in the source genome.

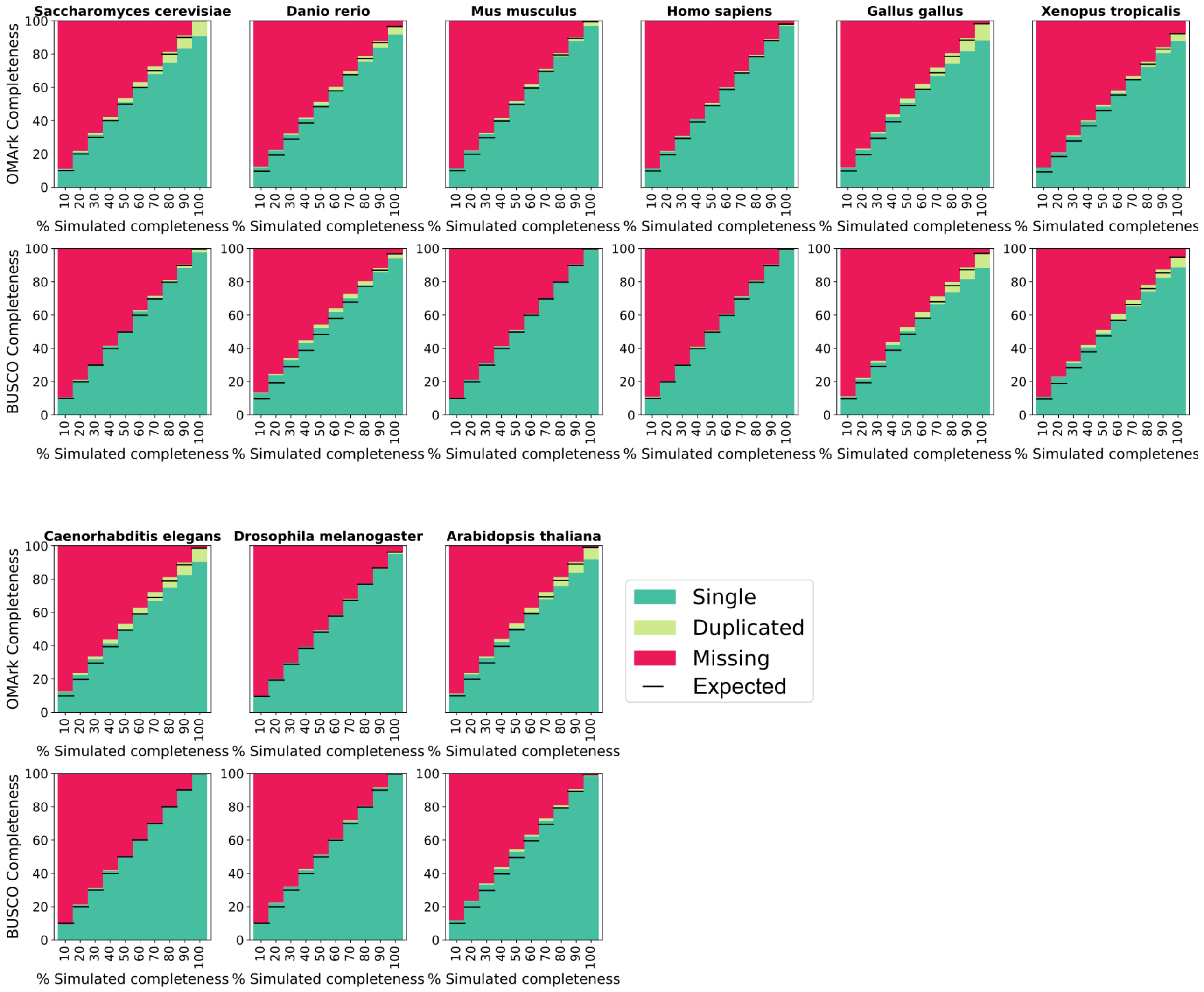

Supplementary Figure 1. **OMArk and BUSCO results for incompleteness simulations on the "Model dataset" proteomes**. Pairs of vertical squares show the completeness statistics (y-axis) for OMArk (top) and BUSCO (bottom). The species name corresponding to the source proteome is indicated on top of each pair of squares. Gene families (HOGs) present in single-copy (dark green), duplicated (light green), and missing (red) are shown for different levels of simulated completeness (x-axis). The dark horizontal lines show the expected completeness measurement (divide between the green and red section), considering the estimated completeness of the source proteome. The results from running BUSCO and OMArk on the Model dataset show that they accurately measure completeness, but tend to slightly overestimate it.

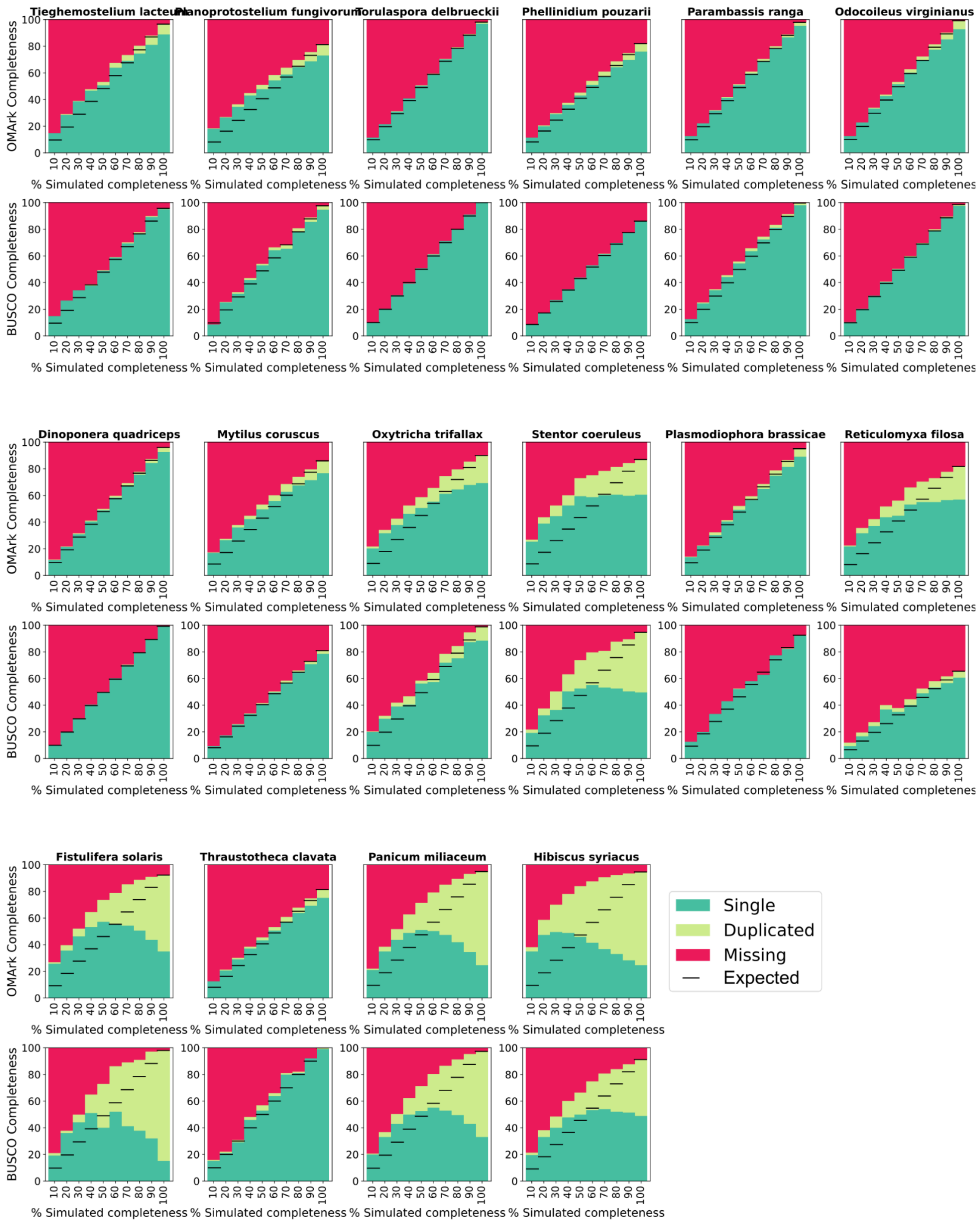

Supplementary Figure 2. **OMArk and BUSCO results for incompleteness simulation on the "Representative dataset" proteomes**. Pairs of vertical plots show the completeness statistics (y-axis) for OMArk (top) and BUSCO (bottom). The species name corresponding to the source proteome is indicated on top of each pair. Conserved gene families (HOGs) present in single-copy (dark green), duplicated (light green), and missing (red) are shown for different levels of simulated completeness (x-axis). The dark horizontal lines show the expected completeness measurement (divide between the green and red section), considering the estimated completeness of the source proteome. BUSCO and OMArk accurately report completeness level in most cases, but overestimate it greatly in case of highly duplicated proteomes (polyploids).

Erroneous sequences simulation

For each proteome, we added protein sequences translated from random nucleic sequences to simulate proteins translated from randomly occurring open reading frames in the genomic sequence. The number of random sequences was proportional to the number of proteins in the source genome, from 10% to 90% by increments of 10%. All results are shown in Supplementary Figures 3 (Model dataset) and 4 (Representative dataset).

In both Model and Representative datasets, the proportion of Unknown proteins increases noticeably with the proportion of added erroneous protein sequences (on average, +45.4% of Unknown genes in the 90% added error simulation in the Model dataset, +35.3% in the Representative dataset).The relative increase of Unknown proteins is lower in the Representative dataset since these proteomes from lower sampled lineages initially have a high amount of sequences that are not present in the underlying OMA database. For example, in *Plasmodiophira brassicae*, the percentage of sequences OMArk detects as Unknown starts at 34.6% with no simulated error added, and is at 65.5% Unknown at with 90% error added, an absolute increase of only +30.9%. On the other hand, the proportion of both Inconsistent and Consistent genes decreases with the addition of errors, which is expected as the total proteome size increases with added error. Importantly, the number of taxonomically and structurally Consistent proteins (non-hashed blue bars in Supplementary Figures 3 and 4) stays identical, regardless of the proportion of added error (expected level in all simulations is shown as a red bar). This effect is observed in both Model and Representative species, indicating the OMArk is efficient at detecting dubious proteins.

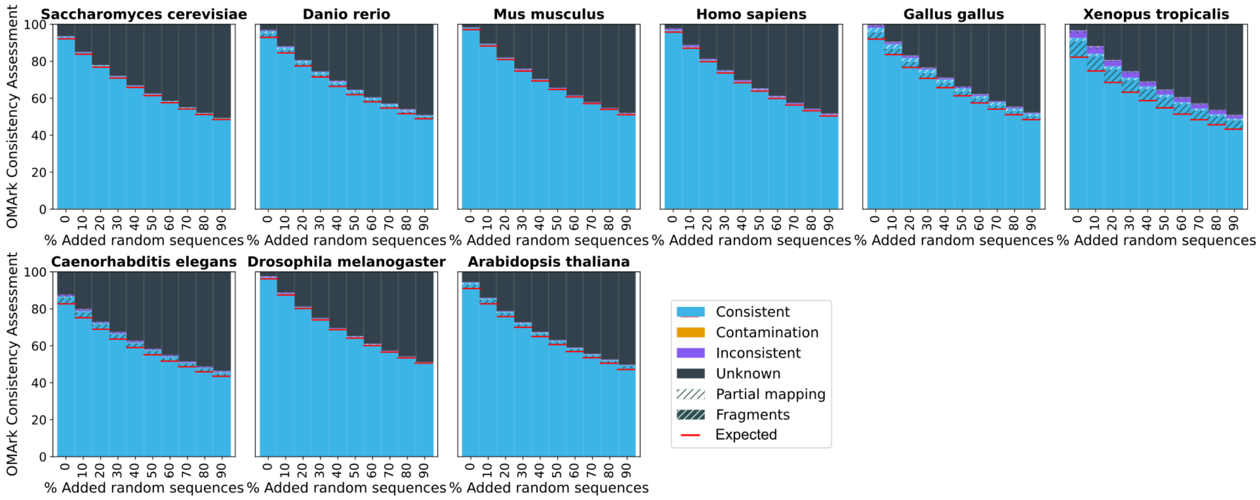

Supplementary Figure 3. OMArk results for random erroneous sequence simulation on "Model" proteomes. Squares represent OMArk statistics for consistency assessment using different proportions of randomly generated proteins in the source proteome, as a proportion of its original size. The species name of the source proteome is indicated on top of each square. The red lines show the expected proportion of taxonomically and structurally consistent genes in the source proteomes of the dataset. The proportion of taxonomically and structurally consistent genes (non-hashed blue) stays virtually identical, regardless of the amount of noise added.

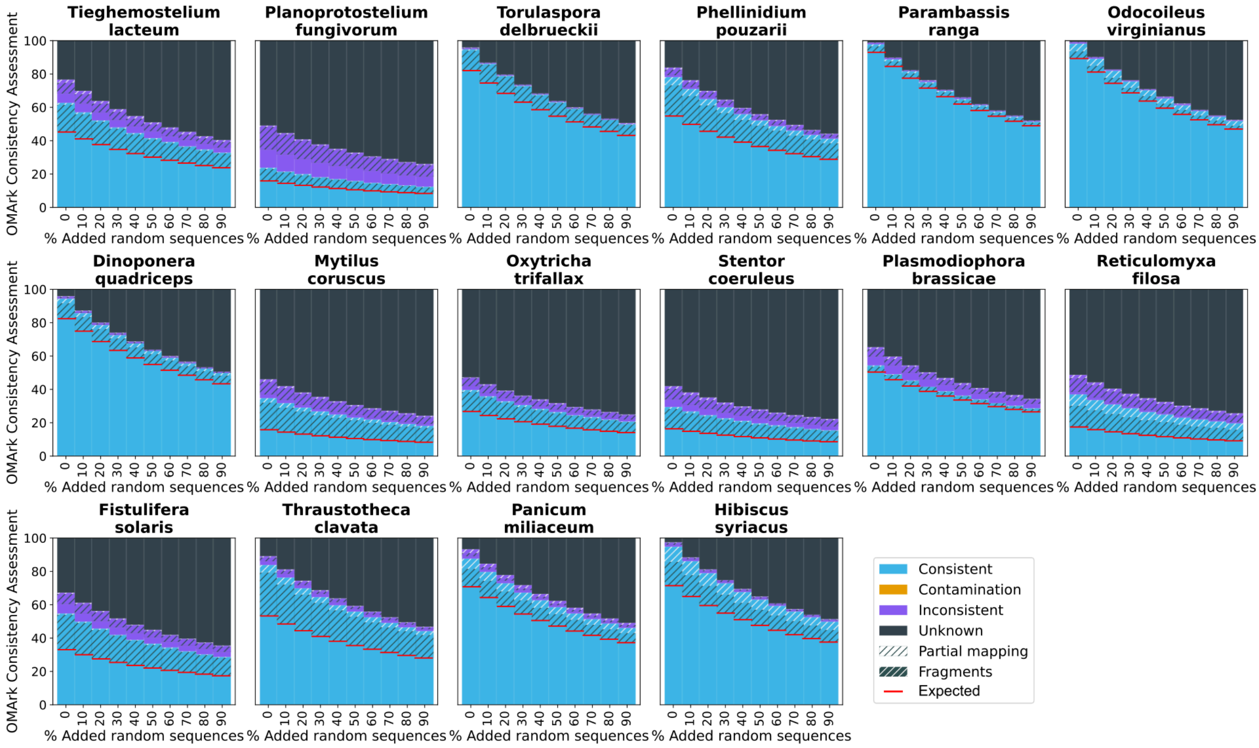

Supplementary Figure 4. **OMArk results for random erroneous sequence simulation on "Representative" proteomes**. Squares represent OMArk statistics for consistency assessment using different proportions of randomly generated proteins in the source proteome, as a proportion of its original size. The species name of the source proteome is indicated on top of each square. The red line shows the expected proportion of taxonomically and structurally consistent genes in the source proteomes of the dataset. The proportion of taxonomically and structurally consistent genes (non-hashed blue) stays virtually identical, regardless of the amount of noise added.

As proteins translated from random nucleic sequences may not be accurate representations of mistakes made by gene prediction software, we generated another category of protein sequences in which amino acids were drawn randomly from the empirical distribution of amino acids in the proteomes they were added to. Again, the number of random sequences was proportional to the number of proteins in the source genome, from 10% to 90% by increments of 10%. All results are shown in Supplementary Figures 5 (Model dataset) and 6 (Representative dataset). The results for this simulation were essentially identical to the previous simulation, although with a slightly reduced increase of Unknown genes by added error (+45.3% of Unknown genes with 90% added error for the Model dataset and +35.2% for the Representative dataset). Again, the proportion of structurally and taxonomically consistent genes stayed identical regardless of the proportion of added errors.

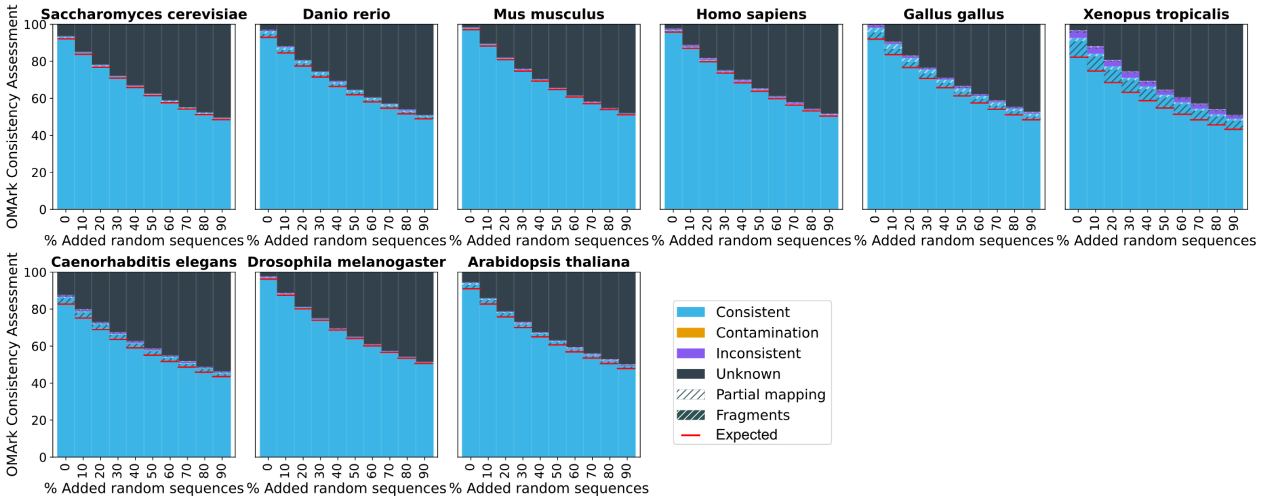

Supplementary Figure 5. **OMArk results for amino acid distribution-aware random erroneous sequence simulation on "Model" proteomes**. Squares represent OMArk statistics for consistency assessment using different proportions of randomly generated proteins in the source proteome, as a proportion of its original size. The species name of the source proteome is indicated on top of each square. The red lines show the expected proportion of taxonomically and structurally consistent genes in the source proteomes of the dataset. The proportion of taxonomically and structurally consistent genes (non-hashed blue) stays identical, regardless of the amount of noise added.

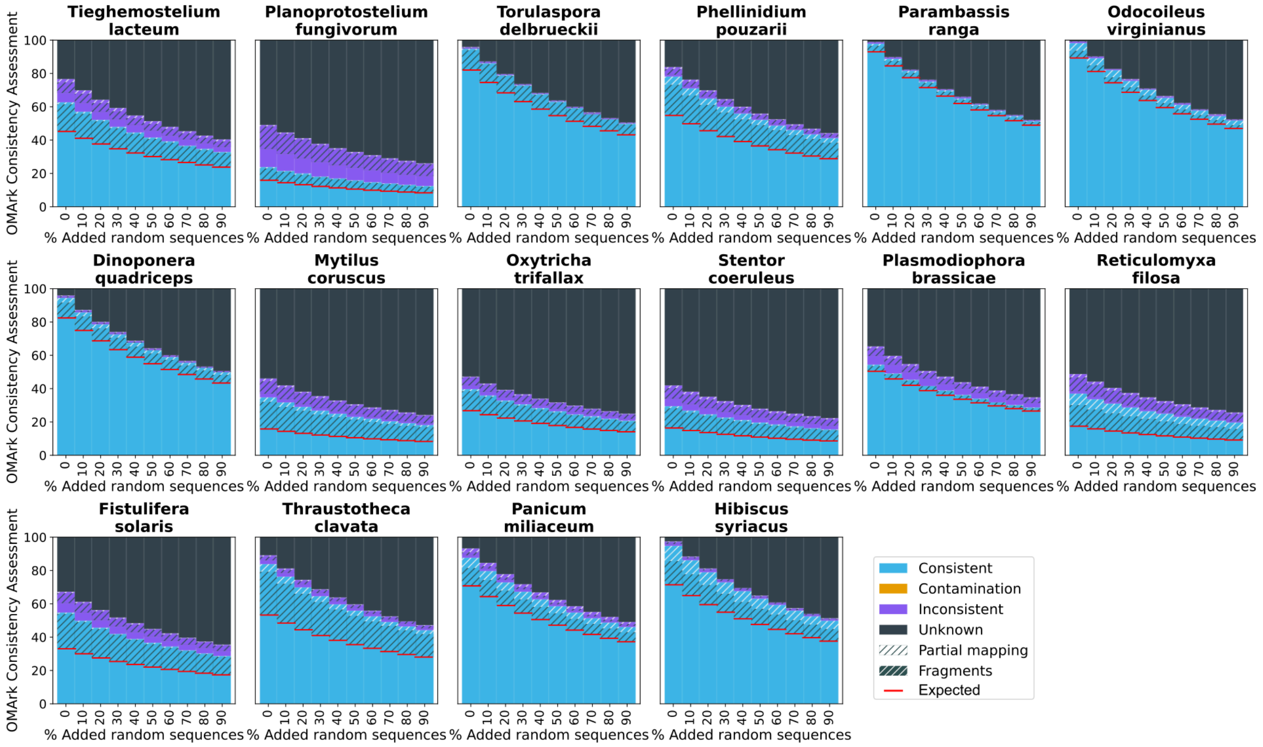

Supplementary Figure 6. **OMArk results for amino-acid distribution-aware random erroneous sequence simulation on "Representative" proteomes**. Squares represent OMArk statistics for consistency assessment using different proportions of randomly generated proteins in the source proteome, as a proportion of its original size. The species name of the source proteome is indicated on top of each square. The red lines show the expected proportion of taxonomically and structurally consistent genes in the source proteomes of the dataset. The proportion of taxonomically and structurally consistent genes (non-hashed blue) stays identical, regardless of the amount of noise added.

Overall, these results demonstrate that random sequences, or random ORFs, will not be placed with OMAmer and will be detected by OMArk as having no homologs. This simulation shows that the combination of simple metrics provided by OMArk regarding the protein-coding gene set allows one to discriminate noise from high confidence protein-coding genes.

Fragmented sequences simulation

For each proteome, we artificially fragmented proteins in the source proteomes. We selected random sequences and removed anywhere from 10%-90% of the sequence's length on either the N-terminal or C-terminal end. We did this from 10% to 90% of the proteome by increments of 10%. All results are shown in Supplementary Figures 7 (Model dataset) and 8 (Representative dataset).

The proportion of structurally fragmented sequences in both the Model and Representative datasets increased proportionally to the proportion of added fragments, but was systematically underestimated. For example, the average proportion of detected fragments was only on average 39.5% for 90% simulated fragments in the Model species and 17.6% detected fragments for 90% simulated fragments in the Representative species. The proportion of Unknown proteins also increased by 2.7% on average for the Model species and 10.1% for the Representative species, likely because fragmenting genes can lead to them not be recognizable as homologs, especially in lowly sampled species.

Although the proportion of taxonomically and structurally Consistent genes was above what was expected if all fragments were detected (red lines in Supplementary Figures 7-8), it was close to what would be expected if half of the fragments were detected (pink lines in the figures). The difference compared to the expectation in this case was on average a 0.6% underestimation in Model species and a 1.8% underestimation in Representative species. This result is logical since OMArk considers sequences as fragmented if they are half the size of the median sequence lengths for species in this family. In our simulated fragmentation procedure, we randomly removed anywhere from 10-90% of the protein length, thus only half should be detected as fragments.

These results demonstrate that OMArk is efficient at detecting small fragments of proteins as long as they are less than half the size of their expected length.

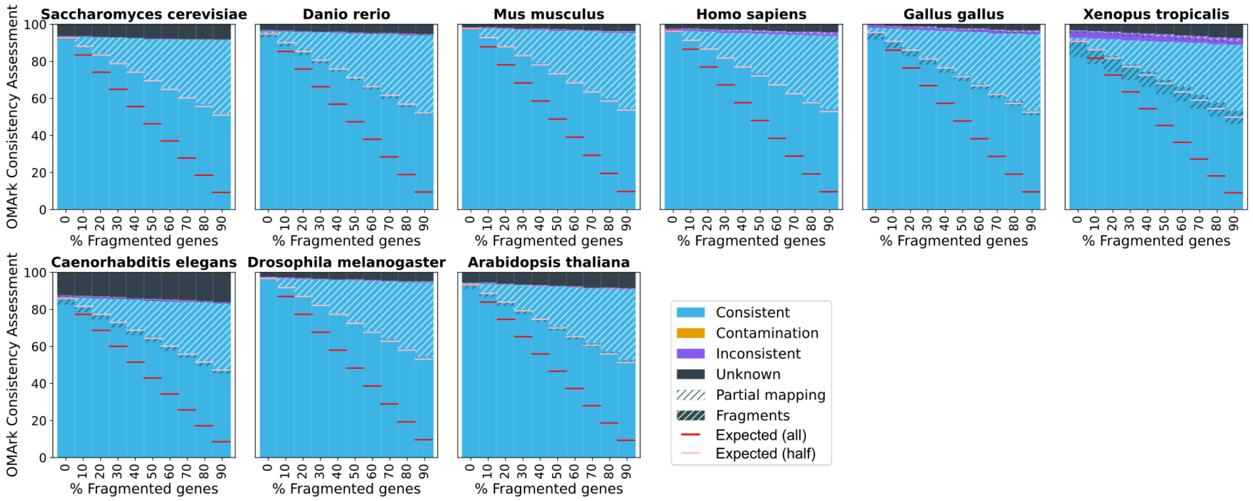

Supplementary Figure 7. **OMArk results for fragmented sequences simulation on "Model" proteomes**. Squares represent OMArk statistics for consistency assessment using different proportions of fragmented proteins in the source proteome, as a proportion of its original size. The species name of the source proteome is indicated on top of each square. The red lines show the expected proportion of taxonomically and structurally consistent genes in the source proteomes of the dataset. The pink lines show the expected proportion of taxonomically and structurally consistent genes if half of the fragments are detected. The proportion of taxonomically and structurally consistent genes (non-hashed blue) stays close to what would be expected considering detection of half the fragments.

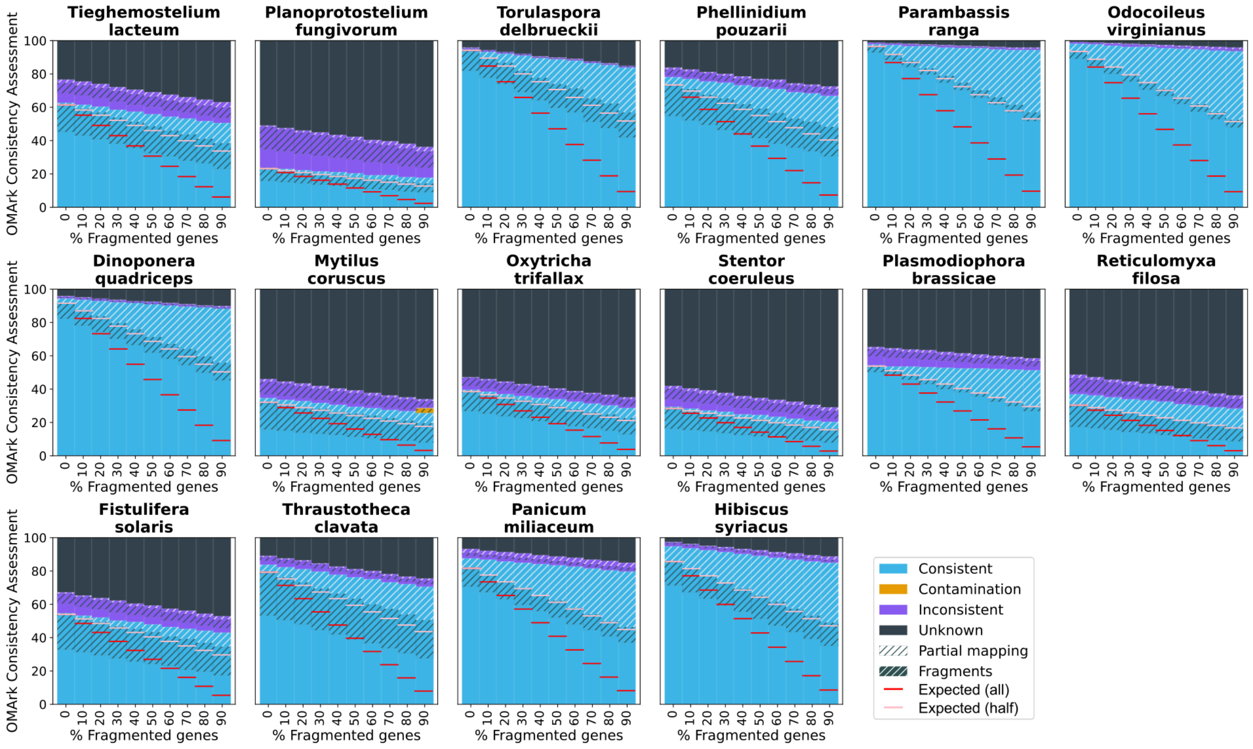

Supplementary Figure 8. **OMArk results for fragmented sequences simulation on "Representative" proteomes**. Squares represent OMArk statistics for consistency assessment using different proportions of fragmented proteins in the source proteome, as a proportion of its original size. The species name of the source proteome is indicated on top of each square. The red lines show the expected proportion of taxonomically and structurally consistent genes in the source proteomes of the dataset. The pink lines show the expected proportion of taxonomically and structurally consistent genes if half of the fragments are detected. The proportion of taxonomically and structurally consistent genes (non-hashed blue) stays close to what would be expected considering detection of half the fragments.

Fused sequences simulation

For each proteome, we artificially fused proteins in the source proteomes. We selected a random pair of sequences, removed less than 20% of the sequences either on the N-terminal or C-terminal end, and appended the trimmed sequences together. The fused protein was added to the proteome while the original ones were removed. We did this from 10% to 90% of the proteome by increments of 10%. All results are shown in Supplementary Figures 9 ("Model" dataset) and 10 ("Representative" dataset).

In both datasets, the fusion of sequences led to a reduction in proportion of Unknown proteins, likely resulting from merging one sequence with no known homologs to one with known homologs for which there is enough signal for placement. The fusion of sequences also leads to an increase in proteins with partial mapping. This is expected as these proteins should be placed into one of the HOGs containing one of the fused sequences, but with only part of the sequence participating in the placement. The red line in the figure shows the expected proportion of taxonomically and structurally Consistent proteins if the fused proteins were placed in another category. The difference to the expectation is -17.6% on average for Model species and -13% on average for Representative species.

This simulation shows that OMArk can somewhat detect errors due to fusion of two distinct protein-coding genes, which could be the result of misannotation of gene models. However, it is

not highly sensitive, likely because OMArk uses only the distance between the first and the last k-mer in common to discriminate partial mapping. Because of this, one randomly occurring *k-mers* in common with the HOG at the end of the non-homologous sequence may result in non-counting as partial mapping and only as consistent.

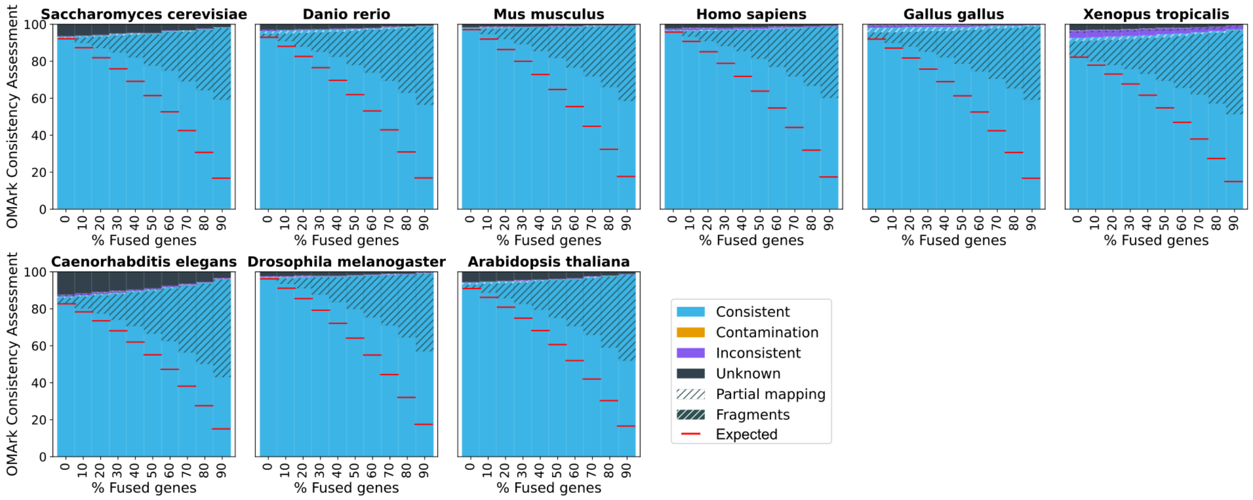

Supplementary Figure 9. **OMArk results for fused sequences simulation on “Model” proteomes**. Squares represent OMArk statistics for consistency assessment using different proportions of fragmented proteins in the source proteome, as a proportion of its original size. The species name of the source proteome is indicated on top of each square. The red lines show the expected proportion of taxonomically and structurally consistent genes in the source proteomes of the dataset. The proportion of taxonomically and structurally consistent genes (non-hashed blue) decreases with more fused proteins but is underestimated compared to the expectation.

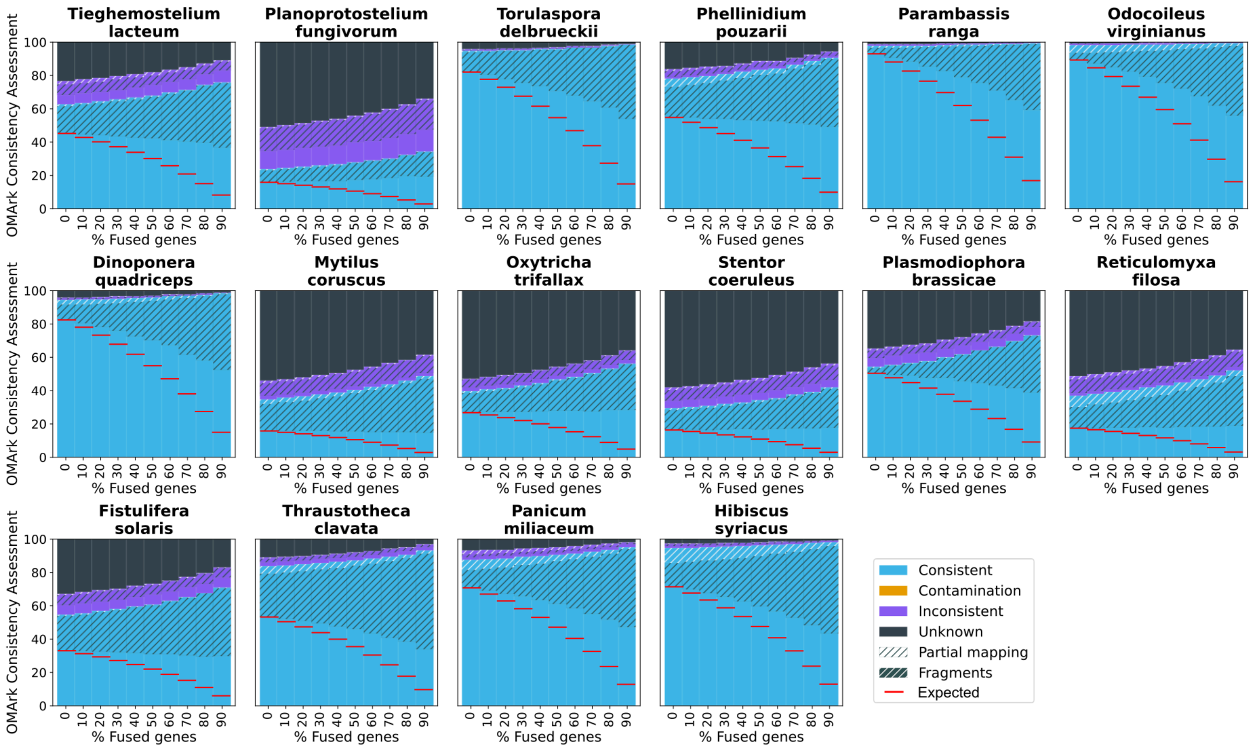

Supplementary Figure 10. **OMArk results for fused sequences simulation on “representative” proteomes**. Squares represent OMArk statistics for consistency assessment using different proportions of fragmented proteins in the source proteome, as a proportion of its original size. The species name of the source proteome is indicated on top of each square. The red lines show the expected proportion of taxonomically and structurally consistent genes in the source proteomes of the dataset. The proportion of taxonomically and structurally consistent genes (non-hashed blue) decreases with more fused proteins but is underestimated compared to the expectation.

Contamination simulation

We simulated different degrees of contamination with exogenous sequences by introducing, independently, proteins from a selection of species in each proteome of the Model and Representative datasets. The choice of the contaminant species was based on their perceived likeliness to contaminate sequence data: human, yeast, a diversity of bacteria often detected as contaminants, and unicellular eukaryotes were added as contaminants. We introduced an increasing number of proteins: 10, 20, 50, 100, 200, 500 and 1000. We then assessed whether the correct species or a taxonomically adjacent species was detected as the contaminant (Supplementary Table 3).

The effectiveness of contamination detection, expectedly, increases with the number of introduced proteins. Even with as few as 10 introduced proteins, contamination from fungi or bacteria is detectable. This is equivalent to roughly 10 kbp for bacterial DNA and 25 kbp for fungal DNA. For all species, barring two exceptions, contamination becomes noticeable at 50 proteins. This represents between about 100 kbp - 175 kbp DNA for unicellular eukaryotes and roughly 7.5 Mbp for human DNA. Detection of contamination appeared to be mainly dependent on the contaminant species and its taxonomic origin. Contamination from bacteria was reliably detected (>50% of the samples) with the introduction of 20 contaminant proteins, equating to ~20 kbp bacterial DNA. For fungi, 50 contaminant proteins were needed (~125 kbp fungal DNA). For other species, the range varied, typically needing between 50 and 200 introduced contaminant proteins. This equates to a maximum of ~700 kbp DNA for unicellular eukarotes and ~30 Mbp DNA for humans.

With our method, contamination is harder to detect between relatively close species. For example, contamination from the fungi *Torulaspora delbrueckii* in *Saccharomyces cerevisia*e proteome could not be detected, even at the highest degree of contamination. Similarly, contamination from *Homo sapiens* in other Vertebrates could not be detected unless the number of introduced proteins was high (1000 proteins; ~150 Mbp human DNA). Even 1000 proteins was never detectable as human contamination for *Mus musculus* and *Xenopus tropicalis*; however it was detected in *Odocoileus virginianus texanus*.

The importance of clade sampling for accurate contamination detection is well illustrated by two Eukaryotic species from lowly sampled lineages in the OMA database: the amoeba *Planoprotostelium fungivorum* and the alveolata *Stentor coeruleus*. Contamination from either of the species could not be detected until we introduced 1000 contaminant proteins. This is likely because the low sampling of the clade in the OMA database does not allow for many specific placements.

Conversely, when the same species were used as the source genome, strong contamination often led OMArk to mistake the contaminant as the most likely main lineage for the proteome. Nevertheless, the results we obtained show that, for contaminant species from well-sampled clades and especially if the contaminant species is distant from the contaminated one, our software can detect even small traces of contamination in the dataset.

Results on UniProt Reference Proteomes

We demonstrate the utility of OMArk on real data by quantifying the quality of 1805 publicly available UniProt Reference Proteomes [6] for eukaryotic species.

Global results

Figure 2 of the main manuscript (reproduced below) summarizes the results of quality assessment over the whole dataset. At first glance, it appears that the overall completeness of the Reference Proteome data is high, with 1552 (86%) proteomes being detected with more than 80% completeness and 46 proteomes (2.5%) missing more than 50% conserved genes. The results of the consistency assessment are more contrasted. Only 1083 proteomes (60.0%) have at least 80% of their proteins categorized as taxonomically Consistent, and 486 proteomes (27%) are more than 80% taxonomically *and* structurally Consistent (excluding Partial mapping and Fragment). Surprisingly, 378 proteomes (20.9%) have less than half their genes that are Consistent both taxonomically and structurally (neither Partial mapping or Fragment).

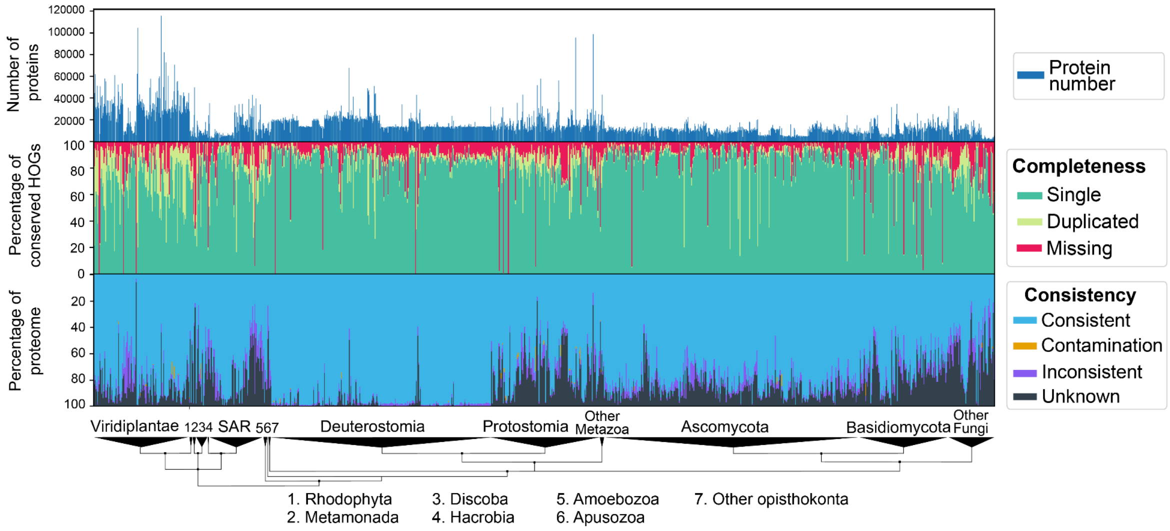

Figure 2 (from main text). OMArk results on 1805 Eukaryotic proteomes from UniProt. Middle - Completeness assessment: Proportion of conserved genes found (green) with a breakdown between single copy (light green) and duplicated (dark green) and missing conserved HOGs (red), in all proteomes ranked by proportion of found genes. Bottom - Whole proteome characterization: Proportion of accurately mapped proteins ("Consistent"; blue), incorrectly placed proteins ("Inconsistent"; purple), contaminant proteins (orange), and proteins with no mapping ("Unknown"; black). Genomes are ranked by taxonomy, with major eukaryotic taxa shown on a taxonomic tree at the bottom.

In terms of taxonomic specificities, a few particularities stand out. Deuterostomes have the highest proportion of taxonomically Consistent genes, however, they do not stand out in terms of completeness. A subset of the plants in the dataset has a high proportion of duplication, as expected in species often subject to whole genome duplication.

We investigated if there is a trend of lower quality for annotations for older assemblies (Supplementary Figure 11). The expectation would be an increase of quality over time, as sequencing technologies improve. Surprisingly, the distributions of OMArk's key quality assessments do not show a clear improvement over time. In fact, they fluctuate over time, and the proportion of Unknown genes even increased over the last part of the 2010 decade. These surprising results do not allow us to conclude there is not an upward trend in quality over time because other factors must be considered. First, the older assemblies in this dataset are only species for which no new assemblies were made available since their first submission; species with lower quality assemblies and annotations may have been resequenced since then. Second, OMArk statistics are influenced by taxonomy, and there is likely a taxonomic bias affecting when

certain species are sequenced. For example, 2020 appears to be a year with higher gene set consistency, but lower completeness and more fragmented gene sets. This anomaly can by partly attributed the influx of bird proteomes from a singular source that year, which had a high prevalence of fragmented proteomes (for further details, see the section "Analysis of avian proteomes").

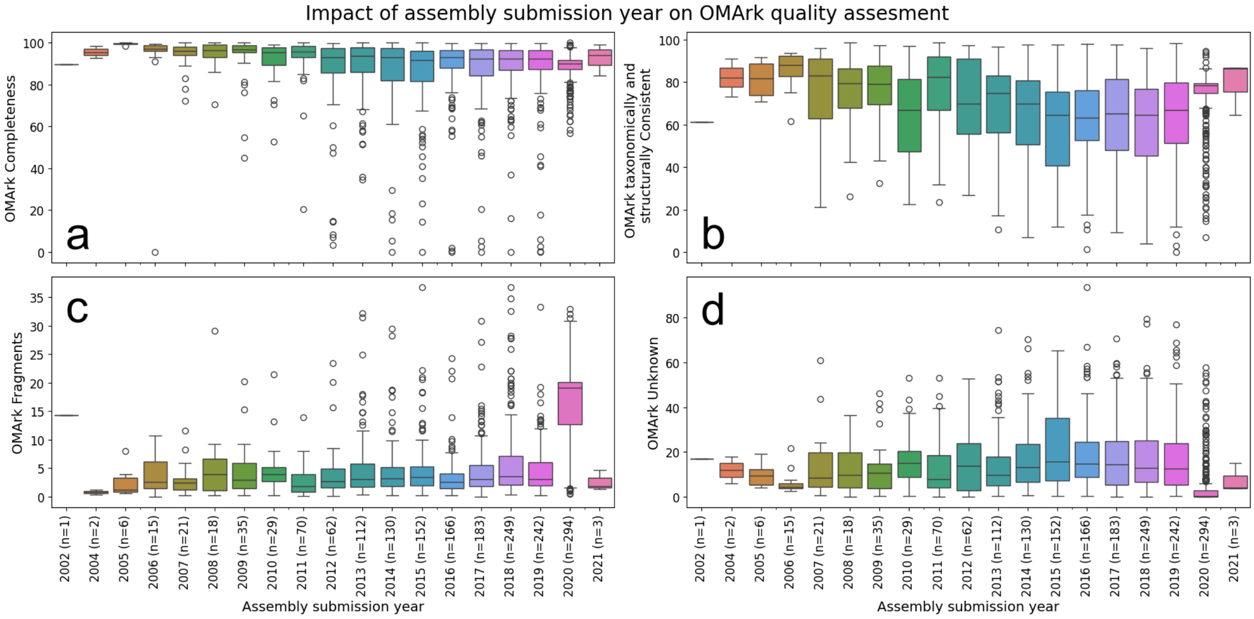

Supplementary Figure 11. Distribution of OMArk results according to the year of submission of the corresponding assembly. Lower bound of boxes represents the first quartile, the center line the median and upper bounds of the box the third quartile of the value distribution. The whiskers extend to the minimal and maximal values of the distribution or to 1.5 the interquartile range beyond the first and third quartile, depending which is the closest. In case it extends to 1.5 the interquartile range, all values beyond it are shown as single points. The number of proteomes for each year is indicated. **a.** Total completeness (Single-copy plus duplicated). **b.** Proportion of the proteome that is taxonomically and structurally consistent. **c.** Proportion of the proteome that is marked as fragmented. **d.** Proportion of the proteome marked as Unknown. Average quality of proteomes do not appear to increase for more recent assemblies.

We used OMArk's results on these 1805 proteomes to assess the accuracy of species identification. The taxon identified by our method corresponded exactly to the most specific taxon containing the species and represented in the OMA database for 849 (47%) proteomes. In scenarios where the OMA database contains close relatives of the query species, OMArk might identify a closely related species or a sister clade instead of the exact one. However, when we considered the ancestral lineage thatOMArk uses post species identification, the choice of ancestral lineage based on species identification was accurate for 1592 proteomes (88.2%). Finally, the majority of the remaining 213 species misidentifications were identified as an ancestral clade of the reference one (165; 9.1% of the total). Selecting such clades for the quality assessment step would lead to less specific but still accurate results.

Thus, in total, the automatic species identification of OMArk was able to select a satisfactory clade in 97.3% of the cases.

Contamination detection and validation

In our analysis of the whole UniProt Reference Proteome dataset, OMArk detected contamination in 79 proteomes. Some of these have contamination from multiple species, leading to a total of 124 detected contamination events. The breakdown of detected

contaminants indicates that the majority were from bacterial species, accounting for 112 of the 124 events (Supplementary Table 4).

To further validate these findings, we checked the time of submission of the assembly associated with each proteome identified as contaminated. Our goal was to determine if older assemblies, which might have faced challenges in contamination checks due to incomplete sequence databases, were more susceptible to contamination. This didn't appear to be the case in our data, with a proportion of contaminated proteomes between 2.5 to 10% for most years spanning 2009 to 2020 (Figure 12).

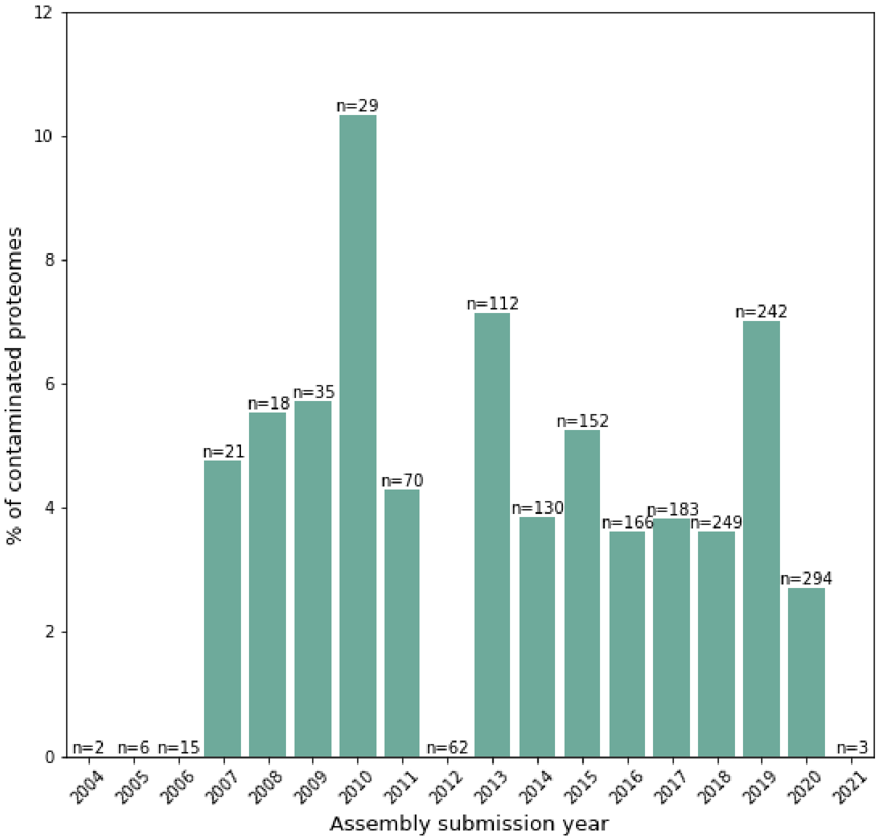

Supplementary Figure 12. Proportion of contaminated proteomes by year of assembly submission. Proportion (as percent of total proteome) which have contamination as detected by OMArk. Each bar represents a year, with the number of proteomes with an assembly submitted this year written on top. The year 2004 is excluded as there is only one proteome this year and it is contaminated, which leads to an uninformative 100%.

In order to validate the contamination calls made by OMArk, we performed two supplementary analyses: one based on a BLAST search of a sample of the contaminant sequences in the non-redundant database and a second based on BlobToolkits results on the assembly corresponding to the proteome of interest.

BLAST cross-validation

For each contamination event detected by OMArk, we selected the first 10 sequences reported as contaminants and performed a search against the non-redundant database. Then for each result, we selected the best hit that was not from the query species itself and asserted whether the target species for this hit was closer to the query species or from the OMArk-determined contaminant species. Results for all of the BLAST searches were reported as closer to the

potential contaminants for 80 events. For 26 other events, more than 80% of the searches were closer to the contaminant.

For those 18 samples with less than 80% successful hits, we manually evaluated the BLAST output to assess a possible contamination. Based on the distribution of best hits, we could confirm at least some of the sequences were likely exogenous and closest to the reported contaminant for an additional 11 OMArk-detected contamination events.

We could not confirm whether the 7 others were actual cases of contamination and labeled them as false positives, for a true positive rate of 94.3% overall. One of the false positives (contamination from *Picea glauca* in the fungi *Mytilinidion resinicola*) originates from contamination in our reference database.

BlobToolkit cross-validation

For each detected contamination event, we obtained the contaminated proteome's identifier for the corresponding assembly from UniProt and used this to query the BlobToolkit webserver. Results from BlobToolkit were available for 89 of our detected contamination events. Of those, 73 were also detected as contaminants in the assembly by Blobtoolkit, corresponding to a true positive rate of 82%. Of the 16 contamination events not detected as such by BlobToolkit, 2 of them were also marked as false positives in our BLAST analyses. However, 13 that were not confirmed by BlobToolkit could be confirmed as contamination by BLAST. In the BLAST results, most contaminant sequences detected by OMArk had a high sequence similarity with the identified contaminant, suggesting they are *bona fide* contamination.

In conclusion, from both the BLAST and BlobToolkit analyses, we could validate 117 of the contamination events detected, corresponding to contamination in 73 species. This corresponds to a true positive rate of 94% on the Reference Proteome data. Overall, OMArk provides a viable indicator of potential contamination events, but we recommend exercising caution and performing a more detailed analysis using complementary tools when using the contaminated proteomes.

Comparison with BUSCO on UniProt Reference Proteomes

We compared OMark completeness to BUSCO's assessment (Supplementary Figure 13a). Here, we define completeness as the percentage of conserved ancestral genes in either BUSCO or OMArk which are found in the query proteome. Note that this includes duplicated and fragmented genes. The results show a high correlation between completeness scores (Pearson: 0.86, p-value: 0) between the results but with divergence for a few proteomes.

In order to assess the difference between OMArk and BUSCO, we classified the 1805 UniProt Eukaryotic proteomes by comparing their completeness scores. Proteomes with a score difference of ≤ 5% were considered to have similar BUSCO and OMArk completeness, encompassing 57.1% of the sample. The rest were split between BUSCO showing higher completeness (18.8%) and OMArk doing so (24.1%). The breakdown of categories is shown in Supplementary Figure 13a.

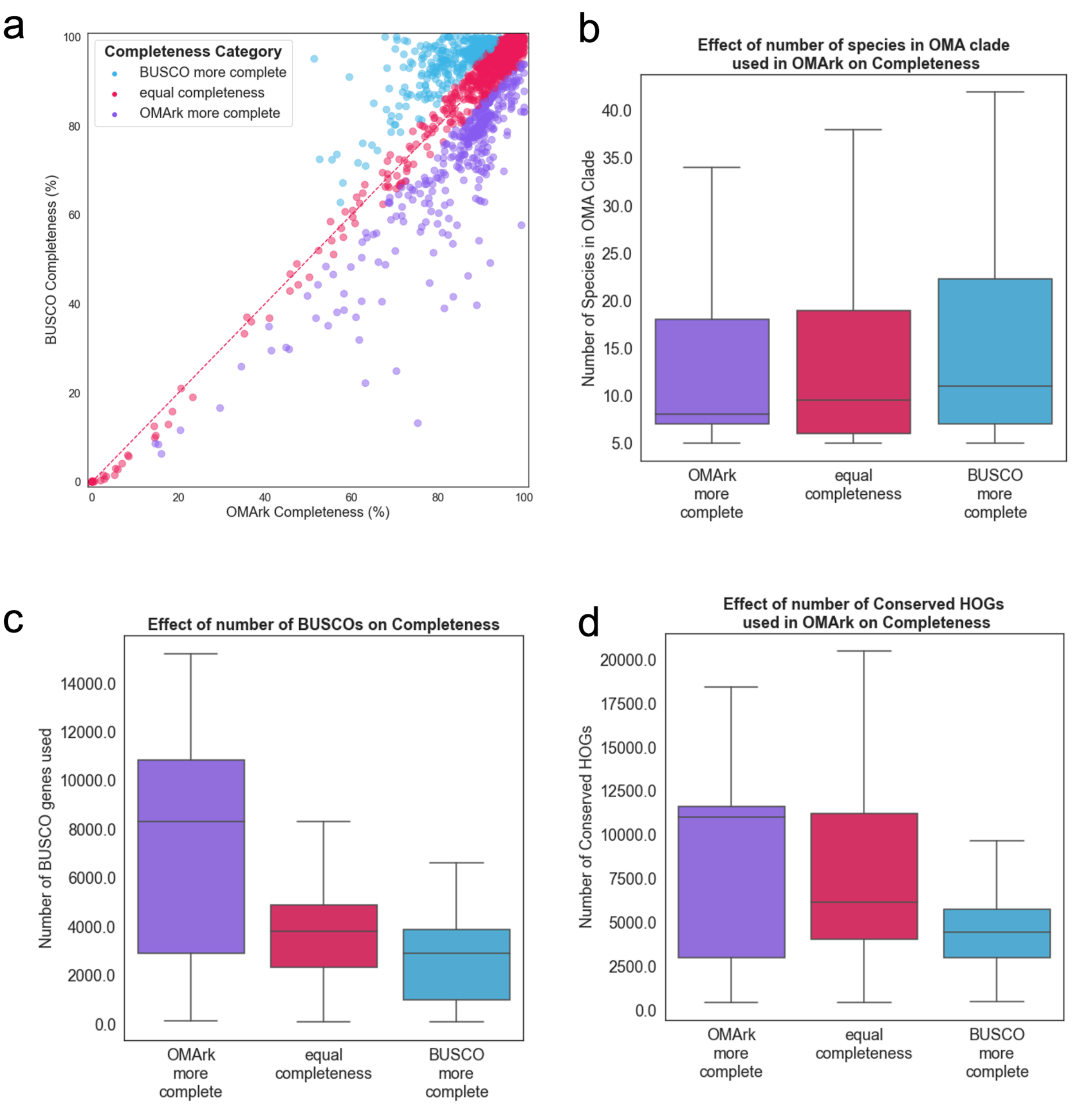

Supplementary Figure 13. Comparative analysis of OMArk and BUSCO completeness. A) Scatterplot depicting the relationship between OMArk and BUSCO completeness levels. Here completeness includes both fragments and duplicated genes (all those not missing). Points represent individual proteomes and are colored based on which method indicated greater completeness: BUSCO (blue), OMArk (red), or equal (purple). The dashed line represents the line of equality. B) Box plots illustrating the effect of the number of species in the clade in OMA for which OMArk used for assessment. C) Box plots illustrating the effect of the number of BUSCO single-copy genes used for BUSCO assessment. D) Box plots illustrating the effect of the number of Conserved HOGs (i.e. ancestral repertoire) used for OMArk assessment. For boxplots, the lower bound of boxes represents the first quartile, the center line the median and upper bounds of the box the third quartile of the value distribution. The whiskers extend to the minimal and maximal values of the distribution or to 1.5 the interquartile range beyond the first and third quartile, depending which is the closest. Box plot categories are “OMArk more complete” n=434 proteomes where OMArk Completeness is higher than BUSCO Completeness by 5%. “equal completeness” n=1030 proteomes where OMArk and BUSCO Completeness are within 5%, “BUSCO more complete” n=340 proteomes where BUSCO Completeness is higher than OMArk Completeness by 5%.

	OMArk more complete	equal completeness	BUSCO more complete
N	435	1030	340
OMArk % Duplicated	3.9	4.0	3.0

BUSCO % Duplicated	0.3	0.6	0.4
OMArk % fragment	17.8	2.9	3.0
BUSCO % fragment	5.9	1.4	1.5
OMArk % Missing	10.4	4.5	13.6
BUSCO % Missing	20.8	3.7	3.4
OMArk % Inconsistent	1.5	2.6	8.3
Number of BUSCOs	8338	3817	2898
Number of conserved HOGs (OMArk)	10994	6137	4436
Number of species in OMA clade (OMArk)	8	9.5	11

Supplementary Table 7. Median values of various quality metrics for each Completeness category in the 1805 Eukaryotic proteomes dataset. Proteomes were classified as OMArk more complete if OMArk showed a 5% or higher completeness score than BUSCO. Proteomes were classified as BUSCO more complete if BUSCO showed a 5% or higher completeness.

In proteomes where OMArk was deemed as more complete, there was a higher proportion of fragments as identified by both BUSCO and OMArk. Proteomes with OMArk resulting in higher completeness had a median of 17.8% of the proteomes identified as fragments, in contrast to those where BUSCO was more complete (median 3.0% fragments). Those deemed equally complete had a similar fragment level of 2.9% (Supplementary Table 7). This suggests OMArk is better at identifying fragmented proteins without labeling them as missing.

The difference in BUSCO vs. OMArk completeness is likely affected by differences in the ancestral clade selection. Indeed, when breaking down the completeness scores by lineage (as defined in BUSCO), several lineages display a bias towards being either more complete when analyzed with BUSCO or more complete when analyzed with OMArk (Supplementary Figure 14). For example, the *passeriformes*, *nematoda*, and *certartiodactyla* clades tend to be consistently showing more complete with OMArk, whereas clades like *stamenopiles* and *chlorophyta* consistently were shown as more complete with BUSCO. However, these differences are likely due to the underlying clade used for assessment and consequently, the number of conserved genes used. Proteomes that were deemed more complete by BUSCO tended to use a smaller number of BUSCOs for assessment, whereas proteomes deemed more complete by OMArk tended to use a smaller number of conserved HOGs for the assessment.

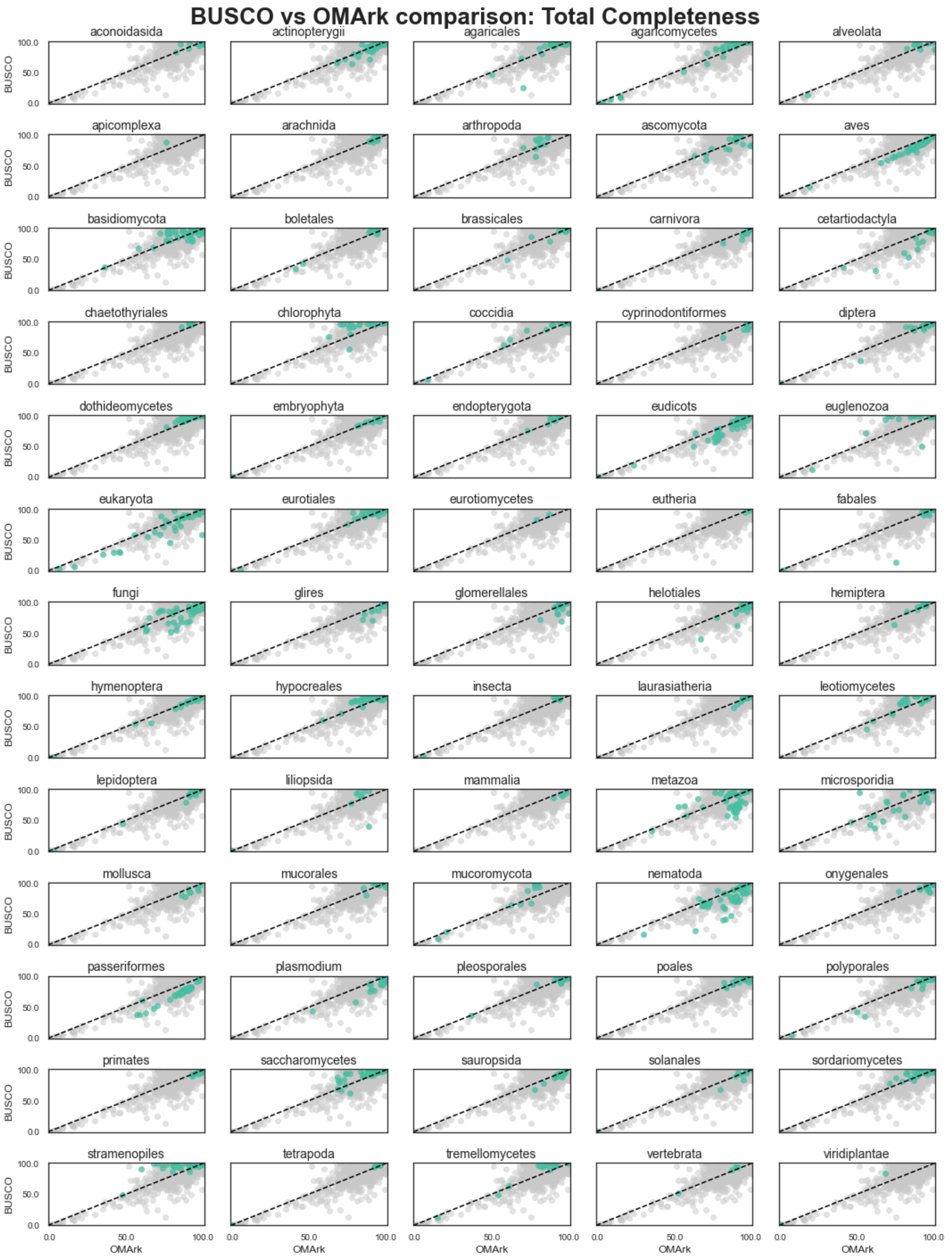

Supplementary Figure 14. Scatterplots of BUSCO vs. OMArk completeness for the 1805 Eukaryotic proteomes, broken down by lineage. The x-axis shows OMArk completeness, and the y-axis shows BUSCO completeness. Both scores include duplicates and fragments. In each plot, the proteomes belonging to that lineage are highlighted in green.

For example, results of both methods using the *Aves* (Supplementary Figure 15) lineage as source are clearly collinear (Pearson: 0.98, p-value: $2e^{-77}$). Another salient example is the Nematoda lineage, where the results are visibly different: The extreme values where the OMArk completeness was much higher than BUSCO completeness appear to be due to a higher

percentage of fragmented genes. In the Stramenopiles example, the extreme values of BUSCO completeness compared to OMArk completeness are likely related to the low number of single-copy BUSCO genes used for assessment..

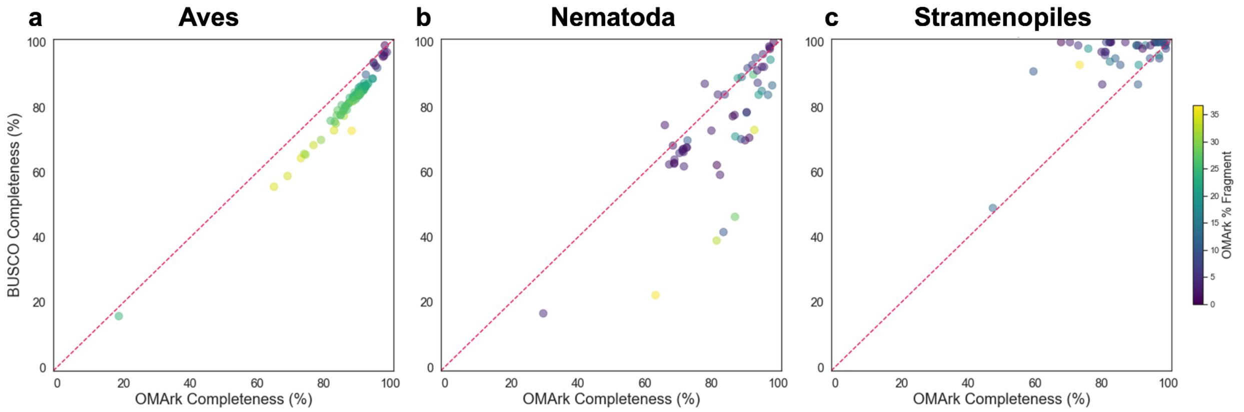

N=112
BUSCO nb = 8338
Conserved HOGs nb = 10994

N=55
BUSCO nb = 3131
Conserved HOGs nb = 5500

N=44
BUSCO nb = 100
Conserved HOGs nb = 5742

Supplementary Figure 15. Comparison of BUSCO and OMArk completeness assessment for example clades. A) **Aves, where t**he completeness assessment is nearly identical between methods. B) Nematoda, where OMArk finds a higher level of completeness. C) Stramenopiles, where BUSCO finds a higher level of completeness, likely due to the low number of BUSCOs used for assessment. N is the number of proteomes for which the BUSCO lineage for this clade was used.

OMArk runtime assessment and comparison with BUSCO

When running the OMArk pipeline on our dataset of 1805 proteome, we evaluated the overall runtime as well as its individual steps (the OMAmer software and OMArk software) through the Slurm scheduling system Job Efficiency Report. We found that the CPU runtime efficiency was mainly dependent on the number of proteins in the analyzed proteomes (Supplementary Figure 16).

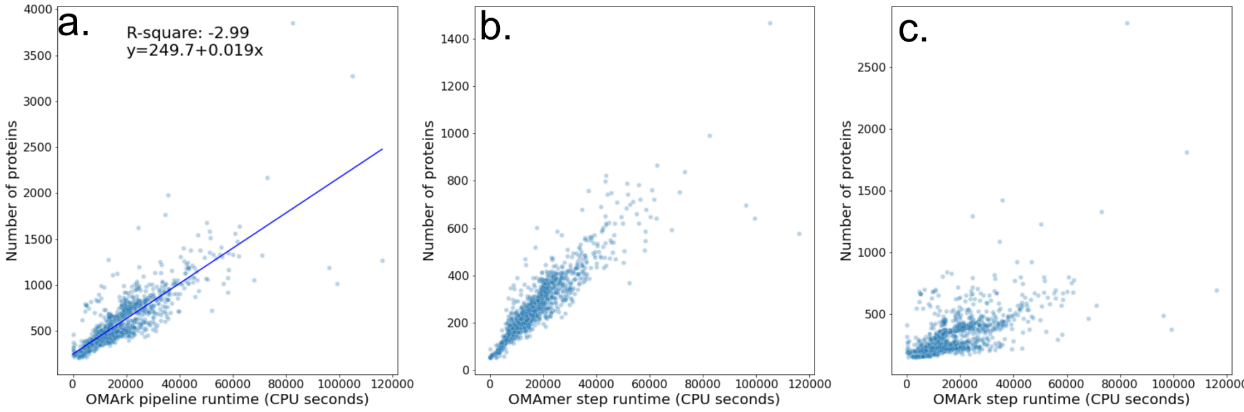

Supplementary Figure 16: CPU runtime of the OMArk pipeline for proteomes with various protein numbers. **a.** CPU runtime of the OMArk pipeline. Linear regression is shown as a line, with its parameters written on top. **b.** CPU runtime of the OMAmer step. **c.** CPU runtime of the OMArk step.

On the whole data, the average runtime for the pipeline was 9.2 minutes per proteome, with 4.15 minutes for OMAmer and 5.05 minutes for OMArk. The lowest runtime was 3.5 minutes for the microsporidian parasite *Enterospora canceri* with 2,147 proteins and the longest took 64 minutes for the *Triticum aestivum* proteome with 82,460 proteins. As mentioned before, the runtime of the whole pipeline increased close to linearly with the number of proteins in the proteome (linear regression R^2: 0.7). The OMAmer part in particular exhibits a strong linear component (linear regression R^2: 0.83), while runtime was not as strongly dominated by proteome size in the case of OMArk (linear regression R^2:0.41). This likely indicates the OMArk runtime is also impacted by factors other than proteome size. Indeed, OMArk operations also include retrieval of conserved HOGs for a particular lineage, as well as known HOGs in this lineage through querying the OMAmer database; before comparing it with the observed HOGs. This likely explains the differences in OMArk runtimes for proteomes with the same number of proteins. Nevertheless, our analysis shows that most OMArk runs can be concluded in less than one hour, and often less than 30 minutes.

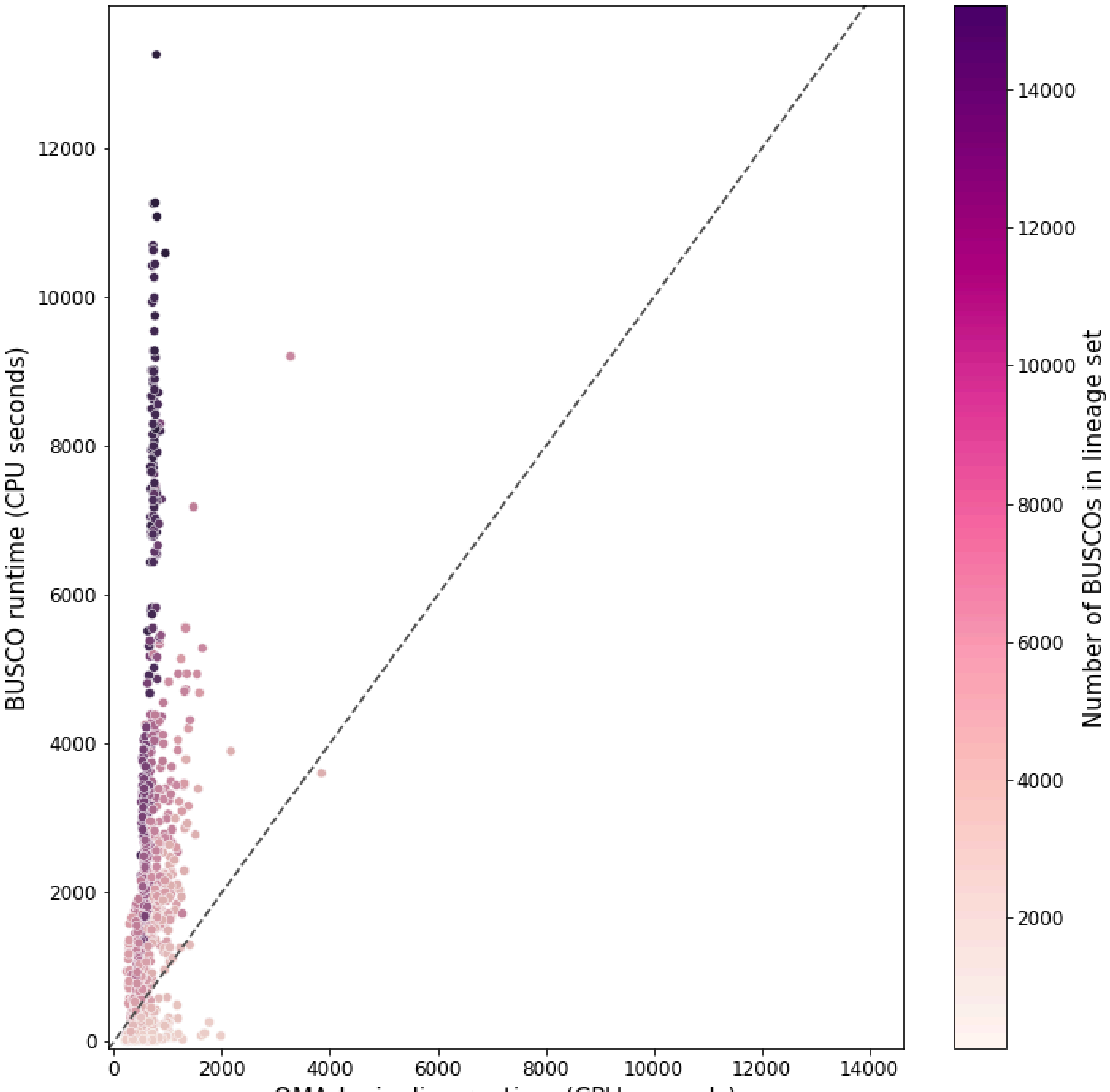

Supplementary Figure 17. CPU runtime comparison of the OMArk pipeline and BUSCO on 1805 proteomes. Runtime in CPU seconds of the OMArk pipeline (x-axis) and BUSCO (y-axis). Each dot corresponds to one of the 1805 UniProt Reference Proteomes

The coloring corresponds to the number of BUSCO genes used for each proteome. BUSCO is faster than OMArk for proteomes for small BUSCO sets but slower for larger BUSCO sets.

We compared OMArk runtimes to that of BUSCO for each proteome in our dataset (assuming an already downloaded lineage dataset)(Supplementary Figure 17). On this dataset, OMArk was faster than BUSCO for more than two-thirds of the dataset (1253 proteomes, 69%) with an average BUSCO runtime of 25.2 minutes per proteome (more than double the 9.2 minutes per proteome from OMArk). The runtimes of BUSCO and OMArk were not correlated, which can be explained by the fact that OMArk's runtime depends mainly on proteome size, while BUSCO's runtime is dominated by the number of proteins in the reference lineage dataset. Thus, BUSCO runtime is often more than one hour for big datasets, while it is well under 10 minutes for proteomes in less sampled and broader clades, making it faster than OMArk on these datasets.

The difference in runtime between BUSCO and OMArk comes mainly from their different way of associating proteins to orthologous groups. BUSCO uses an HMM-based method to search for conserved proteins in a proteome. This is a specific method of association, but can be slow when there are a lot of proteins to look for. On the other hand, OMArk needs all proteins in a proteome to be placed into HOGs, but does it with a faster albeit possibly less specific method. OMArk is therefore not as highly impacted by the number of conserved HOGs it considers.

Case studies of proteomes with outlier OMArk values

Finally, we conducted targeted analyses of proteomes that OMArk identified as having particularly low quality, to confirm it was representative of *bona fide* artifacts in the source data.

The proteomes with the lowest completeness values, according to both OMArk and BUSCO, tend to be proteomes with a low protein count, with a median of 616 proteins for proteomes with <20% completeness (single + duplicated). This is expected and indicates that the completeness value is likely representative of the proteome as a whole. We focus here on a few exceptions: proteomes with low completeness statistics and high protein count such as the white spruce *Picea glauca* (6,155 proteins, OMArk completeness: 0.58%) and the Fungi *Auricularia subglabra* (strain TFB-10046 SS5) (5,290 proteins, OMArk completeness: 14.68%).

For the first example, *Picea glauca,* looking at the proteome characterization statistics gives an additional insight: 93.66% of the proteins are categorized as Unknown. Only 3.74% of proteins from this species are homologous to gene families consistent with its lineage and most of them have divergent gene structures compared to the known members of their gene families and are mostly classified as Partial mapping (1.84%) or Fragmented (0.42%). This indicates that the proteome for this species contains a high proportion of mis-annotated genes, and otherwise not well-defined gene models. Furthermore, the number of proteins in the proteome is uncharacteristic of land plant species and while no other Acrogymnosperm species is present in UniProt for comparison, the Chinese yew *Taxus chinensis* has more than 44,000 protein-coding genes according to the NCBI database. It is thus likely that this proteome is both incomplete and contains mostly erroneous sequences.

The picture is different for *Auricularia subglabra*, which, despite lacking a high proportion of the 'Conserved HOGs' (OMArk completeness: 14.68%), has 49.38% of genes that correspond to expected gene families for its taxonomic division (*Consistent*: 39.19%). This indicates that at least half of the gene count consists of well-annotated genes. The low completeness level suggests that the complete genome is expected to have a much higher count of protein-coding

genes than the 5,290 reported in the proteome. This is confirmed in the literature, where the number of protein-coding genes for species in the genus is around 16,000 [7].

The proteomes with the highest amount of proteins placed into taxons inconsistent with the species' lineage tend to be from taxonomic groups underrepresented in the OMA database. Of the 10 proteomes with the highest "Inconsistent" score, we count 2 from the SAR division, 6 chlorophytes, 1 Rhodophyte, and 1 Apusozoa. In these cases, a high proportion of inconsistently placed genes may be due to uneven sampling of these taxonomic divisions in the OMA database and not only to dubious gene models. For example, one of the species with the highest Inconsistent percentage (45.29%) is *Cafeteria roenbergensis,* a species from the Stramenopiles clade. When performing a BLAST similarity search against the non-redundant NCBI database using sequences from the Inconsistent category, we consistently found high percent identity hits in multiple strains of the same *Cafeteria roenbergensis* species, with subsequent hits belonging to a variety of other species and with much lower identity, indicating a likely distant homology relationship.

High proportions of inconsistently placed proteins can also be due to faulty taxonomic labeling. For example, the *Paulinella micropora* proteome also displays a high proportion (68.12%) of Taxonomically Inconsistent proteins. From the known taxonomy of this species, OMArk used the SAR clade as the ancestral lineage. The *Paulinella micropora* proteome is detected as incomplete (only 6% conserved HOGs), yet most misplaced proteins are a perfect match. These misplacements appear to be due to exogenous proteins: OMArk's automatic species placement attributes the whole proteome to the *Cyanobium gracile* family, a cyanobacteria and, importantly, is not able to detect any other species from the protein placement. Since *Paulinella micropora* is a unicellular eukaryote known to have recently acquired a plastid by primary endosymbiosis [8], we can thus hypothesize that this proteome contains mainly genes derived from the genome of the plastids of this species.

In these two cases, the Inconsistent statistics are not necessarily an indicator of erroneous gene prediction, but indicate either species with no close relatives or inaccurate species labeling. In both cases, most of 'Inconsistent' genes are neither labeled as Fragmented nor Partial hits, as would be expected for erroneously predicted genes (like in our simulations). One can thus use this additional information to discriminate a situation where the Inconsistent proportion is indicative of OMArk misattributions rather than faulty gene predictions.

To exemplify this, the proteome of the chlorophyte *Spirometra erinaceieuropaei* also displays a high number of Inconsistent placements (27.95% of the proteome). In this case, however, a large fraction of it is detected as Partial mapping (18.80%) or Fragmented (3.80%), consistent with erroneous gene models. The proteins categorized this way also tend to be rich in repetitive peptides, which explains why they were placed significantly in a non-homologous HOG.

Analysis of avian proteomes

In the precomputed UniProt Reference Proteomes, most of the 234 avian proteomes are characterized by an unusually high proportion of Fragmented genes (average taxonomically Consistent Fragment proportion: 18.3%, standard deviation: 4.83) (Supplementary Figure 18). This proportion of fragments is not uniform across species (0.49% minimum to 31.8% maximum of taxonomically Consistent Fragments). They vary depending on the source of the external annotation data used by the UniProt Reference proteome (Supplementary Figure 19).

Proteomes from Ensembl (n=9, average Consistent Fragment proportion: 2.11%, standard deviation: 2.25) [9] and NCBI RefSeq (n=4, average Taxonomically Consistent Fragment proportion: 2.2%, standard deviation: 1.88) [10] have much fewer Fragments than proteomes uploaded by the Bird 10K consortium (B10K) [11], which represent most of the dataset (n=212, average taxonomically Consistent Fragment proportion: 19%, standard deviation: 2.4).

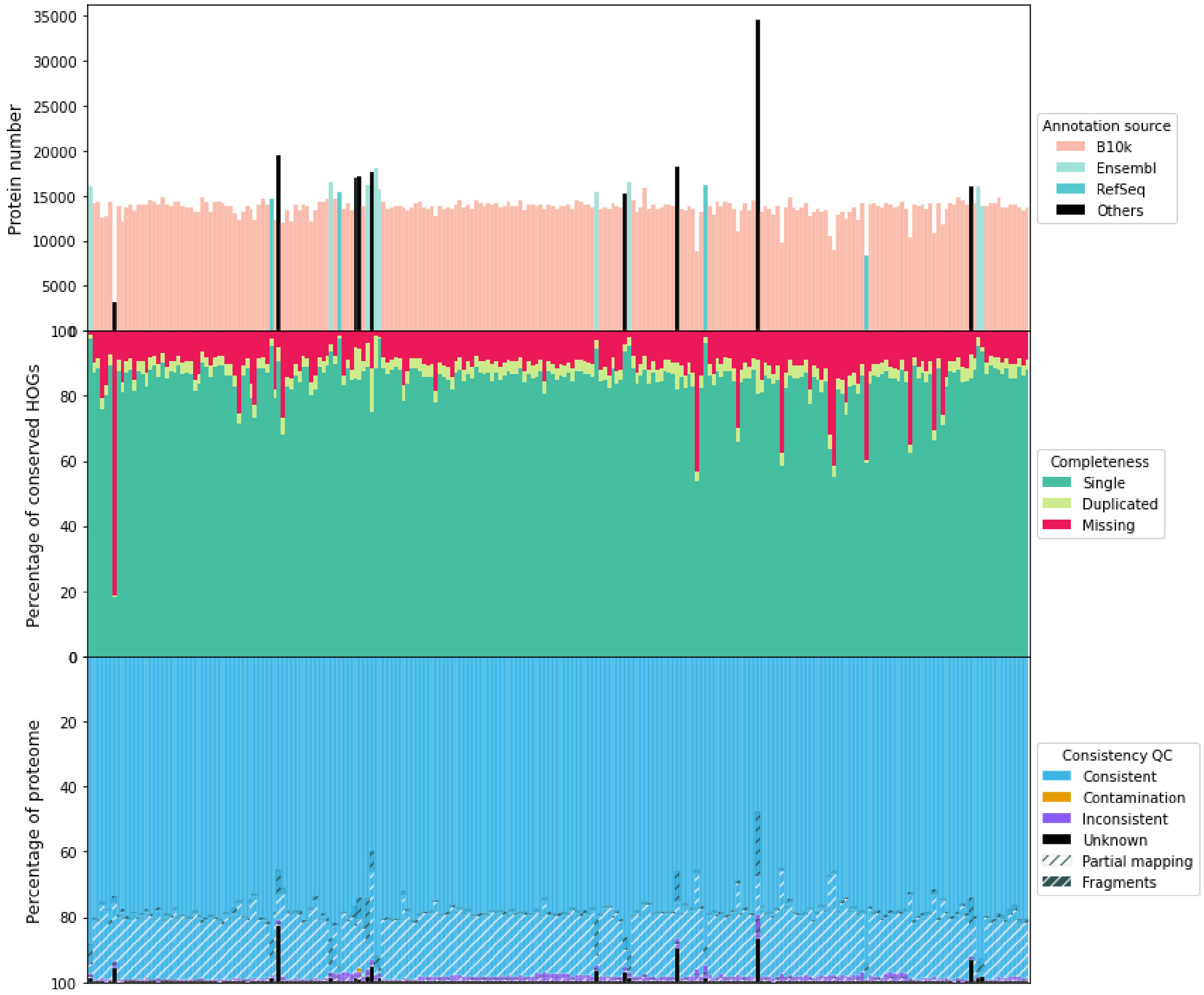

Supplementary Fig 18. OMArk results for Avian proteomes. Each column corresponds to an avian species in the UniProt Reference proteome, ordered taxonomically according to the NCBI taxonomy. The top subplot represents the number of protein-coding genes in each proteome, colored by annotation source. The middle subplot represents completeness, as the proportion of conserved genes present or missing. The lower subplot represents the consistency assessment.

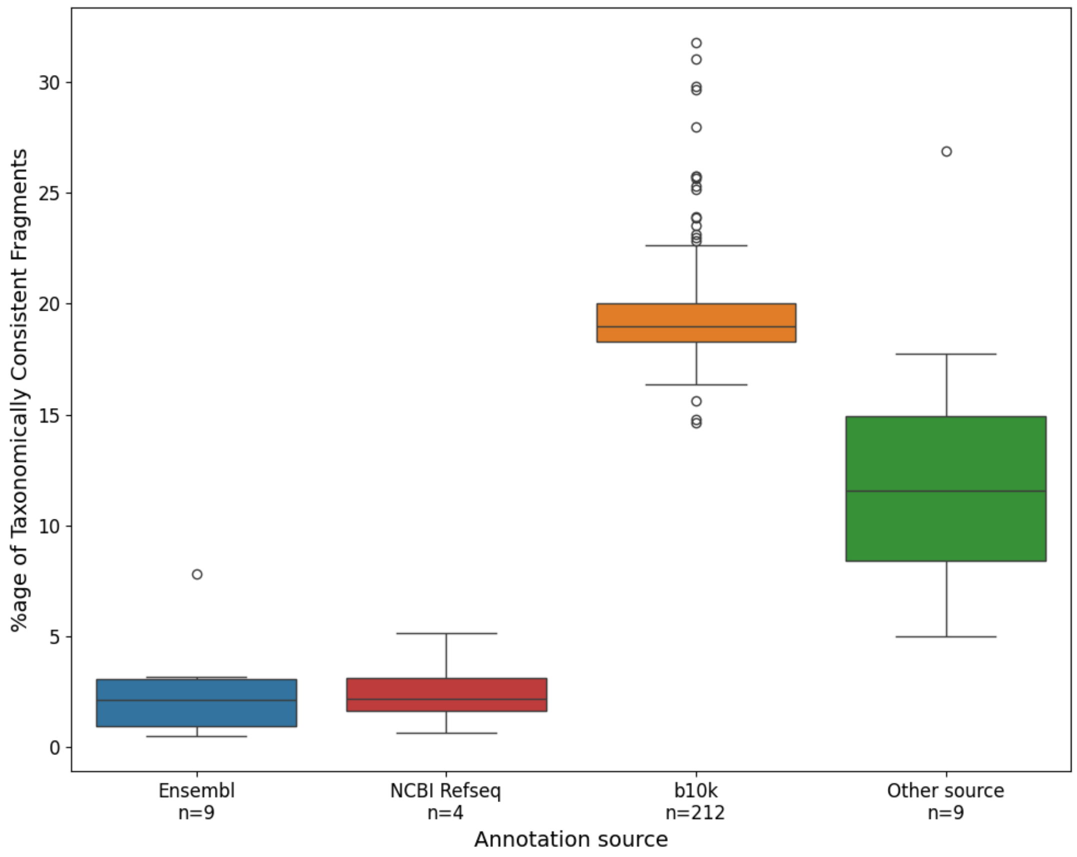

Supplementary Fig 19. Relation between fragment proportion and annotation source. Each category corresponds to a source of annotation.Lower bound of boxes represents the first quartile, the center line the median and upper bounds of the box the third quartile of the value distribution. The whiskers extend to the minimal and maximal values of the distribution or to 1.5 the interquartile range beyond the first and third quartile, depending which is the closest. In case it extends to 1.5 the interquartile range, all values beyond it are shown as single points. Proteomes with B10K annotation are characterized by high amounts of fragments while proteomes from Ensembl and RefSeq have less.

The proportion of fragments being similarly high from all proteomes from the B10K consortium suggests that it is due to systematic bias in the data generation. In order to confirm this, we measured the overlap between the Fragmented gene set between each bird species by using the set of root HOGs into which these fragmented genes were placed by OMAmer. All pairwise overlap measures (calculated as the cardinality of the intersection of the sets divided by the cardinality of the smallest set) are represented in Supplementary Figure 20. The overlap between the B10K proteome fragment set is higher (average: 0.75) than with proteomes from different sources (average: 0.34). Hierarchical clustering based on overlap confirms that proteomes from the B10K annotation dataset form homogeneous clusters in terms of shared fragments.

The clustering reveals additional substratification of the dataset in terms of fragment overlap, suggesting another source-based systematic bias in part of the dataset, but we could not identify an obvious cause. Still, these results show high similarity in terms of fragmented genes for proteomes originating from this project. It could either be related to bias in the sequencing, assembly, or annotation process to generate these data. Because fragmented proteins from this dataset tend to fall within the same gene families, it is most likely the latter.

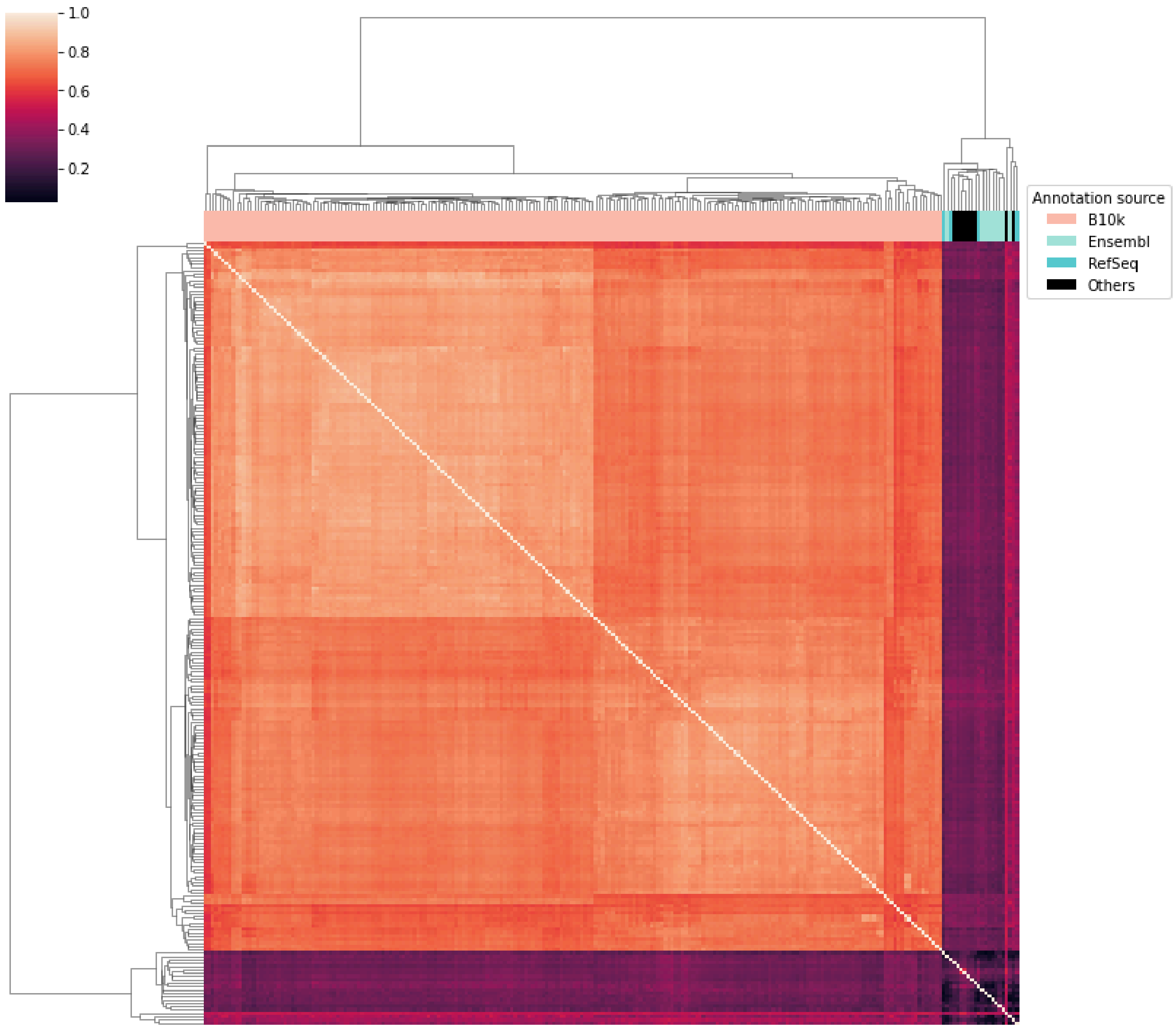

Supplementary Figure 20. Clustered heatmap of overlap of sets of fragmented gene families between genomes. Pairwise overlap is measured as the size of the set intersection over the size of the smallest set and is represented by the color in the heatmap, as coded by the color scale. All proteomes from B10K cluster together and away from other sources of annotation, with high similarity between fragmented genes.

The annotation protocol for the B10K annotation includes, among other sources of evidence, homologous annotation from the Ensembl 85 [9] version of the zebra finch (*Taeniopygia guttata*) proteome. This proteome was annotated on the TaeGut3.2.4 version of the genome assembly. OMArk assessment for this version of the zebra finch proteome reports 20.40% lineage-consistent fragments, much higher than for the current assembly for the same species included in our dataset (0.49%). This is consistent with the improvements of gene models in zebra finch with the latest assembly noted in [12]. We hypothesize that part of the cause for the high proportion of fragments in the B10K annotation is due to error propagation from this fragmented proteome. Accordingly, comparing the overlap between the set of gene families with fragments from the previous version of the zebra finch annotation (Supplementary Figure 21), we show that proteomes from the B10K annotation share, on average, more than half of their fragments with it (average percentage of fragments shared: 76.63%), nearly double the proportion in proteomes from any other sources (average percentage of fragments shared: 40.84%).

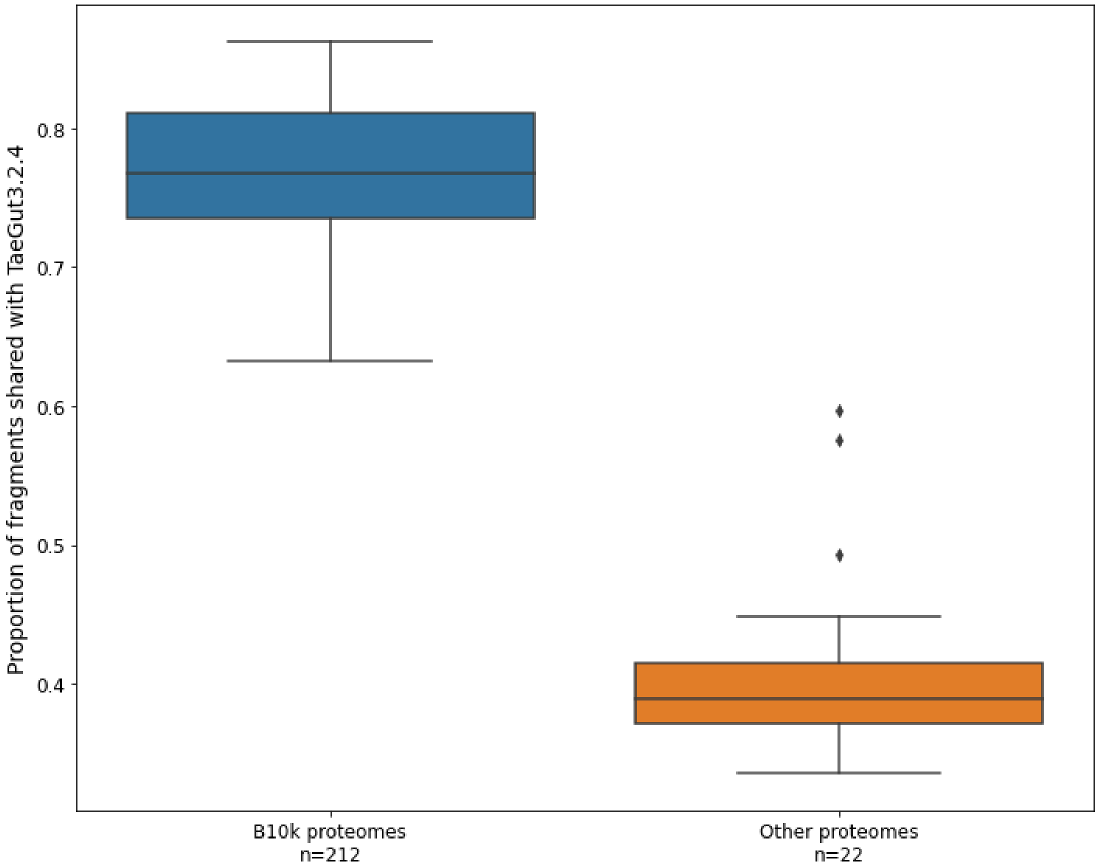

Supplementary Figure 21. B10K annotated proteomes share many fragments with the previous version of the zebra finch proteome. Proportion of fragmented genes that are also fragmented in the proteome for TaetGut3.2.4 assembly. Lower bound of boxes represents the first quartile, the center line the median and upper bounds of the box the third quartile of the value distribution. The whiskers extend to the minimal and maximal values of the distribution or to 1.5 the interquartile range beyond the first and third quartile, depending which is the closest. In case it extends to 1.5 the interquartile range, all values beyond it are shown as single points. All proteomes from B10K have more fragments in common with the zebra finch proteomes than any other proteome in the dataset.

Taken together, these results provide evidence that the high proportion of fragments in bird species in the UniProt Reference Proteome result, at least in part, from error propagation from the reference proteomes used to perform gene annotation.

Comparison of proteomes from closely related species

Similar to BUSCO or other methods based on reference gene sets, OMArk is best used for comparing the proteomes of closely related species. In this context, it can be used to identify the best quality proteomes within a certain taxonomic division. We exemplify this by comparing the OMArk assessment of well-studied, model organisms to the closest related species in the UniProt Reference Proteome dataset.

Human and *Hominidae* proteomes

Proteomes from the *Hominidae* taxa are homogeneous in terms of OMArk statistics (Supplementary Figure 22), with high completeness and a high proportion of genes found to be

Consistent with their taxonomic division, with few Fragments and Partial mapping. The human proteome has a low proportion of missing genes (1.99%), although the chimpanzee has even less (*Pan troglodytes*, 1.37%). The closely related *Pan paniscus* appears to be missing more genes than both (3.79%).

In terms of consistency assessment, *Pongo abelii* has a higher proportion of divergent gene structure than others (88.33% of taxonomically and structurally Consistent genes, respectively, compared to 95.79% - 96.89% for the rest). In addition, *Pongo abelii* also displays a higher proportion of Missing (7.30%) and Duplicated (9.60%) genes than all the other proteomes, likely indicating a slightly worse assembly or gene annotation.

Finally, we note that surprisingly the Human proteome has a slightly higher proportion of "Unknown" proteins than the chimpanzee, bonobo and gorilla. This seems to correspond to short proteins with high compositional bias, for which homology may be hard to detect, or proteins for which there is dubious evidence (260 occurrences of "Putative" in the header on 452 proteins). This outlines the limitation of OMArk, which can only estimate if there is evidence of homology, and could label novel or fast-evolving genes as Unknown. It is then best to rely on other evidence to decide on the status of these genes.

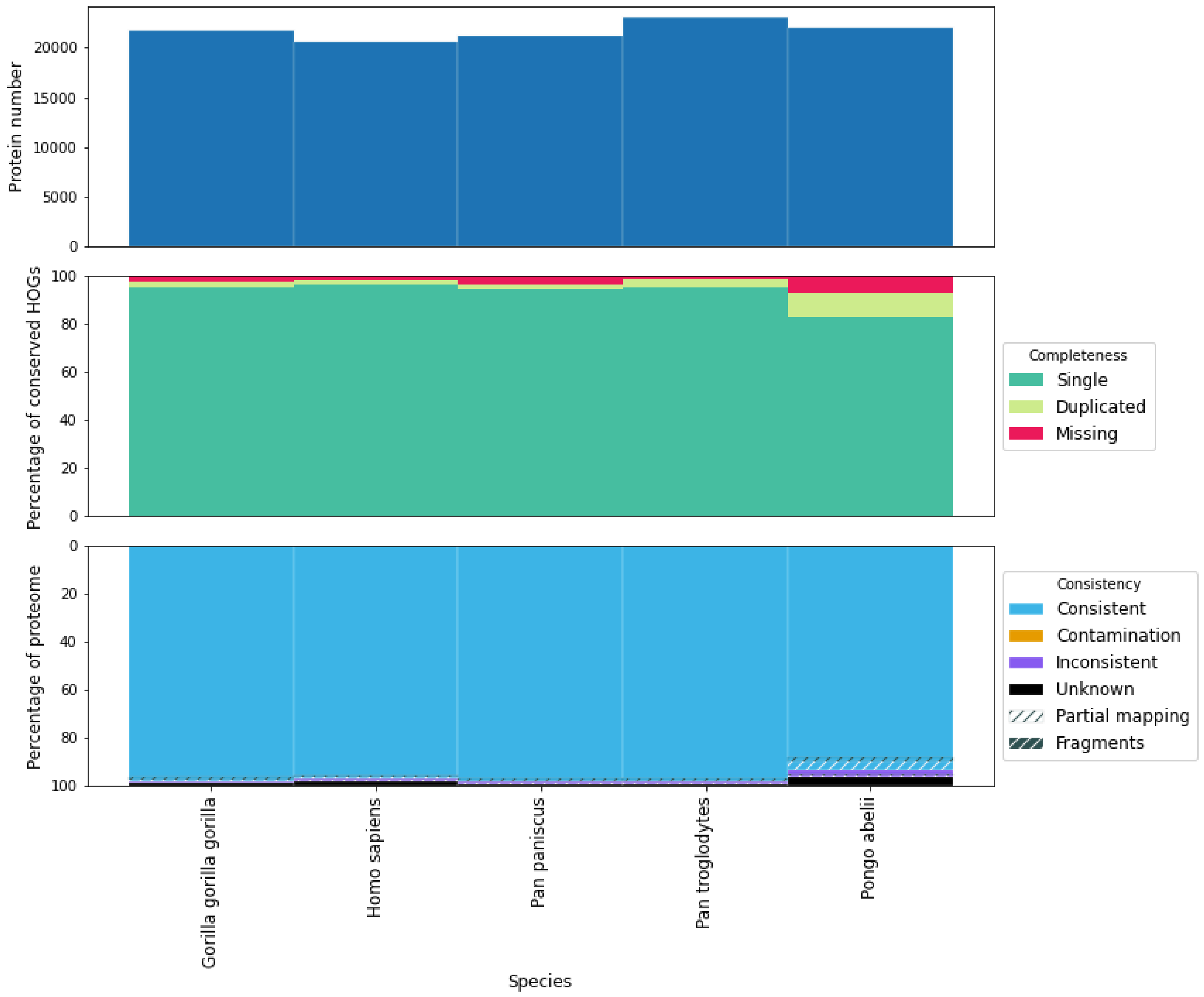

Supplementary Figure 22. **OMArk results for proteomes from the *Hominidae* clade.**

Mouse and *Myomorpha*

Proteomes from the *Myomorpha* clades are noticeably different, both in terms of completeness and gene model quality (Supplementary Figure 23). As expected, the mouse proteome is highly complete (99.42% completeness) and consistent (97.12% of taxonomically and structurally Consistent genes), as is the *Rattus norvegicus* proteome, another well-studied rodent (97.79% completeness, 94.52% taxonomically and structurally Consistent genes). *Mesocricetus auratus* and *Peromyscus maniculatus* have a slightly higher proportion of genes with divergent gene models (90.21% and 91.61% taxonomically and structurally Consistent genes, respectively) and Duplicated genes (6.77 and 4.86%, respectively), indicating the proteome may be less reliable. Finally, *Neotoma lepida* and *Cricetulus griseus* display a comparatively high proportion of Missing (8.84% and 15.03%, respectively) and Duplicated genes (12.01% and 7.63%, respectively), and less than 60% of taxonomically and structurally Consistent gene models. This indicates the gene content is not only incomplete but of lesser quality overall. This is outlined by the number of reported coding genes being higher from the other species by a few thousand while being less complete overall. This result is particularly notable given *Cricetulus griseus* is included in our reference database, showing our method is somewhat robust to circularity (flagging a proteome as high quality because it has an exact counterpart in the reference dataset). Accordingly, one should be cautious when using these in comparative studies.

The number of proteins in this dataset was positively correlated with the number of fragments (Pearson correlation: 0.85), Partial mapping proteins (Pearson correlation:0.79) and Unknown proteins (Pearson correlation: 0.73). Surprisingly, it was also positively correlated with the proportion of Missing proteins (Pearson correlation:0.8), suggesting that genome fragmentation by increasing the number of fragmented gene models overall inflates the protein number despite being less complete.

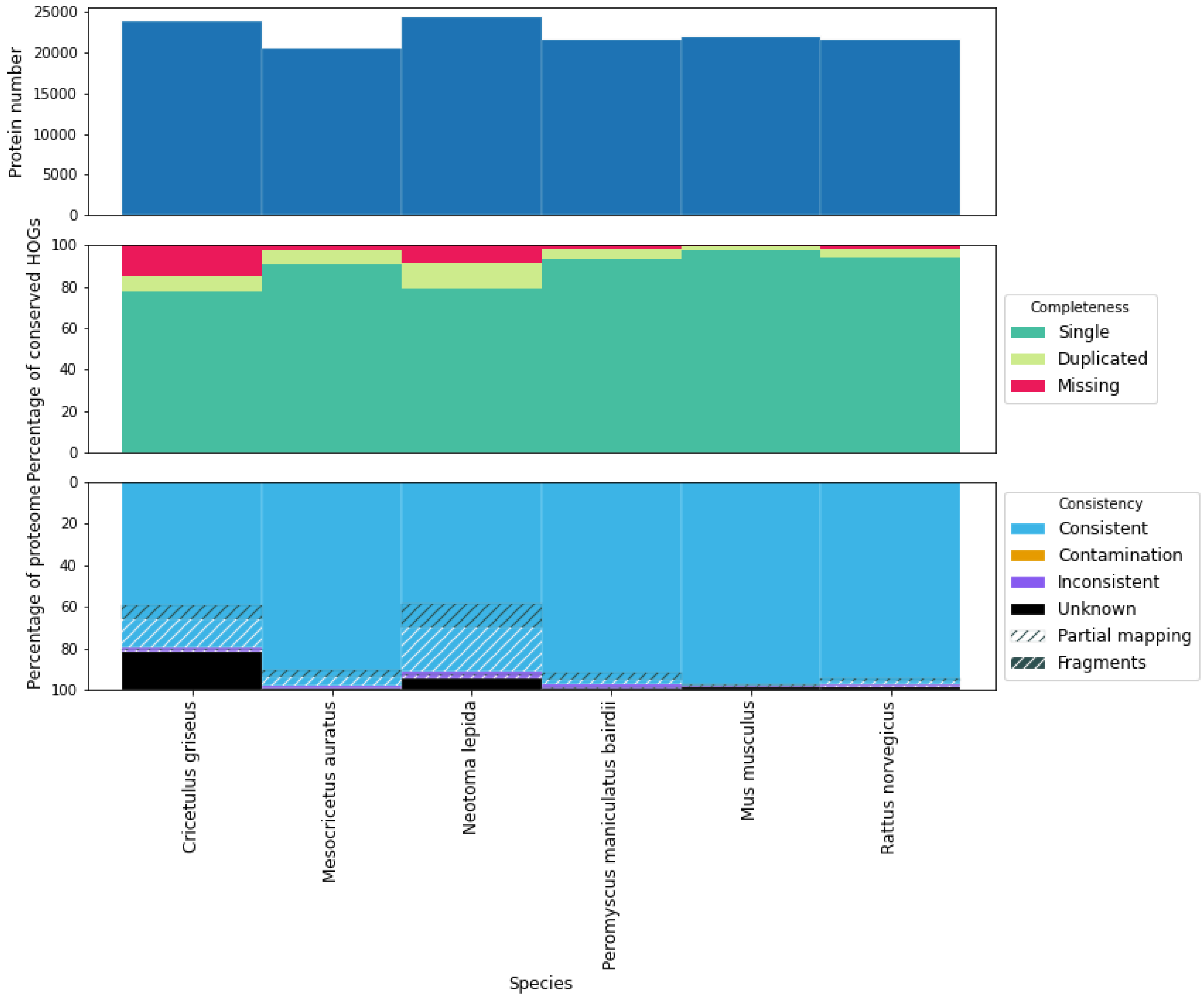

Supplementary Figure 23. OMArk results for proteomes from the *Myomorpha* clade.

Gallus gallus and *Galloanserea*

In the *Galloanserae* clade (Supplementary Figure 24), OMArk statistics are highly heterogeneous. All proteomes display some extent of either Partial mapping or Fragmented genes (minimum 1.87% Consistent Partial mapping and 0.63% Consistent Fragments for *Aythya fuligula*, which has the highest consistency). This is possibly due to the gene set included in the OMA database being imperfect, thus having an impact on the assessment. However, this bias is expected to have a similar impact over all proteomes. Both in terms of completeness and consistency, three proteomes from this clade stand out as more reliable: the chicken *Gallus gallus*, the pheasant *Phasianus colchicus,* and the tufted duck *Aythya fuligula.* The chicken shows an uncommonly high number of Duplicated families (10.05%) compared to the two others (0.82 and 0.86% respectively), which is reflected by the number of proteins. Other proteomes in this clade are less complete and have a higher proportion of fragmented gene models. This is, in part, explained by the use of different annotation methods (See above section - Analysis of avian proteomes).

For *Galloanserea*, the number of proteins is correlated (Pearson correlation:0.73) to the number of duplicated proteins detected in the proteome with the largest proteome also being the one with more duplications. It is only moderately correlated with total completeness (Pearson correlation: 0.57).

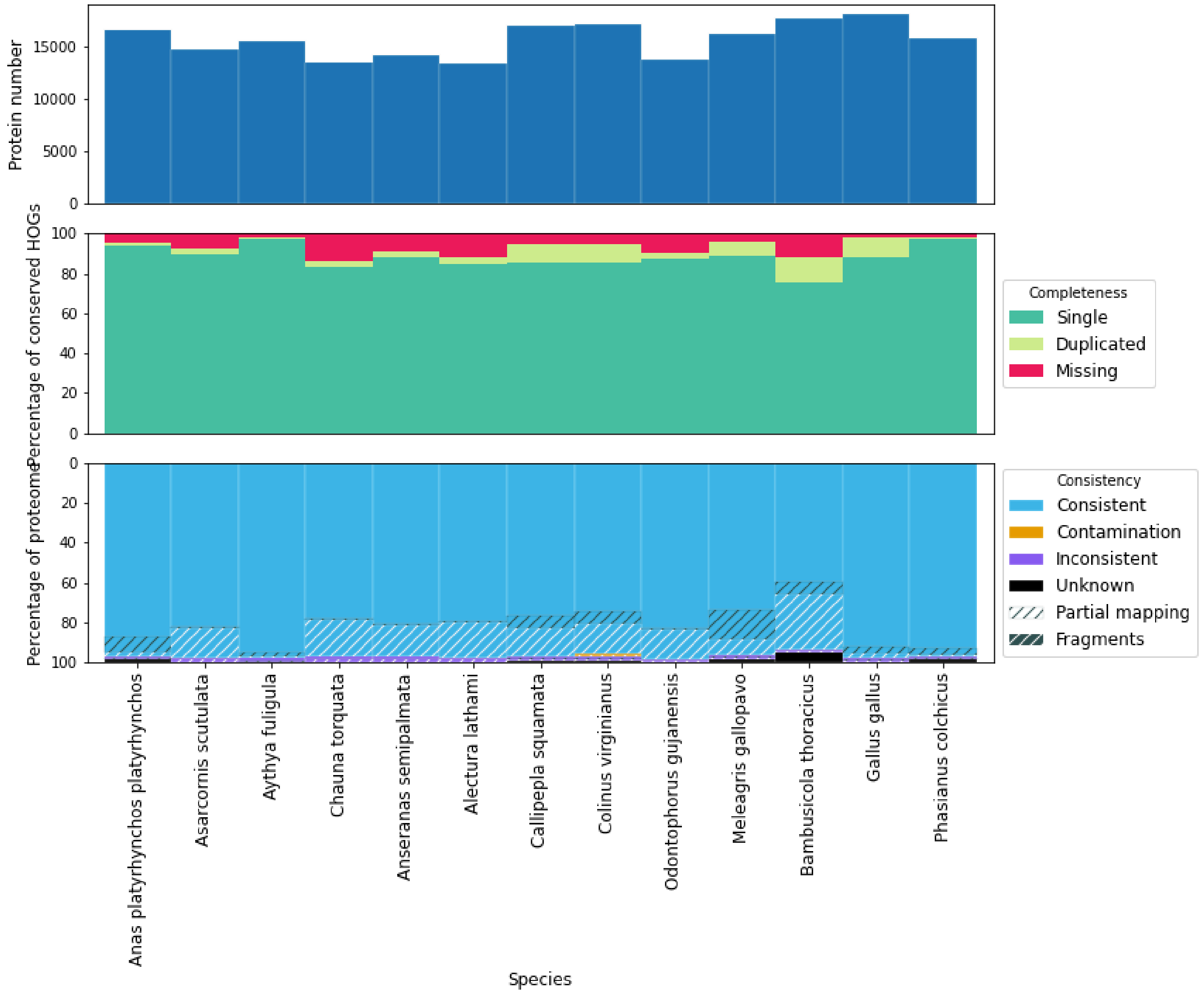

Supplementary Figure 24. OMArk results for proteomes from the *Galloanserae* clade.

Xenopus and *Amphibia*

Due to the low sampling of Amphibian genomes in the OMA database, OMArk data assessments for this clade are based on the *Tetrapoda* ancestral genomes (Supplementary Figure 25). It is thus expected to be less precise than other species where the ancestral lineage is more recent. The *Lithobates catesbeianus* proteome is missing most of its genes, however, this is likely due to an upload error of this proteome onto UniProt and it can be ignored for this analysis. In this example, *Xenopus tropicalis*, *Geotrypetes seraphini,* and *Microcaecilia unicolor* have a close amount of taxonomically Consistent gene placements (92-96%) with consistent gene structure (82-84%), and all have high completeness. However, *Xenopus tropicalis* is missing nearly twice as many genes as the others (7.72% versus 4.32% and 3.69%). This is unexpected as it is considered a model organism for this taxonomic range. Finally, *Xenopus laevis* has a high number of duplicated genes, as expected for a polyploid species, however, the proportion of Unknown genes (18.88%) is also higher than all other representatives of the clade (2-4%), suggesting issues with the annotation. Both values are likely contributing to its high number of proteins.

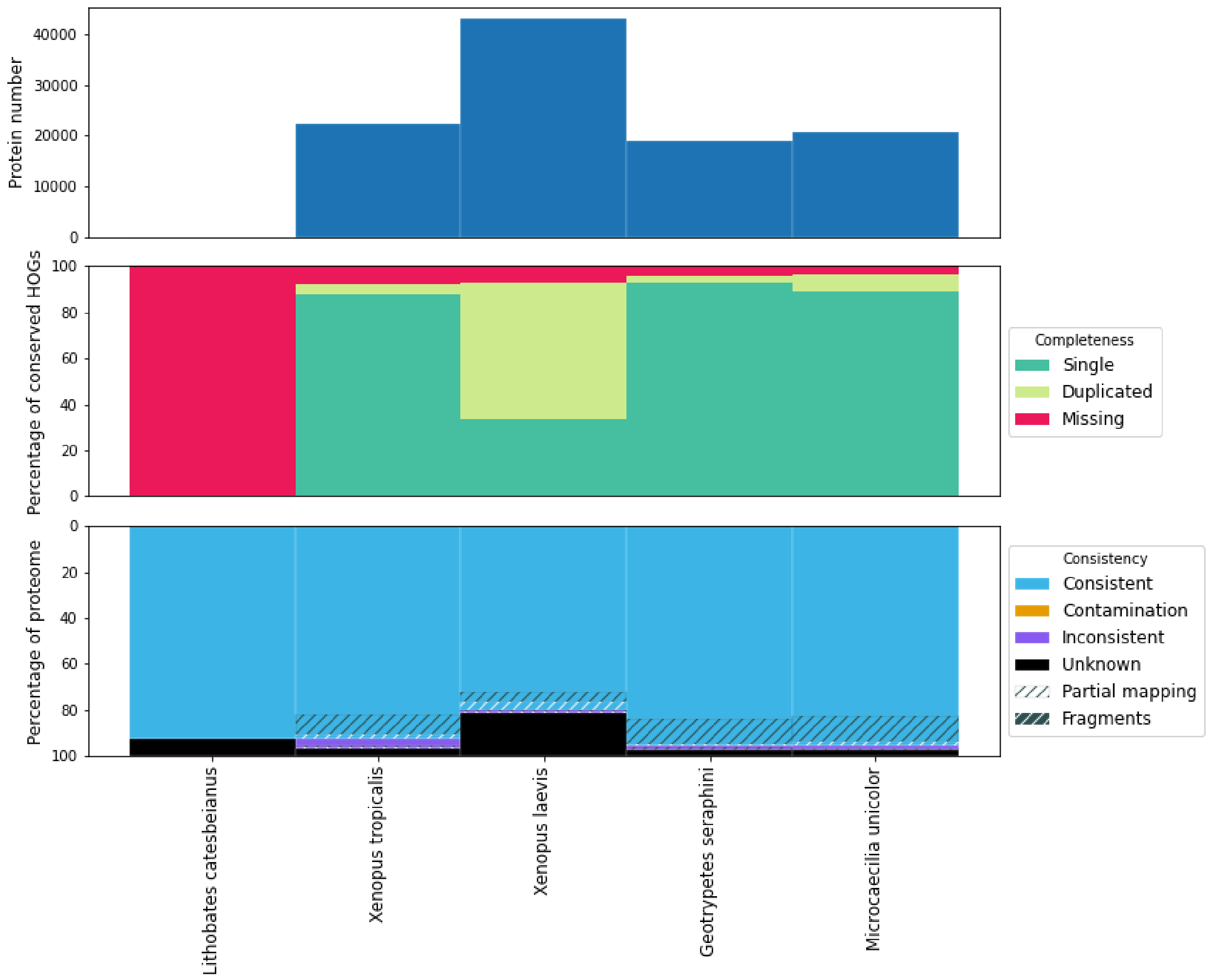

Supplementary Figure 25. **OMArk results for proteomes from the Amphibia clade.**

Zebrafish and *Otophysi*

OMArk results in *Otophysi* (Supplementary Figure 26) are heterogeneous, with all proteomes– including the best annotated ones– having at most 93% structurally and taxonomically Consistent proteins, with at least 3% of genes with divergent gene structure. As all proteomes appear to be impacted, this may be due to a limitation in the reference gene set. Still, *Danio rerio* as well as *Electrophorus electricus* and *Sinocyclocheilus grahami* are detected as the most consistent to known gene families in terms of overall gene model accuracy (between 89% and 93% taxonomically and structurally consistent genes) while all having less than 5% detected Missing genes. Species from the *Sinocyclocheilus* genus and *Carassius auraeus* all have the lowest proportion of missing genes and a high proportion of duplicated genes, likely due to a recent Whole Genome Duplication in the ancestor of these species [13]. Finally, we can note that the Mexican tetra *Astyanax mexicanus* proteome is highly complete but appears to have issues in terms of gene models when compared to other species from the same clade (9.6% Partial mapping and 4.48% Fragmented consistent genes).

In *Otophysi*, the total number of proteins is mostly correlated to the number of duplicated genes (Pearson correlation: 0.91) which is likely driven by the whole genome duplication. It is only moderately correlated with completeness (Pearson correlation:0.53), two species in particular

have protein number equal or higher than more complete species while having a high proportion of Unknown proteins and proteins with divergent structure (Partial mapping and Fragments).

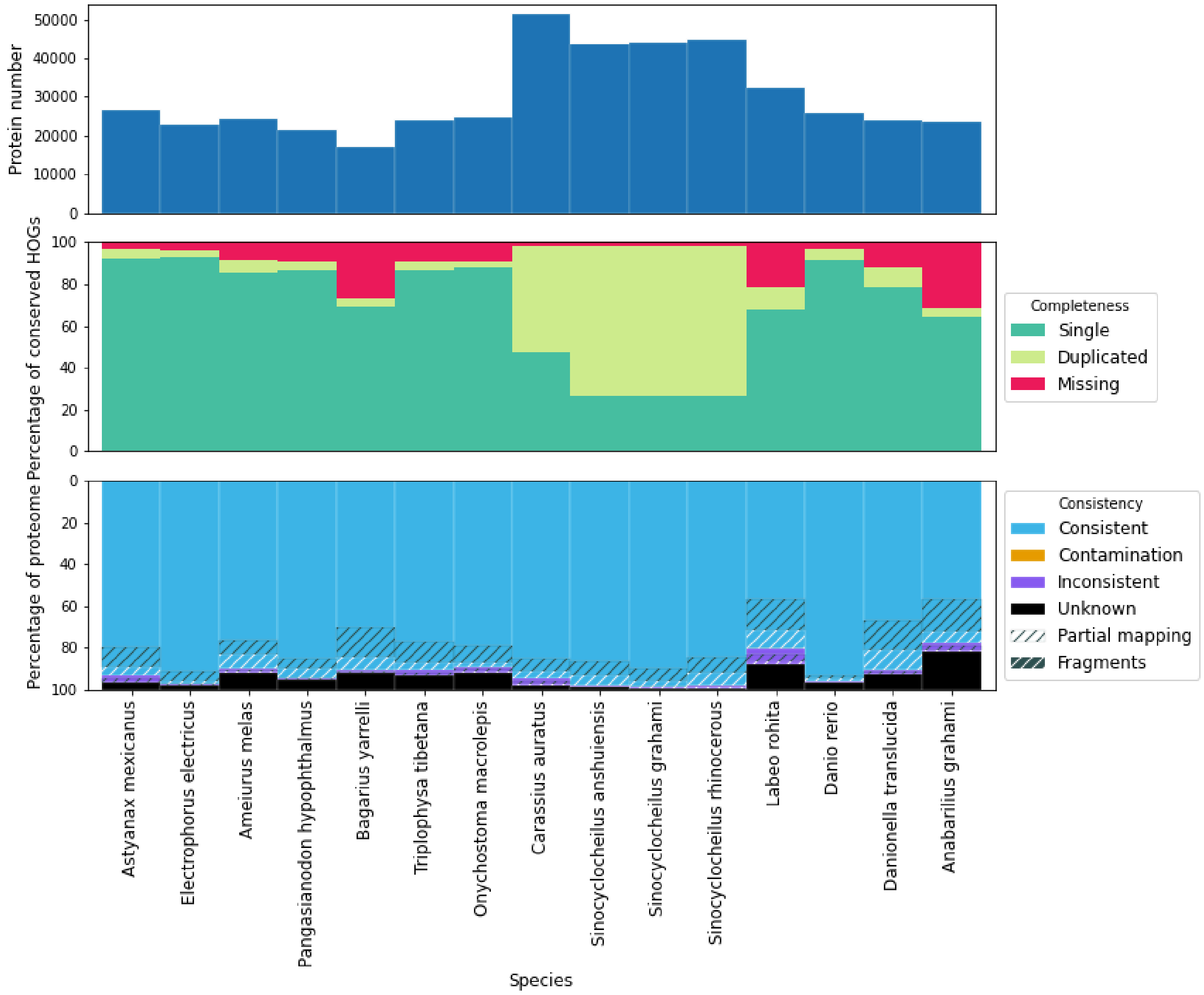

Supplementary Figure 26. OMArk results for proteomes from the *Otophysi* clade.

Drosophila melanogaster and *melanogaster* subdivision

Due to a high sampling in the *Drosophila* genus in the OMA database, the fruit fly *Drosophila melanogaster* is compared to the ancestral gene set of the *melanogaster* subdivision (Supplementary Figure 27). In terms of taxonomically and structurally Consistent proteins, *D. melanogaster* obtains distinctly higher measures than the other two species (96.20% non-partial, non-fragment Consistent genes against 85.15% and 82.72% for *D. sechellia* and *D. simulans,* respectively), despite all three species being present in our reference dataset. In comparison with *D. sechellia, D. melanogaster* has a higher proportion of Missing conserved HOGs (3.78% and 1.74%, respectively), which may be due to a higher number of gene losses as a domesticated species rather than incompleteness. Accordingly, when selecting the Diptera clade as a reference gene set for the same proteome, the proportion of core genes missing drops to 0.13%.

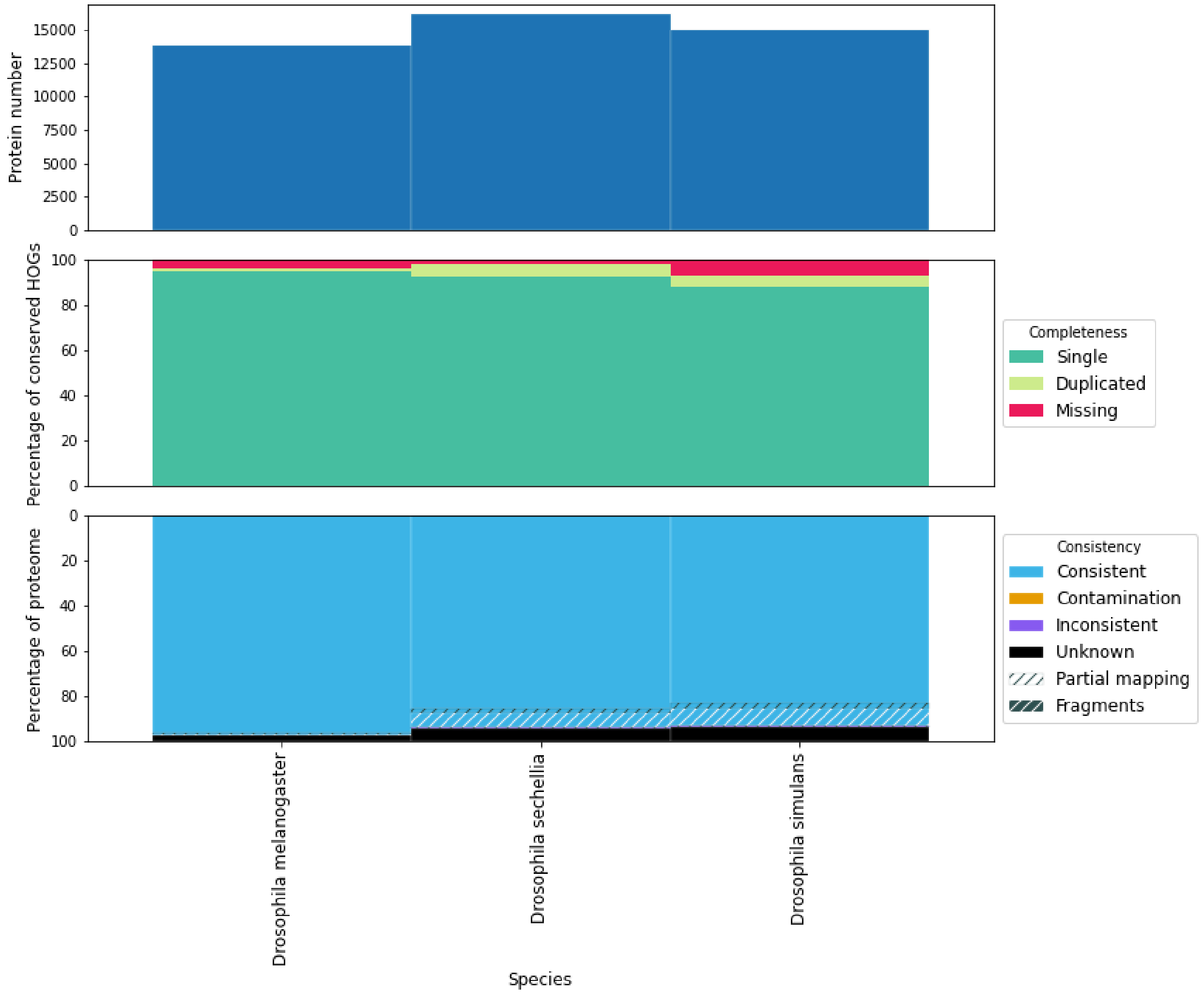

Supplementary Figure 27. OMArk results for proteomes from the "melanogaster subdivision" clade.

Caenorhabditis elegans and the *Caenorhabditis* genus

The *Caenorhabditis* genus (Supplementary Figure 28) is another example of high heterogeneity in terms of OMArk results but also in terms of proteome content, with a variation of gene number from 19,812 for *Caenorhabditis elegans* to 28,999 in *Caenorhabditis brenneri*. All of the species have a relatively high number of "Unknown" proteins, with a minimum of 12.30% for *Caenorhabditis elegans*. This corresponds to 2,437 sequences. This is not necessarily an indicator that so many genes are annotation errors, but may be linked to difficulty in finding homologs in nematode species. Similarly, the OMA Browser does not infer orthologous groups for 3,142 *Caenorhabditis elegans* genes. The numbers we obtain with OMArk are in line with it. The relative proportion of those Unknown genes in the proteome, however, can be a sign of different annotation quality.

Caenorhabditis elegans, the most studied and curated species in this dataset, has both the lowest number of missing genes (1.42%) and the highest proportion of taxonomically consistent genes (86.61%). This can not be explained by being present in our reference database since *briggsae*, *brenneri,* and *japonica* are as well but are marked as less consistent overall. Interestingly, the two other most complete proteomes, *Caenorhabditis briggsae* and *Caenorhabditis nigoni,* have a similar amount of Missing genes (2.52% and 2.55%), however *briggsae* appears to have more taxonomically and structurally Consistent genes (74.03%

Taxonomically and Structurally Consistent compared to 57.12% for *C. nigoni*), indicating this proteome might be more viable overall than the other non-*C. elegans* species.

In *Caenorhabditis,* the total protein number was mostly correlated with the number of Duplicated proteins.

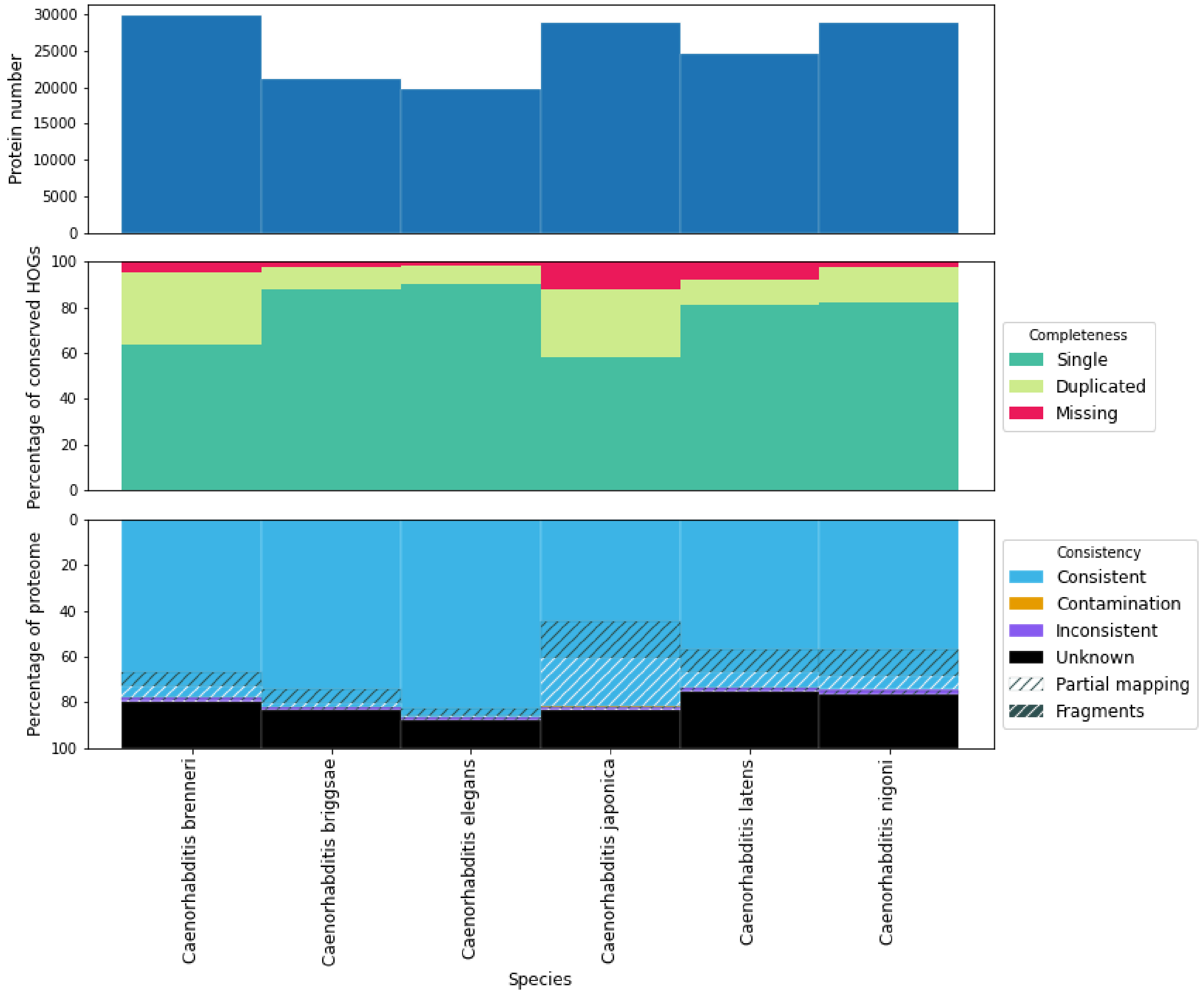

Supplementary Figure 28. OMArk results for proteomes from the Caenorhabditis clade.

Saccharomyces cerevisiae and *Saccharomycetacea*

Many species in the *Saccharomycetaceae* family (Supplementary Figure 29) exhibit high completeness (less than 1% missing genes) and high consistency (95%). However, some species, disseminated across the taxonomy, have more than 3% of missing genes and more than 10% Partial mapping genes. It is unclear if this is an annotation quality issue or a result of gene repertoire plasticity in fungi species, since these proteomes are the ones not represented in the OMA database. Nevertheless, the two other representatives of the *Saccharomyces* genus, *Saccharomyces arboricola* and *Saccharomyces kudriavzevii* can be unambiguously considered as incomplete in relation to close species, with 26.23% and 28.96% of missing Conserved HOGs, consistent with their low number of coding genes.

Saccharomyces cerevisiae stands out from the other species by its perfect completeness (0% missing) and its low level of Fragments (0.66%) and Partial mapping genes (0.53%). It has, however, a relatively high number of Unknown genes (380, 6.28%). This can be attributed to the

fact that *S. cerevisiae* has the highest number of genes compared to the other species (6,050 proteins in *S. cerevisiae*; 5,385 for the second largest). As *S. cerevisiae's* genome is extensively characterized, these are probably actual translated genes but with no, or difficult to detect, homology to other genes. Similarly, in the OMA Browser, 755 genes of this species are also not in orthologous groups.

In *Saccharomycetacea*, number of proteins was strongly correlated with completeness (Pearson correlation: 0.85), and somewhat with the number of Unknown proteins (Pearson correlation:0.66).

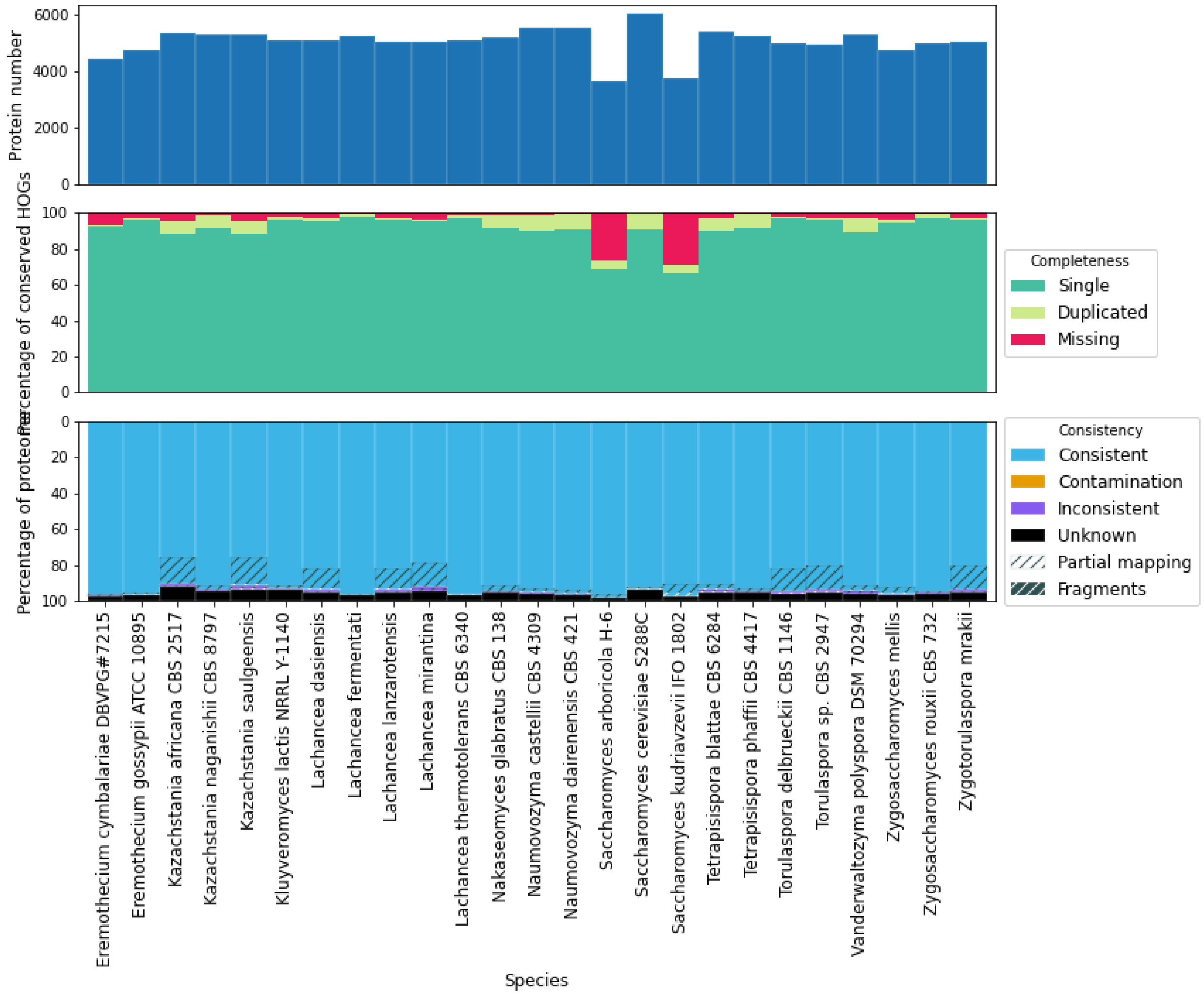

Supplementary Figure 29. OMArk results for proteomes from the Saccharomyces genus.

Arabidopsis thaliana and *Brassicaceae*

We compare *Arabidopsis thaliana* to other species in the *Brassicaceae* clade (Supplementary Figure 30). As expected, its proteome ranks among the most complete in the dataset according to OMArk (99.04% completeness), along with both subspecies of *Brassica rapa* (98.89% and 98.47%). The same three proteomes are also among the best in terms of taxonomically and structurally Consistent genes (90.97% for *A. thaliana,* 89.28% for *B. rapa*, 93.65% for *B. rapa subspecies pekinensis*. Every species in this clade displays some amount of Duplicated genes, especially high among species from the *Brassica* genus, which is likely due to high ploidy levels in those species [14]. Species from the same genus display heterogeneous levels of completeness

and/or different consistency. As an example, even though *Arabidopsis lyrata* is fairly complete (98.14%), its proportion of taxonomically and structurally Consistent genes is 81.27%, nearly 10% lower than its sister species.

In *Brassicaecea,* the total numbers of proteins in the proteome was mainly correlated to the number of Duplicate, as expected with the aforementioned high ploidy level of species included.

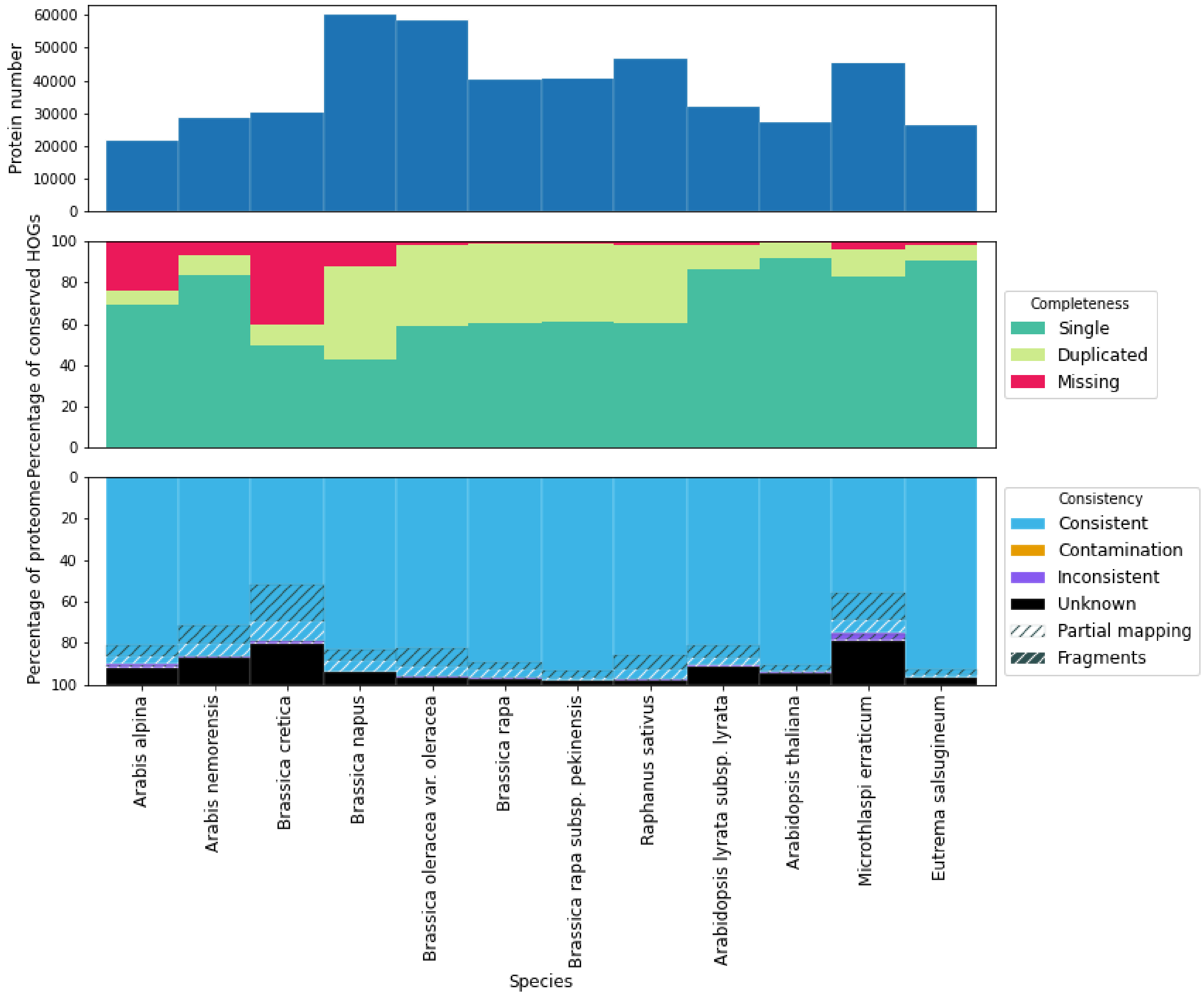

Supplementary Figure 30. OMArk results for proteomes from the Brassicaceae clade.

Impact of the source of OMA's proteomes on OMArk results

Global comparison of Ensembl and NCBI annotations

The OMA Browser, on which OMArk is built, incorporates proteomes from different sources with methods of annotation. For Eukaryotes, which are the expected query for OMArk, sources are biased toward Ensembl (Supplementary Figure 31). In total, Ensembl proteomes make up 74.2% of all proteomes in OMA. Since OMArk relies on homology information for these proteomes, there's potential for a bias, where annotations originating from the same source (Ensembl) yield better results, while those from different sources may receive lower quality scores.

To test whether OMArk was biased by the source of proteomes, we compared OMArk results for 1200 proteomes for which we could find an annotation version in the NCBI database and in the Ensembl database (data in Supplementary Table 6). To ensure that our comparison wasn't affected by other external factors, we only considered proteomes associated with the same assembly. In this dataset, 321 have proteomes from the same species in the OMA Browser that came originally from the Ensembl database, while the 879 others either come from different sources or are not in the OMA database.

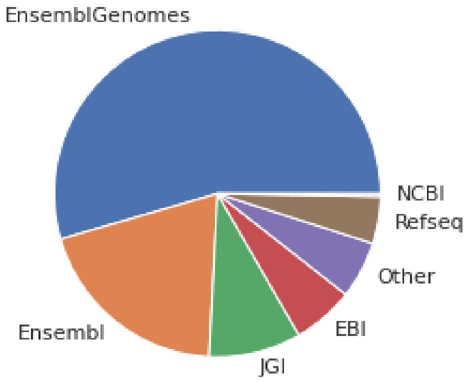

Supplementary Figure 31. Distribution of sources for eukaryotic proteomes in the Nov22 release of the OMA Browser. OMA proteomes mainly come from Ensembl. Ensembl stands for the main Ensembl release, and EnsemblGenomes from any of the clade-specific databases (Ensembl Plants, Ensembl Fungi; Ensembl Metazoa, Ensembl Protists)

Supplementary Figure 32 shows differences between annotations of the same assembly in NCBI (GenBank+Refseq) and Ensembl, highlighting whether proteomes from these species in OMA originally come from Ensembl originally. Supplementary Table 8 provides a summary of the average discrepancies between NCBI and Ensembl for proteomes from the same species. The table further details these variances for subsets where a proteome is either sourced from Ensembl or not. We performed a one-sided Wilcoxon signed-rank test in order to assess the significance of these differences over the samples.

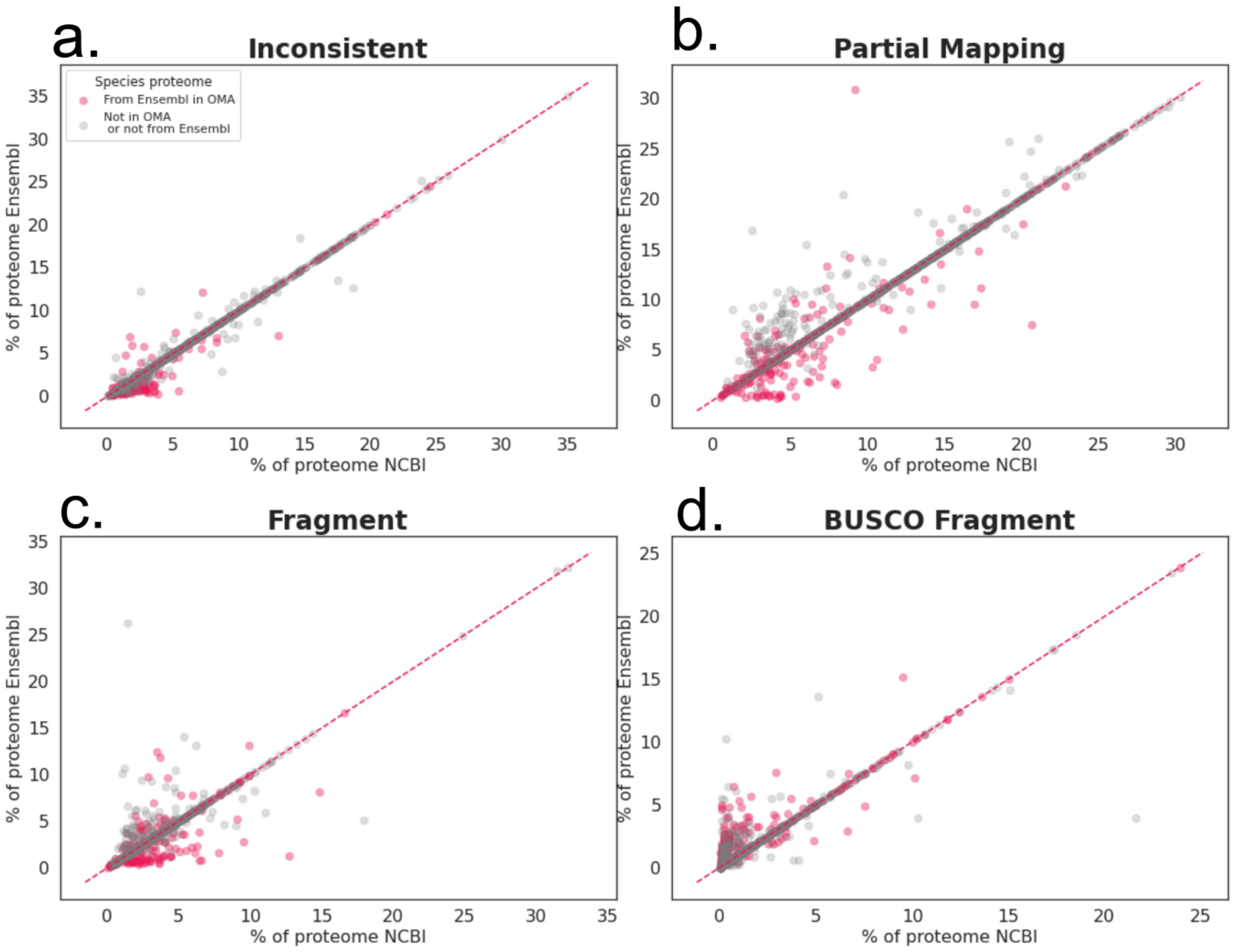

Supplementary Figure 32. Comparison of OMArk/BUSCO results on Ensembl and NCBI. Each panel corresponds to a different OMArk or BUSCO statistic. Each dot is one given assembly, colored in red if the species has an annotation from Ensembl included in OMA. X-axis corresponds to results on NCBI proteomes, and Y-axis to results on Ensembl proteomes. **a.** OMArk Inconsistent gene proportion. **b.** Partial mapping statistics of OMArk (aggregated on Consistent, Inconsistent, and Contamination). **c.** Fragment statistics of OMArk (aggregated on Consistent, Inconsistent, and Contamination). **d.** Fragment statistics of BUSCO.

Difference NCBI-Ensembl (%)	Complet-eness	Consi-stent	Inconsi-stent	Unknown	Partial mapping	Fragment
**Total OMArk**	1.39*** $(p=2*10^{-16})$	0.59*** $(p=4.7*10^{-5})$	0.09*** $(p=1.5*10^{-9})$	-0.64*** $(p=1.2*10^{-9})$	-0.18*** $(p=1.9*10^{-4})$	-0.05* (p=0.048)
Species in OMA from Ensembl	0.59*** $(p=1.2*10^{-5})$	0.77 (p=0.65)	0.29*** $(p=4.8*10^{-14})$	-1.06* (p=0.01)	0.28*** $(p=4.6*10^{-4})$	0.27*** $(p=1*10^{-4})$
Species not in OMA or not from Ensembl	1.69*** $(p=3.8*10^{-13})$	0.52*** $(p=8.8*10^{-11})$	0.01 (p=0.38)	-0.49*** $(p=3.5*10^{-10})$	-0.35*** $(p=2.4*10^{-14})$	-0.17*** $(p=3.4*10^{-10})$
**Total BUSCO**	2.08*** $(p=1.2*10^{-42})$	/	/	/	/	-0.22*** $(p=9.8*10^{-87})$
Species in OMA from Ensembl	1.88*** $(p=3*10^{-22})$	/	/	/	/	-0.47*** $(p=8.4*10^{-8})$

Species not in OMA or not from Ensembl	2.15*** (p=3.1*10^{-22})	/	/	/	/	-0.12*** (p=8.5*10^{-86})

Supplementary Table 8. Average difference of OMArk and BUSCO results of Ensembl and NCBI annotation of the same assemblies. Values are positive if they are greater for NCBI than from Ensembl and are colored in green if the result is favorable to NCBI. Stars correspond to significance of a one-sided Wilcoxon signed rank test- with the alternative hypothesis depending on the sign of the mean difference (H1=NCBI greater than Ensembl; if positive and the reverse if negative) . *p<0.05, **p<0.01, ***p<0.001. The exact p-value is indicated between parenthesis in each cell.

On average, the disparity between annotations from differing sources is minimal, generally lower than a 1% difference, and never exceeding 2%. Proteomes from NCBI tend to exhibit greater completeness, a larger proportion of Consistent genes, and fewer Unknown genes (those lacking known homologs). Furthermore, NCBI proteomes have an even smaller percentage of partial mapping genes and fragments than those from Ensembl. Thus, even though most of OMA's data comes from Ensembl, NCBI's proteomes are ranked by OMArk as having slightly higher quality. In BUSCO results, the trend is similar with genomes from NCBI being marked as more complete and less fragmented - although the amplitude of both being higher in BUSCO.

However, when looking at the subset of these data for which the species of interest is already represented in OMA by a proteome coming from Ensembl, the picture is slightly different. NCBI proteomes are still detected as significantly more complete, though with a lower margin than the main dataset. BUSCO, however, reports a difference in completeness similar to the one of the main dataset. In terms of consistency, the NCBI proportion of consistent genes is still higher than for the main set but not significantly according to our Wilcoxon test (p-value=0.65), possibly because of heterogeneity between proteomes. Unknown genes are also detected as lower for the NCBI proteomes than Ensembl's.

Finally, for this set of species in which the proteome in the OMA database comes from Ensembl, NCBI appears overall significantly richer in genes of the Inconsistent categories, and contrary to the results for the full set of proteomes, the proportion of Partial Mapping genes and Fragments are also higher in the NCBI proteomes. This is in contrast to BUSCO, which registers even more fragments in the Ensembl genomes on the same dataset. The increased proportion of detected Inconsistent genes, Partial mapping, and Fragments can likely be attributed to the bias in sourcing from the OMA database. Higher inconsistent proportions may be due to the fact that true gene models are somehow not represented in OMA for a clade of interest because they are missing from the Ensembl gene set, while Partial mapping and Fragments may be due to divergence in annotated gene structure.

In conclusion, a bias due to the source of the proteome appears to significantly affect OMArk's results when comparing proteomes from different sources - especially in terms of Inconsistent, Partial mapping, or Fragmented genes. Yet, the intensity of this bias seems relatively minor, at most a few percentage points, and doesn't overshadow the enhanced completeness and reduced Unknown genes in NCBI's proteome. We could not detect this trend when using BUSCO for comparing these proteomes, further confirming the differences reported by OMArk stem from dataset biases. However, it is plausible that BUSCO too is affected by a similar data origin bias, given most proteomes in OrthoDB are imported from NCBI.

Danio rerio's case studies

As an example of the possible impact of the source of the proteome present in the database, we focused on the different public annotations available on public databases: Ensembl, UniProt, and

NCBI. As the proteomes from zebrafish and all but two other Teleost fishes are from Ensembl, a bias could favor proteomes from this source against ones from other sources. For UniProt, we chose to compare two versions of the proteomes: the one from the 2022 Reference Proteome, the main dataset for this paper, and a more recent version downloaded in August 2023. The 2022 proteome in UniProt was derived mainly from Ensembl data, while the most recent one is mainly derived from NCBI's.

The four proteomes studied vary by their protein number, with the one from Ensembl having 30,313 proteins and the one from NCBI 28,638, compared to the much smaller 25,706 and 26,249 from UniProt (Supplementary Figure 33). This can be explained by the fact that proteomes from NCBI and Ensembl also include variant sequences from Alternative loci not included in the Primary Assembly in their FASTA files representing the proteomes. This also likely explains the higher proportion of duplicate genes in these (18.03% in Ensembl and 6.76% in NCBI) compared to UniProt proteomes (4.85% and 4.09%).

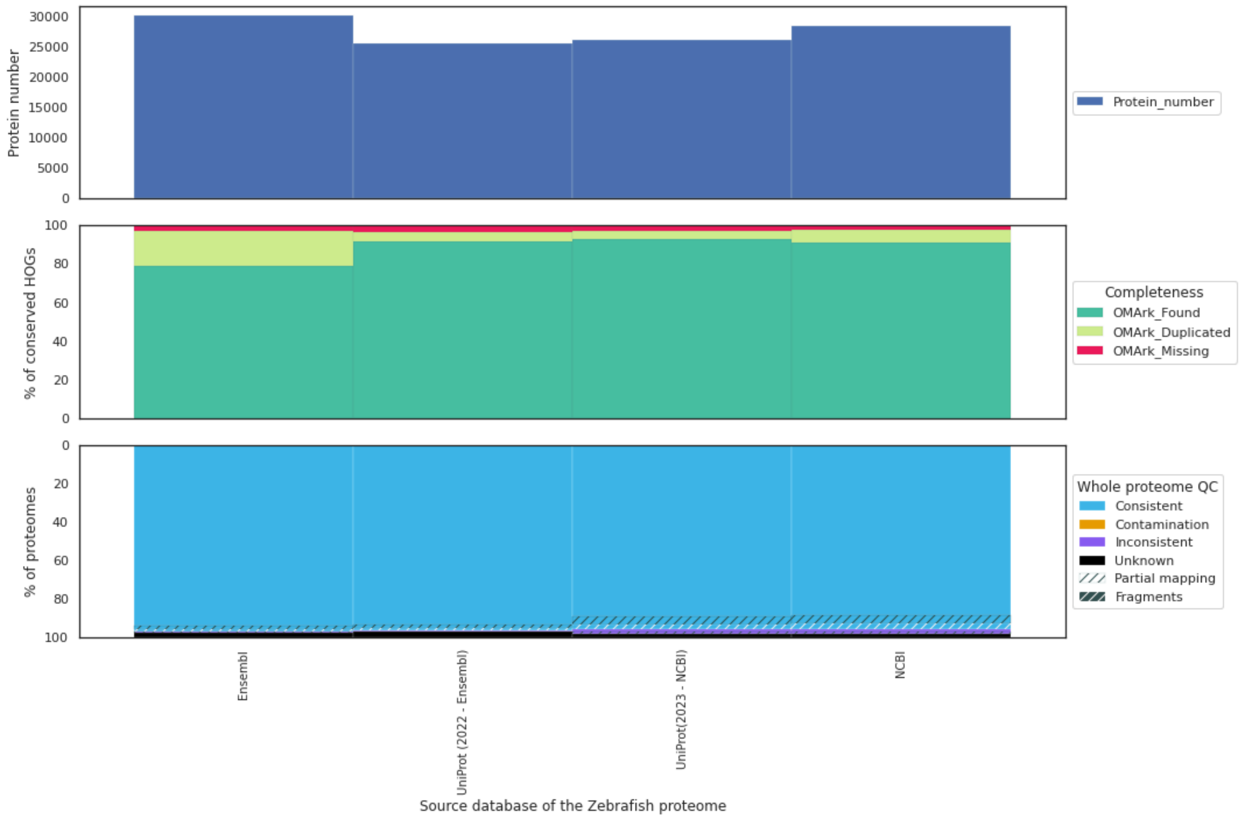

Supplementary Figure 33. OMArk results for four different proteomes of zebrafish. All proteomes are derived from the GRCz11 of the zebrafish genomes. Each row is a different proteome from Ensembl (Danio_rerio GRCz11) UniProt (UP0000437_7955; 2022 and 2023) and NCBI (GCF_000002036.6).

According to OMArk, the proteome from NCBI is the most complete, with only 2.13% Missing genes. The corresponding NCBI-derived UniProt 2023 proteome follows closely with 2.63% Missing genes. The Ensembl proteome (3.04% Missing) and the older Ensembl-derived 2022 UniProt proteome (3.37% Missing) are slightly less complete overall. In terms of consistency, the two proteomes derived from NCBI also have a lower proportion of genes with no known homologs (NCBI: 1.98% and UniProt 2023 1.94%) than the Ensembl derived ones (Ensembl 2.69% and UniProt 2022: 3.04%) which possibly indicates a higher proportion of erroneous genes in the later. Interestingly, NCBI-derived proteomes exhibit a higher proportion of taxonomically Consistent Partial mapping (NCBI: 4.17%, UniProt 2023: 4.30%; Ensembl: 1.53%,

UniProt 2023: 1.87%) and fragmented genes (NCBI: 2.96%, UniProt 2023: 2.57%; Ensembl: 1.18%, UniProt 2022: 1.48%), as well as taxonomically Inconsistent genes (NCBI: 2.36%, UniProt 2023: 2.26%; Ensembl: 0.70%, UniProt 2023: 0.65%) which indicate proteins likely to represent *bona fide* homologs genes that are not known to OMA in these clades or with this structure.

Drawing from this data, for zebrafish, as is the case globally, sourcing the majority of proteomes from this clade might introduce a minor bias when evaluating the structural and taxonomical consistency of genes. However, this potential bias doesn't overshadow the evident higher completeness and broader proportion of genes with known homologs evident in NCBI proteomes.

Assembly and annotation comparisons

We obtained the list of species with either an annotation or assembly change for the two previous releases of Ensembl metazoa (53 and 54)[15], and computed OMArk results for the previous and newly released versions of each one. This corresponds to 18 pairs of proteomes from protostomian species; 11 resulting from an assembly change and 7 (only nematode species) from an annotation change. All the results are available in Supplementary Table 5 and shown in Supplementary Figures 34-51.

Assembly comparisons

The assembly updates in Ensembl often lead to a less fragmented (higher contig N50) or slightly longer assembly (higher overall assembly length), except in the case of *Acyrthosiphon pisum* (Supplementary Table 9). Three assemblies have an increase in contig N50 of more than 100 fold: *Sarcoptes scabie*, *Solenopsis invicta,* and *Bombyx mori* while *Schistosoma mansoni* and *Crassostrea gigas* have an increase of more than 10% of total assembly length.

Changes in proteomes resulting from a different assembly manifest as important differences in the number of reported protein-coding genes, with all but one leading to a change of more than 500 in the number genes. In OMArk, this is reflected as a minor decrease in completeness (less than 2%) for 4 species, a minor increase for 2 species, and an increase of several percent for the 5 others, up to 22.94% in the most extreme case (*Teleopsis dalmanni*) (Supplementary Table 9, Supplementary Figures 34-44).

Interestingly, an increase in completeness was not always nor significantly associated with an increase in the number of proteins (Pearson correlation: 0.59, p-value:0.054), with *Glossina fuscipes* gaining 5% of completeness despite having 7,730 fewer protein-coding genes and *Bombyx mori* gaining 2.38% completeness with 742 fewer genes. Another notable case is the latest *Acyrthosiphon pisum* assembly– it is only 0.71 less complete with although it has 17,918 fewer genes. Similarly, completeness shows even less association with the increase of the assembly length in itself (Pearson correlation: 0.05, p-value: 0.89).

The newest assembly also led to a decrease in proportion of Duplicate conserved genes in all but four cases, likely corresponding to a decrease in the proportion of fragmented genes or improved phasing with better genome coverage. For the other species, the increase in Duplicated genes is slight for *Pristionchus pacificus* (0.45%), *Sarcoptes scabiei* (0.21%), and *Schistosoma mansoni* (1.72%) but larger for *Teleopsis dalmanni* (+11%). As the selected

Conserved HOGs are not necessarily conserved in a single copy, this is not enough to conclude that errors were introduced in the novel assembly. However, the *T. dalmanni*'s increase in Duplicated genes is so drastic, it is indicative of fragments or spurious duplicates.

In terms of consistency assessment, the new assembly leads to a higher proportion of Taxonomically Consistent genes in 9 cases out of 11. The highest increases are detected in species for which the number of proteins decreases the most, namely: *Acyrthosiphon pisum* (-17,918 proteins, +12.77% Taxonomically Consistent genes), *Glossina fuscipes* (-7,730 proteins, +27.77% Taxonomically Consistent genes), *Bombus impatiens* (-5,264 proteins, +12.09% Taxonomically Consistent genes) and *Danaus plexippus plexippus* (-2,023 proteins, +7.64% Taxonomically Consistent genes). Together with the reported increase or low decrease in completeness, this indicates that the newest assembly leads to a better quality gene set overall. Surprisingly, while the decrease in the proportion of taxonomically Consistent Fragments is highly correlated to an increase in contig N50 (Pearson correlation: 0.85, p-value:0.002), it has only a non-significant correlation with the proportion of Taxonomically and Structurally Consistent genes (Pearson correlation: -0.35, p-value: 0.32).

In contrast to the others, the updated assembly of *Crassostrea gigas* leads to a decrease in the proportion of taxonomically Consistent genes, together with an increase in the number of genes and im completeness (+4,239 proteins, -4.28% Taxonomically Consistent genes) meaning that as a whole, the added genes show lower agreement with known Lophotrochozoan gene families and contain likely artifactual sequences.

Evidence of improved gene set quality in most assemblies is more pronounced when examining the proportion of taxonomically and structurally Consistent genes. In 9 out of 11 cases, this proportion increases, often surpassing the rise in observed taxonomically consistent genes alone. Exceptions to this trend are *Crassostrea gigas, Sarcoptes scabiei* and *Schitosoma mansoni*. Overall, our results show that new assemblies often result in more accurate gene models (Supplementary Table 9).

Finally, the contamination detected by OMArk in different assemblies of the same species varies between assemblies. In *Schistosoma mansoni*, the contamination by *Trypanosoma* is no longer detected in the gene set of the updated assembly. In the cases of *Acyrthosiphon pisum* and *Glossina fuscipes,* the number of detected contaminant proteins is reduced in the newer assembly, implying that either some contamination still exists in the current or there is a case of horizontal gene transfer in these genomes. We notice one case (*Teleopsis dalmanni*) where OMArk detects contamination from species of the Bacteroidetes taxa, only on the updated assembly, indicating contamination introduced during resequencing.

Species	Completeness change (%)	Structurally consistent gene change (%)	Contig N50 ratio	Assembly length ratio
Solenopsis invicta	-0.24	2.32	445.2	0.94
Bombus impatiens	-1.21	17.34	1	0.99
Glossina fuscipes	5.05	30.99	1.12	1.04

Pristionchus pacificus	4.68	1.38	N/A	N/A
Sarcoptes scabiei	-0.52	-0.114	177.25	1.00
Bombyx mori	2.38	11.48	786.78	0.95
Danaus plexippus plexippus	0.01	13.78	1.70	1.00
Acyrthosiphon pisum	-0.73	21.06	0.91	1.00
Schistosoma mansoni	0.20	3.63	69.59	1.12
Crassostrea gigas	4.38	-9.16	50.08	1.16
Teleopsis dalmanni	22.74	9.82	0.97	1.00

Supplementary Table 9. Summary table of OMArk quality assessment of the percentage of change from previous to current assembly release of Ensembl Metazoa. Most proteomes increased in completeness or consistency in the newest release. Green color indicates an improvement, and red indicates a decrease in quality, based on OMArk.

By providing statistics about the presence of conserved genes and consistency of the overall gene sets, OMArk enables the detection of change along both axes. Over all proteomes, OMArk detects an increase in either completeness and proportion of taxonomically and structurally consistent genes in the new assembly in all but one case *(Sarcoptes scabiei*). Considering both axes can be particularly useful when they give contradicting information: for example, we notice that the updated proteome of *Bombus impatiens* is marked as slightly less complete than the previous version, but has higher consistency measures. However, *Crassostrea gigas*' newer proteome is more complete but exhibits an overall lower consistency. This nuance would be lost when comparing completeness alone.

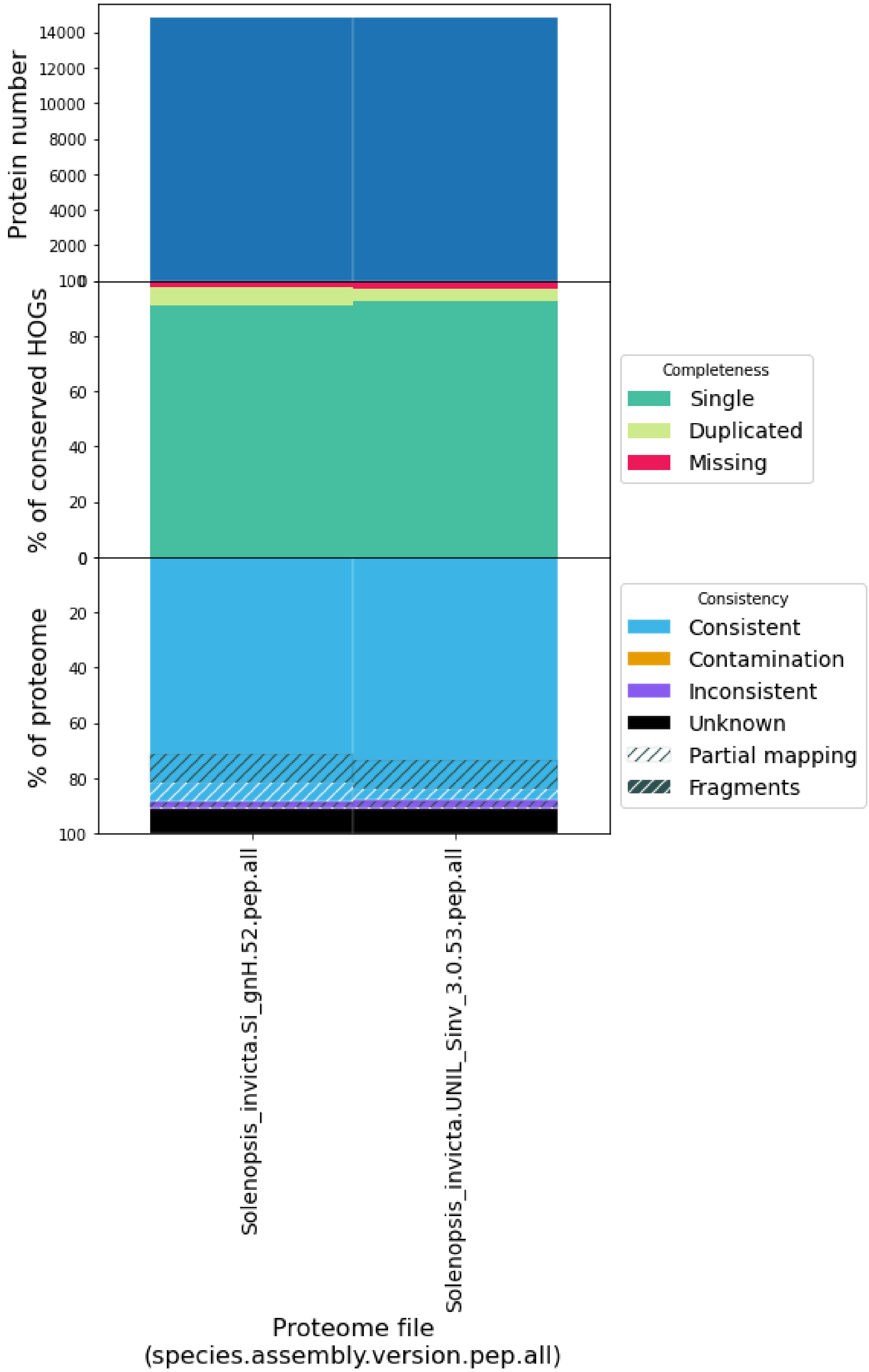

Supplementary Figures 34. OMArk comparison of different versions in *Solenopsis invicta* assemblies. Left bar plot corresponds to the proteome version in Ensembl Metazoa 52. Right bar plot is the proteome available in Ensembl Metazoa 53 and onward.

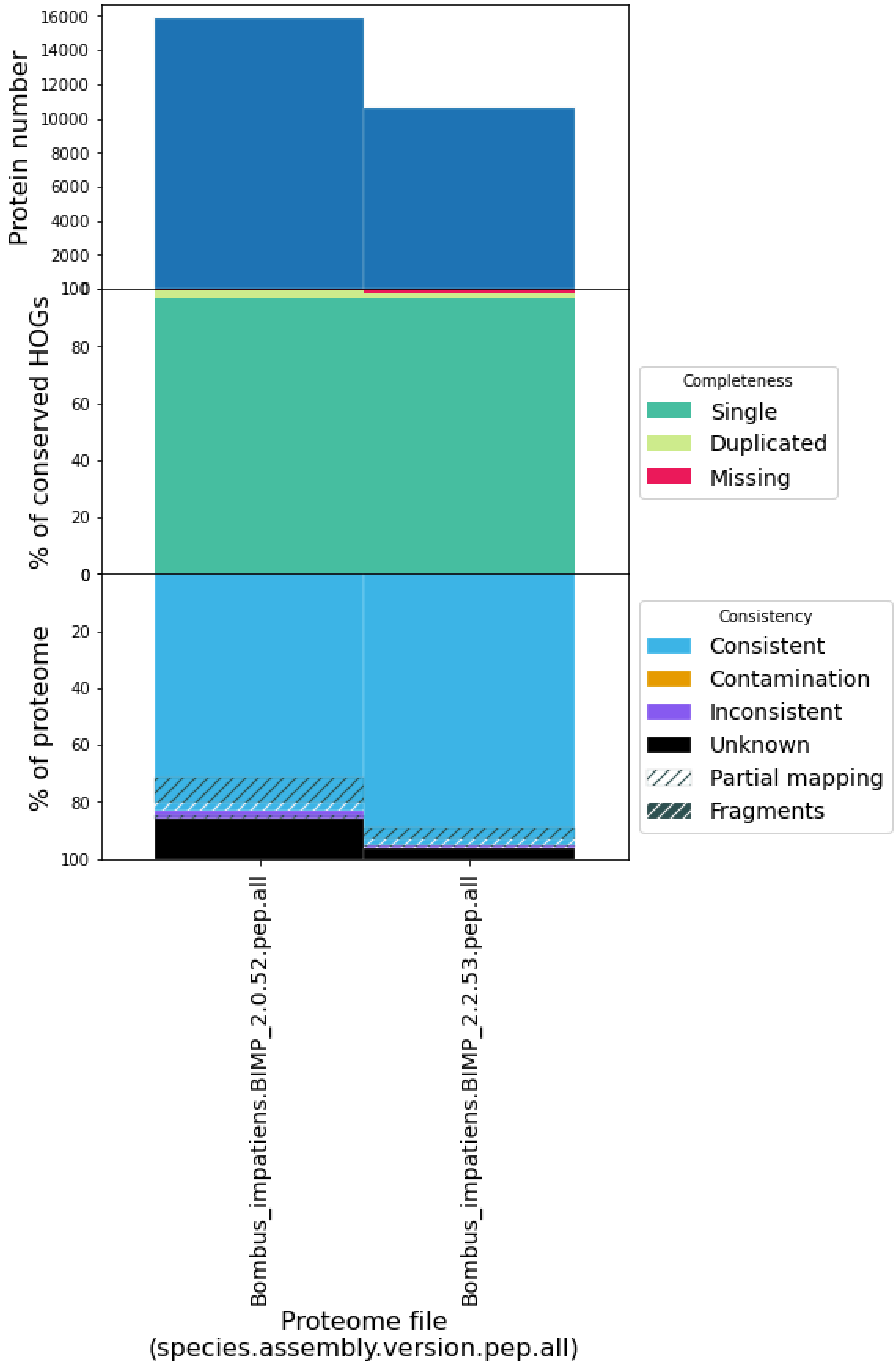

Supplementary Figures 35. OMArk comparison of different versions in *Bombus impatiens* assemblies. Left bar plot corresponds to the proteome version in Ensembl Metazoa 52. Right bar plot is the proteome available in Ensembl Metazoa 53 and onward

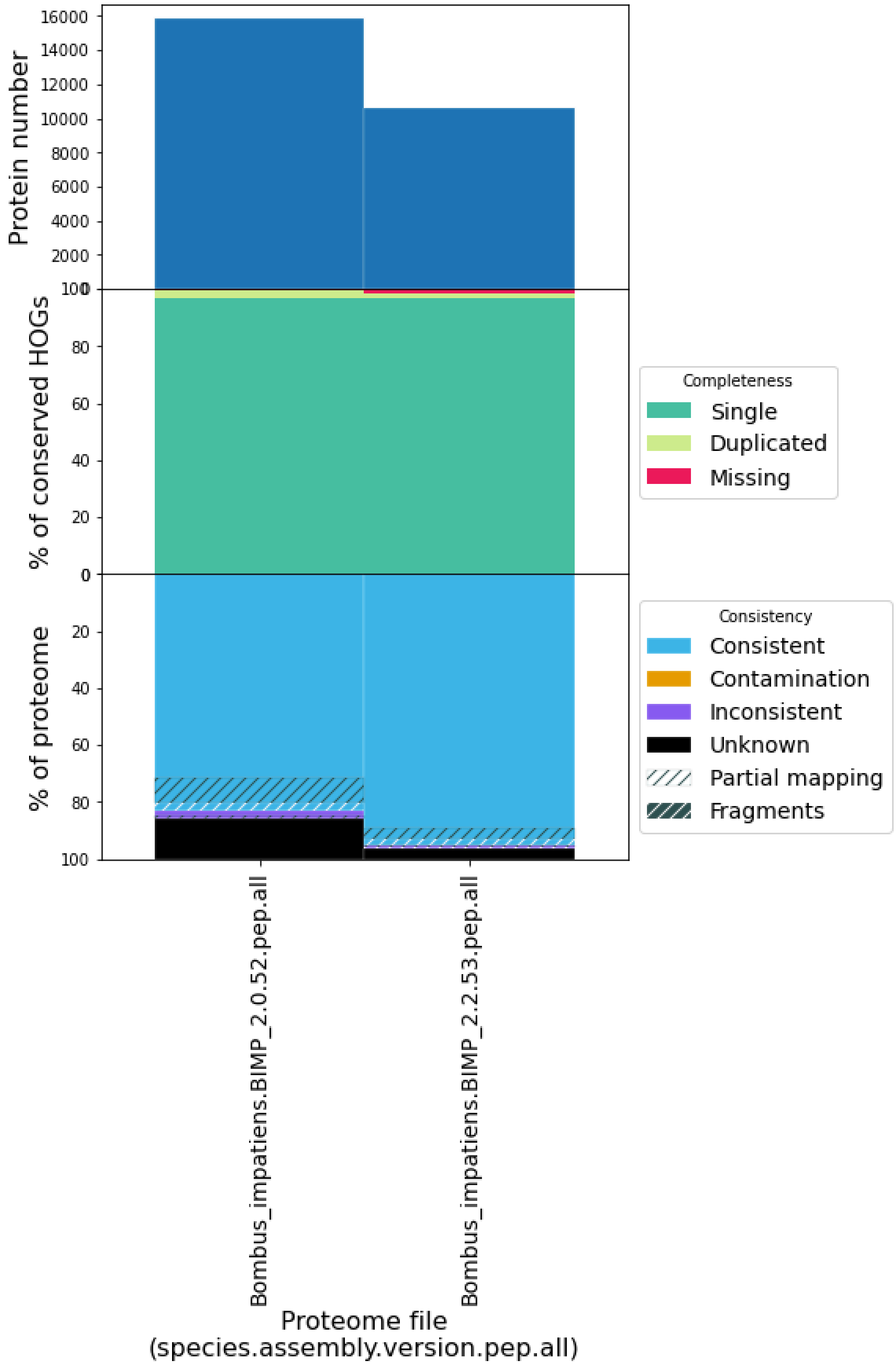

Supplementary Figures 36. OMArk comparison of different versions in *Glossina fuscipes* assemblies. Left bar plot corresponds to the proteome version in Ensembl Metazoa 52. Right bar plot is the proteome available in Ensembl Metazoa 53 and onward

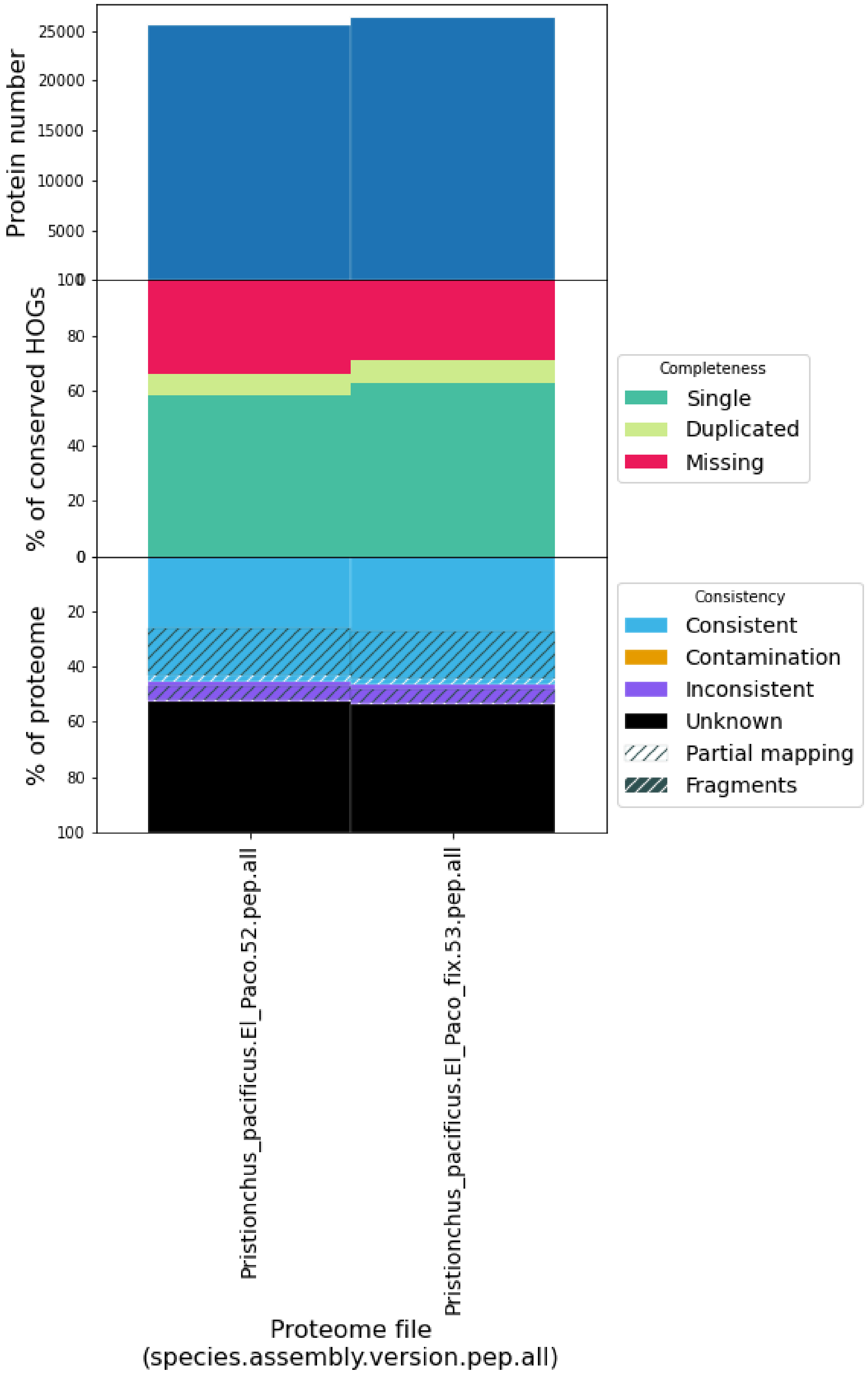

Supplementary Figures 37. OMArk comparison of different versions in *Pristionchus pacificus* assemblies. Left bar plot corresponds to the proteome version in Ensembl Metazoa 52. Right bar plot is the proteome available in Ensembl Metazoa 53 and onward

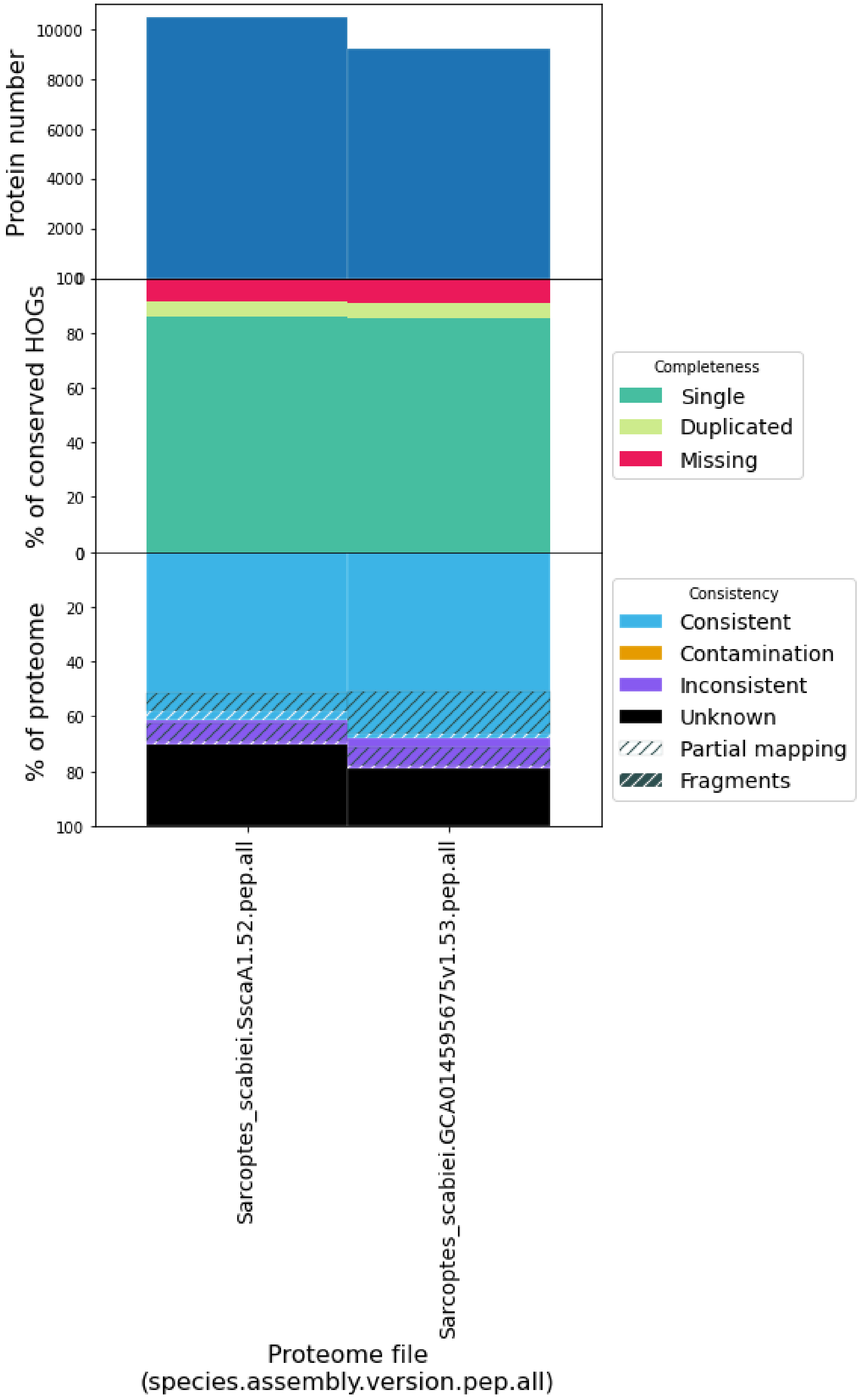

Supplementary Figures 38. OMArk comparison of different versions in *Sarcoptes scabiei* assemblies. Left bar plot corresponds to the proteome version in Ensembl Metazoa 52. Right bar plot is the proteome available in Ensembl Metazoa 53 and onward

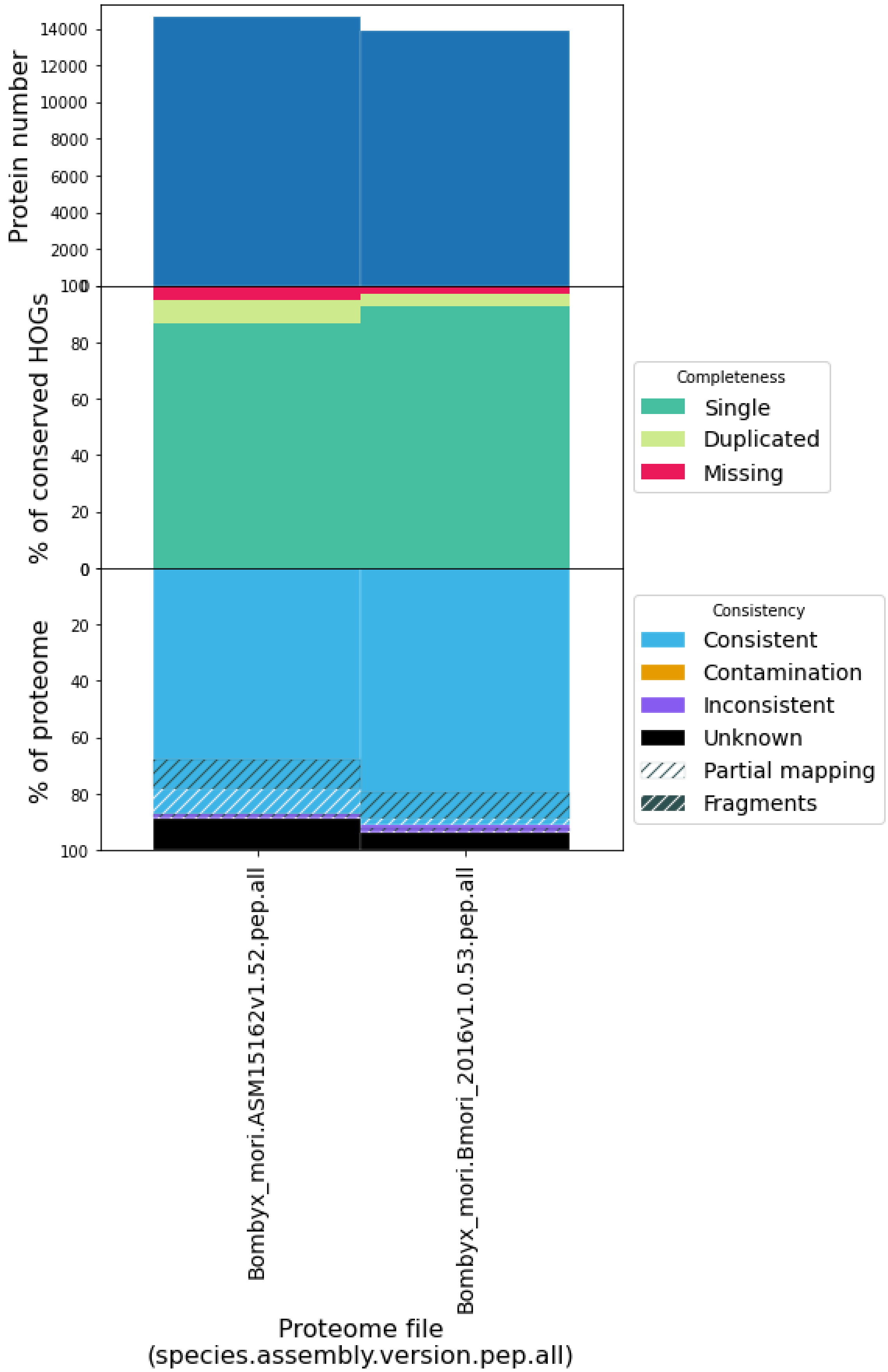

Supplementary Figures 39. OMArk comparison of different versions in *Bombyx mori* assemblies. Left bar plot corresponds to the proteome version in Ensembl Metazoa 52. Right bar plot is the proteome available in Ensembl Metazoa 53 and onward

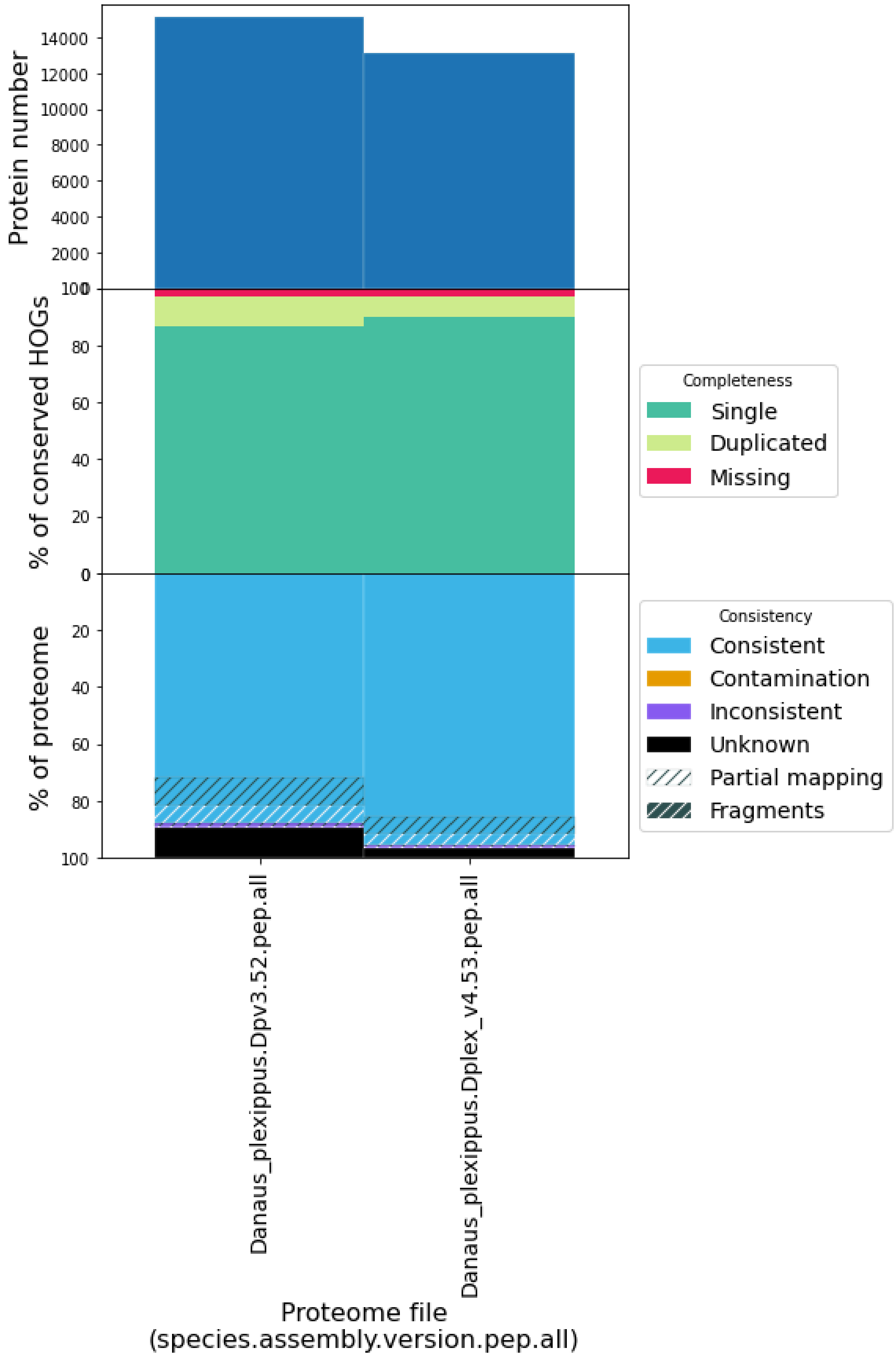

Supplementary Figures 40. OMArk comparison of different versions in *Danaus plexippus* assemblies. Left bar plot corresponds to the proteome version in Ensembl Metazoa 52. Right bar plot is the proteome available in Ensembl Metazoa 53 and onward

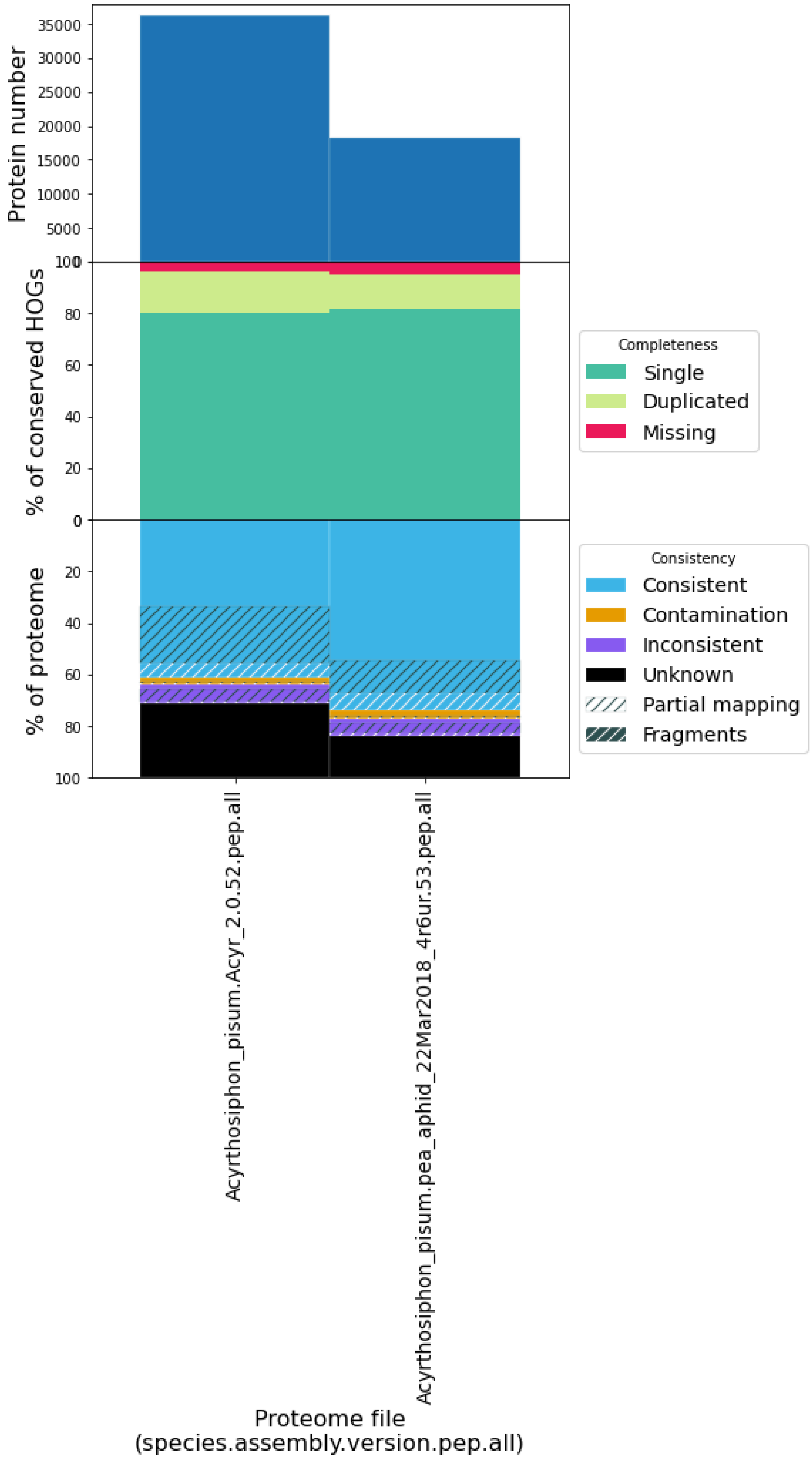

Supplementary Figures 41. OMArk comparison of different versions in *Acyrthosiphon pisum* assemblies. Left bar plot corresponds to the proteome version in Ensembl Metazoa 52. Right bar plot is the proteome available in Ensembl Metazoa 53 and onward

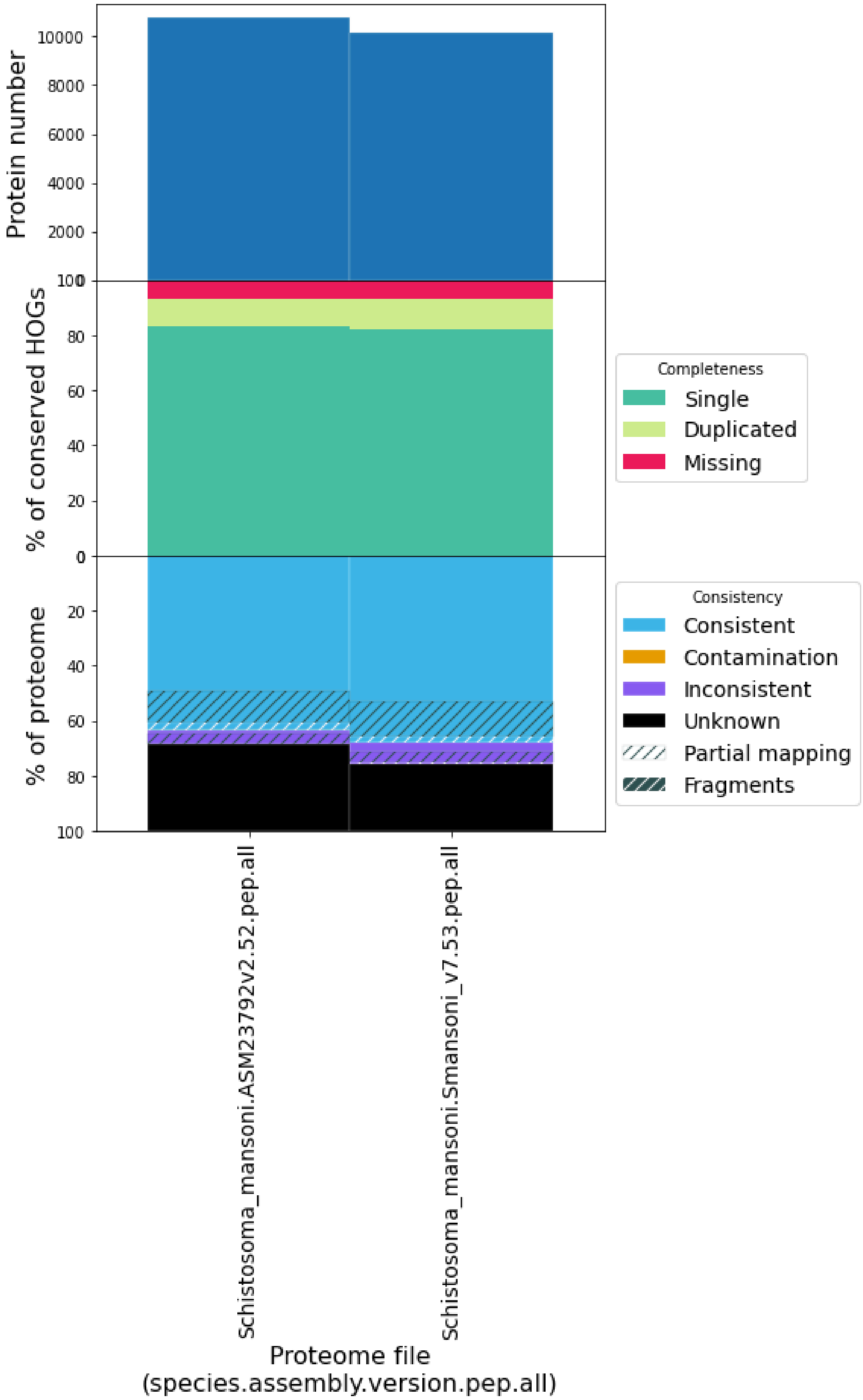

Supplementary Figures 42. OMArk comparison of different versions in *Schistosoma mansoni* assemblies. Left bar plot corresponds to the proteome version in Ensembl Metazoa 52. Right bar plot is the proteome available in Ensembl Metazoa 53 and onward

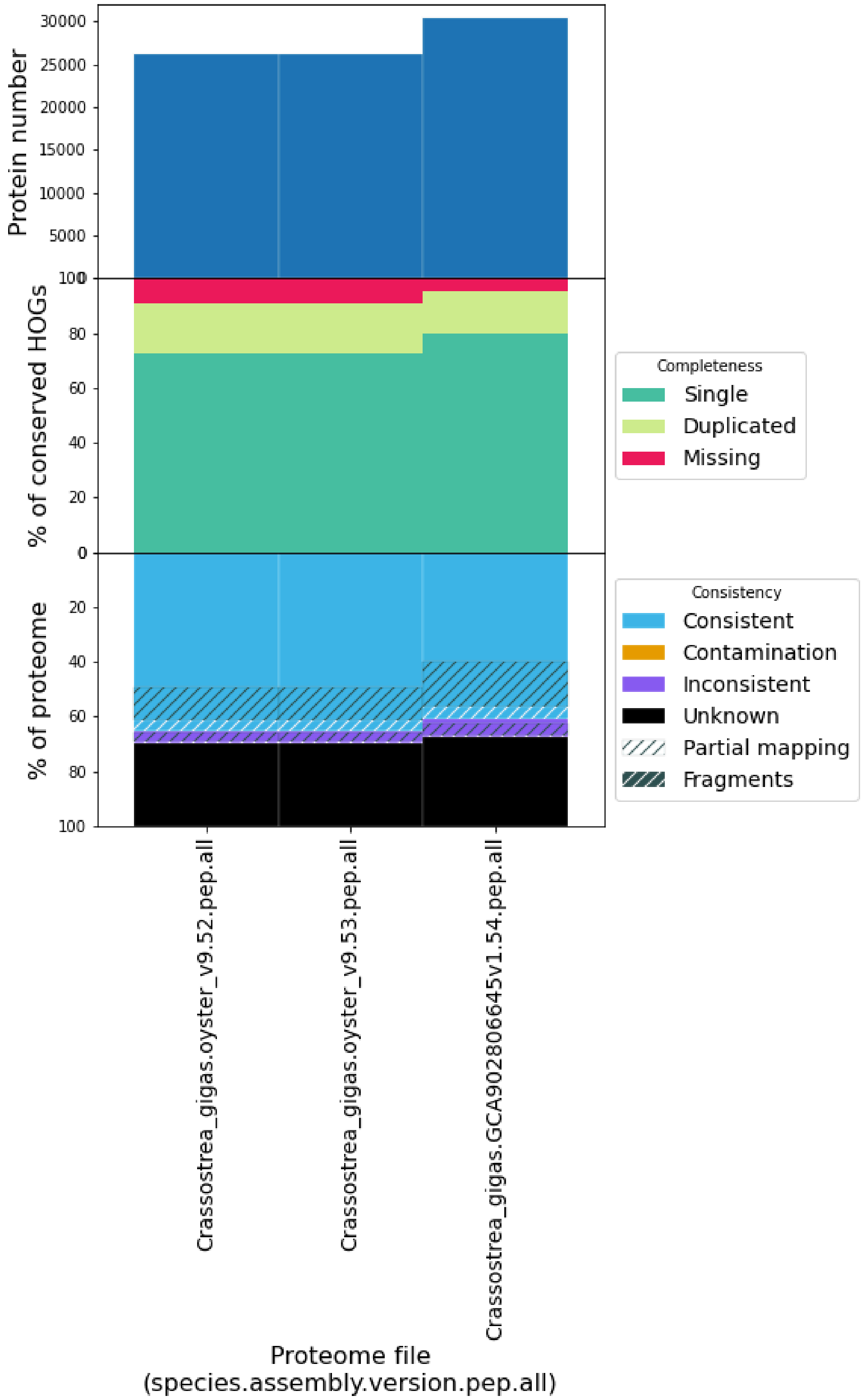

Supplementary Figures 43. OMArk comparison of different versions in Crassostrae gigas assemblies. Left bar plot corresponds to the proteome version in Ensembl Metazoa 52. Central bar plot is the proteome available in Ensembl Metazoa 53. Right bar plots the newest assembly available in Ensembl Metazoa 54.

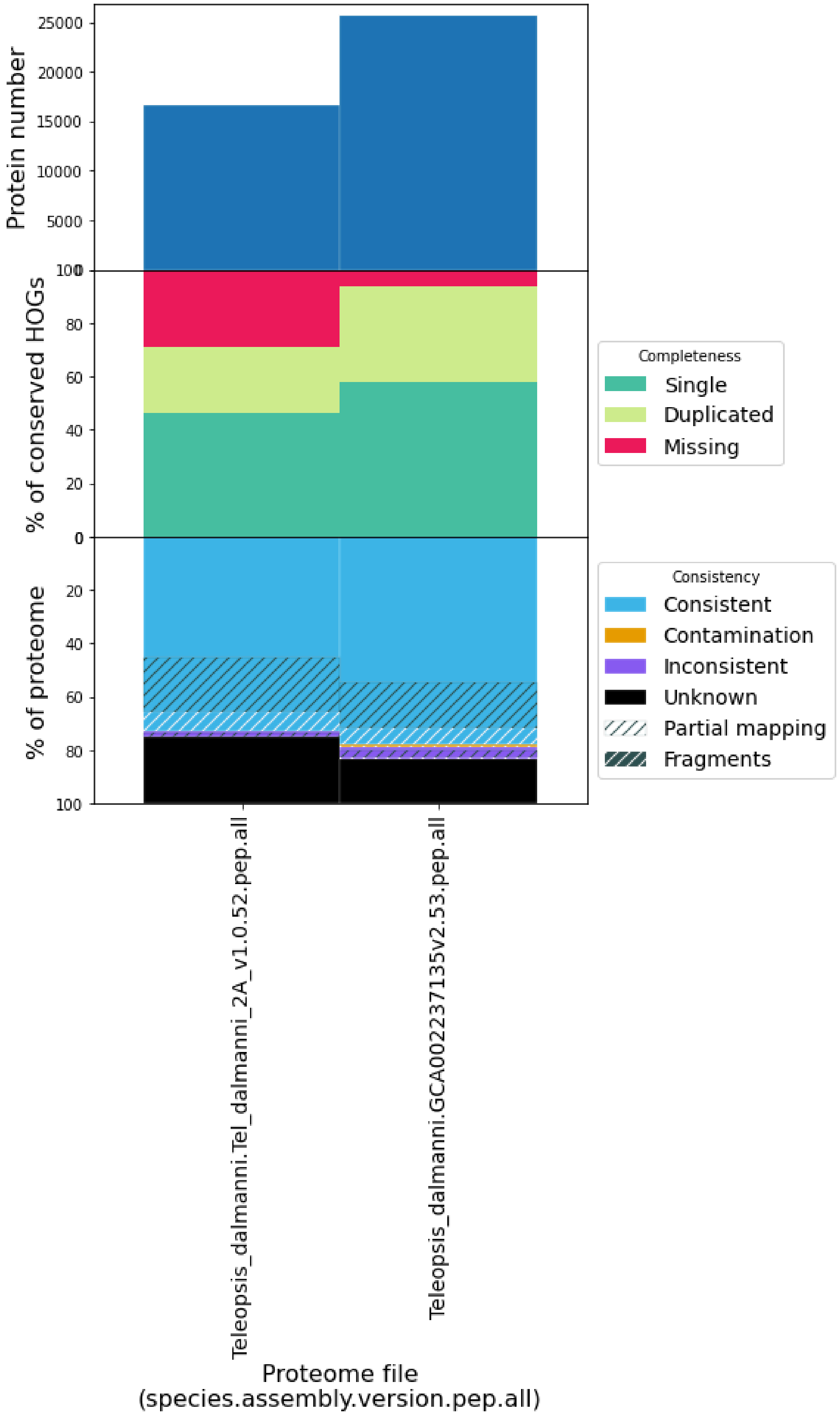

Supplementary Figures 44. OMArk comparison of different versions in *Teleopsis dalmanni* assemblies. Left bar plot corresponds to the proteome version in Ensembl Metazoa 52. Right bar plot is the proteome available in Ensembl Metazoa 53 and onward.

Annotation comparisons

The species with updated annotations in our dataset were all nematodes. The change in the gene set between annotations was minor according to OMArk results (few metrics with a change higher than 1%) and in terms of coding-gene number, likely reflective of iterative changes (Supplementary Figures 45-51). Absolute change in gene number was higher than 10 for 4 of the 7 proteomes, namely: *Caenorhabditis brenneri* (+30), *Brugia malayi* (-132), *Caenorhabditis briggsae* (-175), and *Caenorhabditis elegans* (-195).

Surprisingly, the change in gene content of the three latter species leads to slightly lower completeness as detected by OMArk (respectively, -0.01%, -0.04% and -0.15%), meaning some of the removed genes correspond to ones that are presumably conserved within the lineage. However, the observed changes in the same species are more prominent in the Duplicated category (respectively, -0.03%, -0.05% and -0.19%), hinting that part of this change is due to removal of spuriously duplicated genes. In the opposite direction, the added genes in *Caenorhabditis brenneri* led to a slight increase in completeness (0.02%) and a larger increase in duplicated genes (0.07%). The change in annotation in other species has a comparatively lower impact on OMArk statistics.

The change in gene set has a small impact on Taxonomical consistency, but a noticeable one for *C. elegans* (+0.13%) and *Brugia malayii* (+0.73%) meaning it leads to better quality metrics overall. This is also the case for taxonomically and structurally consistent proteins, where it increases by 0.31% for *C. elegans*, by 0.22% for *C. briggsea* and by 1.16% for *Brugia malayi*.

As expected, given no changes were made to the assembly, we noticed no significant differences in terms of contaminant for these species, with *Caenorhabditis japonica* being contaminated by a *Paenibacillus* species and *Caenorhabditis remanei* being contaminated with *Acinetobacter* species in both annotations according to OMArk.

To conclude, we show that while iterative changes in the annotation do not lead to large changes in OMArk statistics, it still provides information about the nature of these changes when used in a one-to-one comparison.

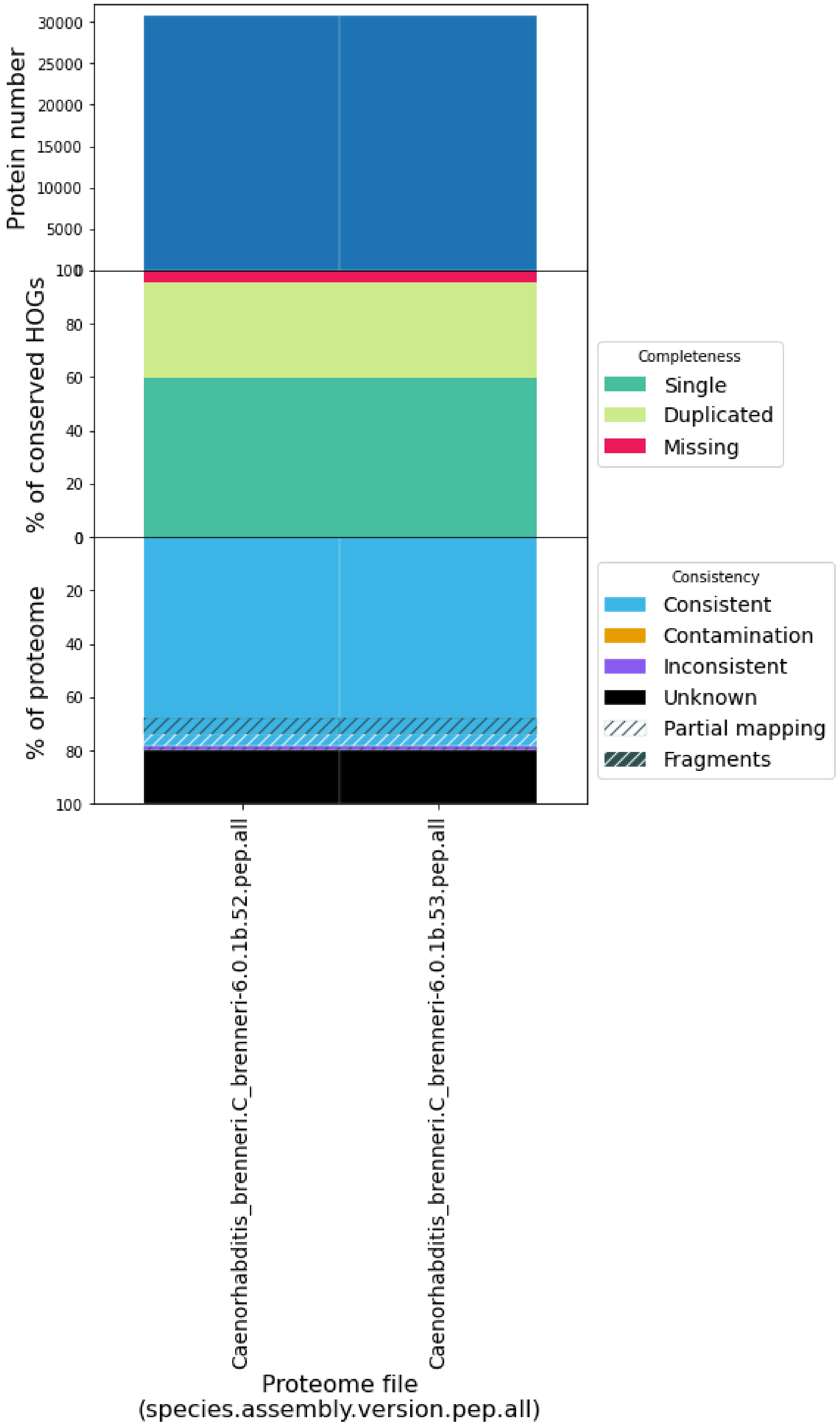

Supplementary Figures 45. OMArk comparison of different versions in *Caenorhabditis brenneri* annotation. Left bar plot corresponds to the proteome version in Ensembl Metazoa 52. Right bar plot is the proteome available in Ensembl Metazoa 53 and onward.

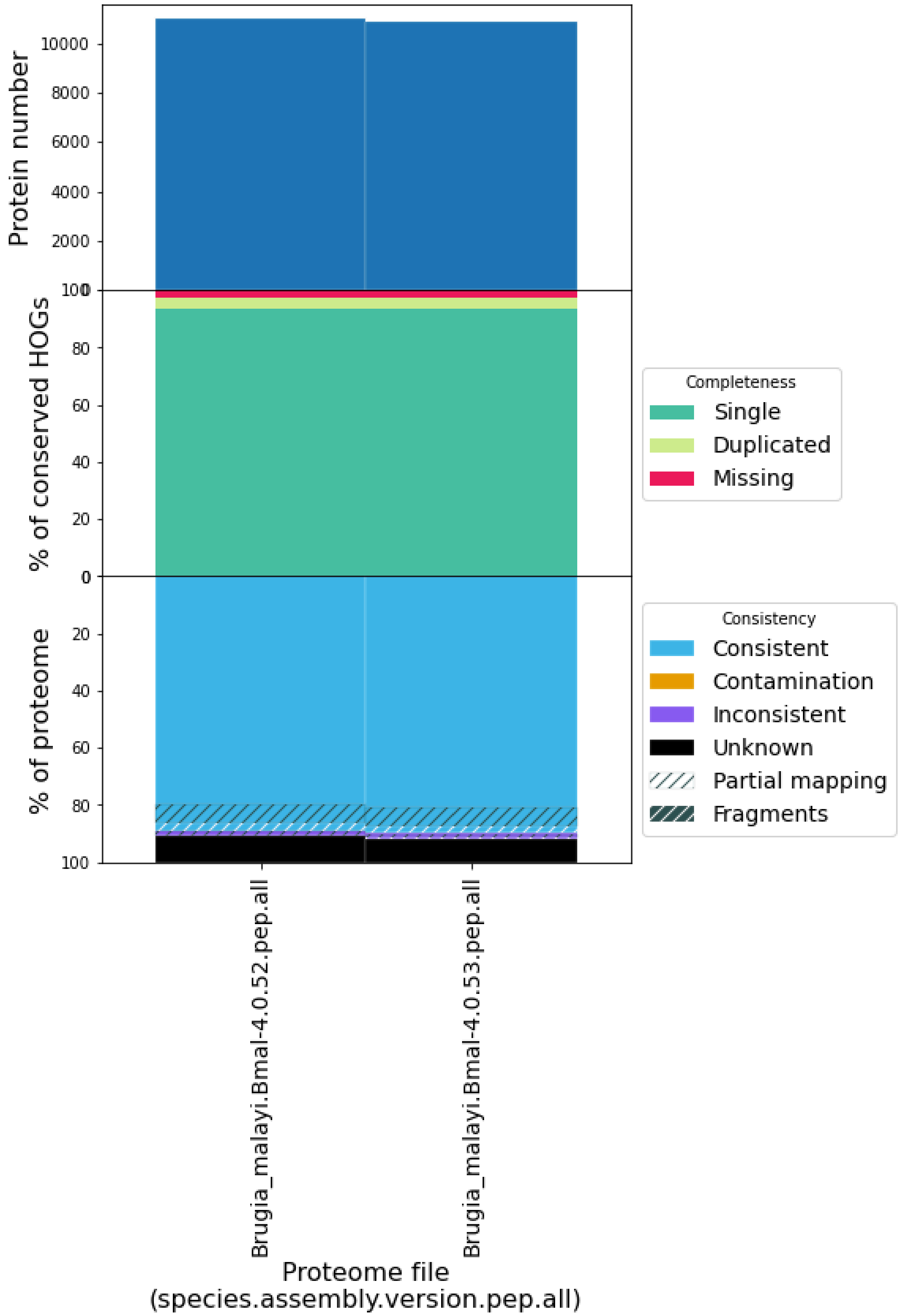

Supplementary Figures 46. OMArk comparison of different versions in *Brugia malayi* annotation. Left bar plot corresponds to the proteome version in Ensembl Metazoa 52. Right bar plot is the proteome available in Ensembl Metazoa 53 and onward.

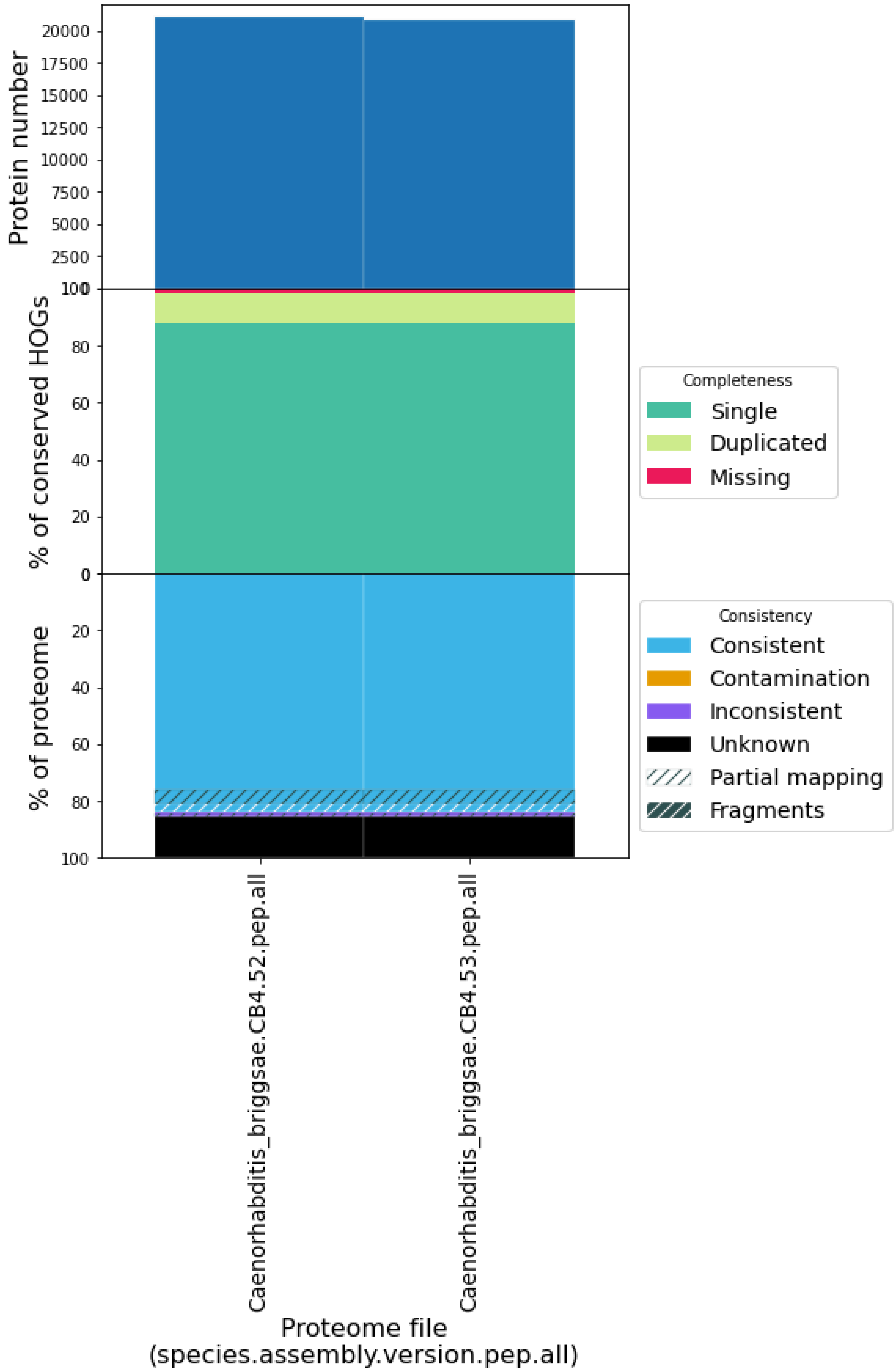

Supplementary Figures 47. OMArk comparison of different versions in *Caenorhabditis briggsae* annotation. Left bar plot corresponds to the proteome version in Ensembl Metazoa 52. Right bar plot is the proteome available in Ensembl Metazoa 53 and onward.

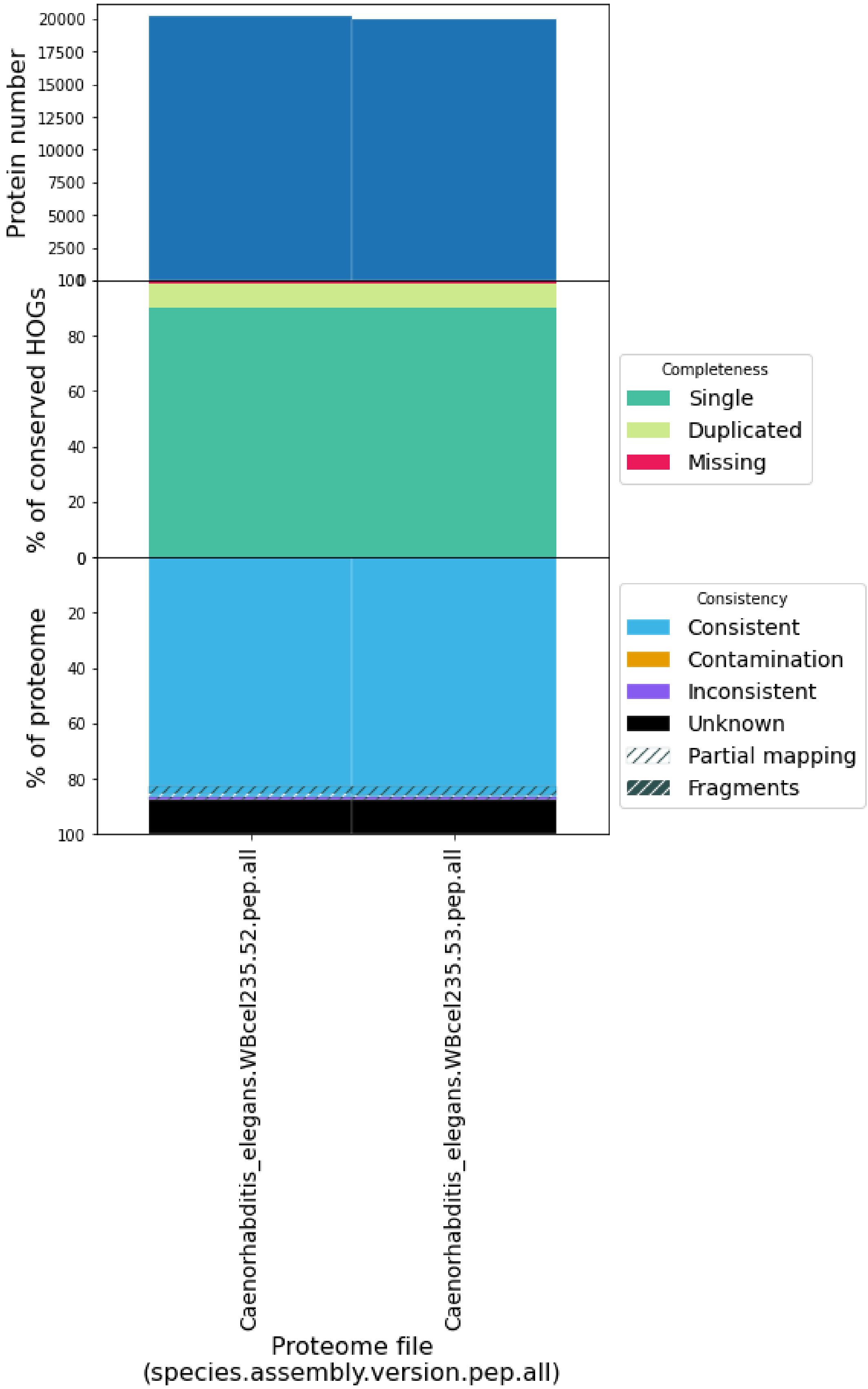

Supplementary Figures 48. OMArk comparison of different versions in *Caenorhabditis elegans* annotation. Left bar plot corresponds to the proteome version in Ensembl Metazoa 52. Right bar plot is the proteome available in Ensembl Metazoa 53 and onward.

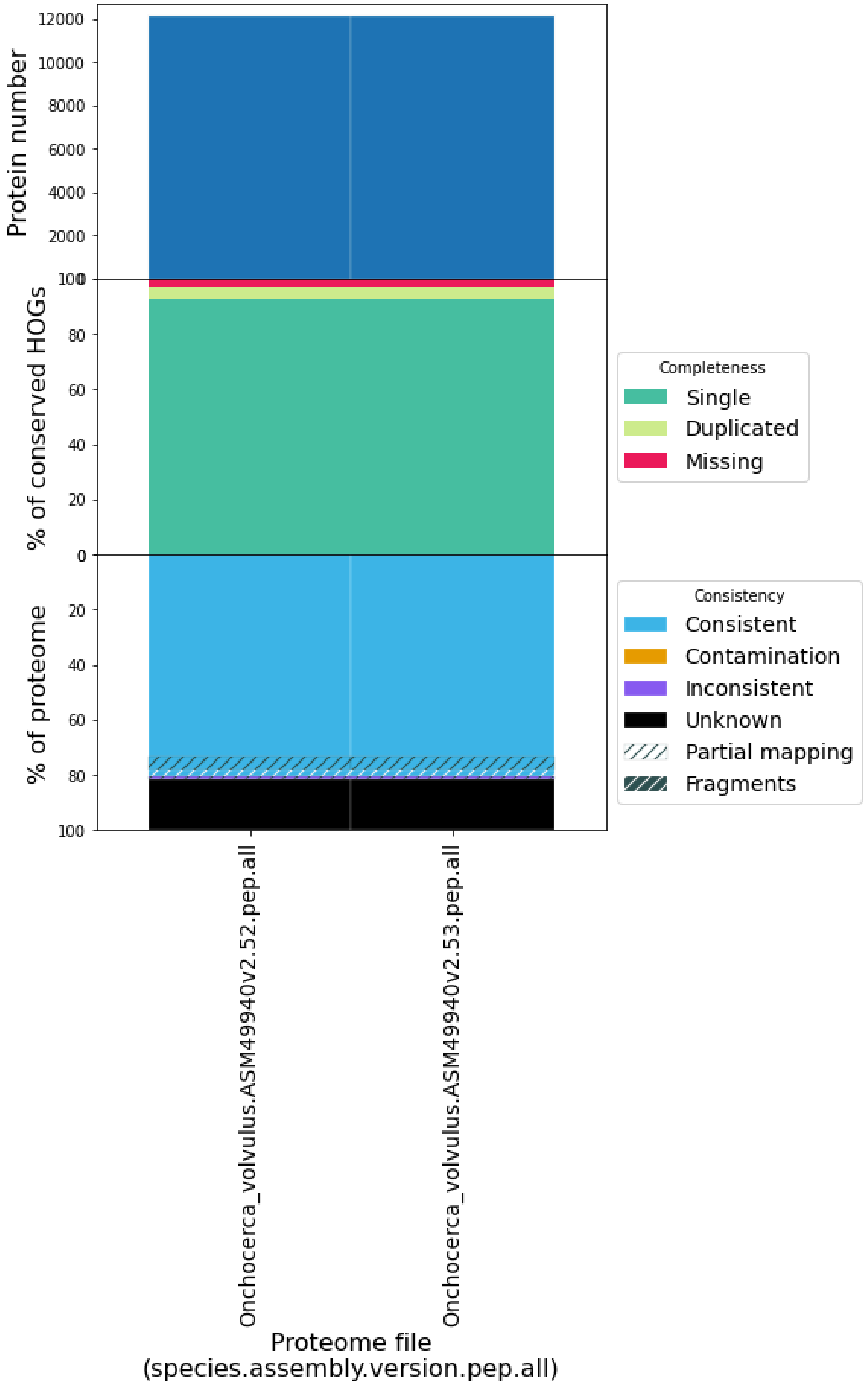

Supplementary Figures 49. OMArk comparison of different versions in *Onchocerca volvulus* annotation. Left bar plot corresponds to the proteome version in Ensembl Metazoa 52. Right bar plot is the proteome available in Ensembl Metazoa 53 and onward.

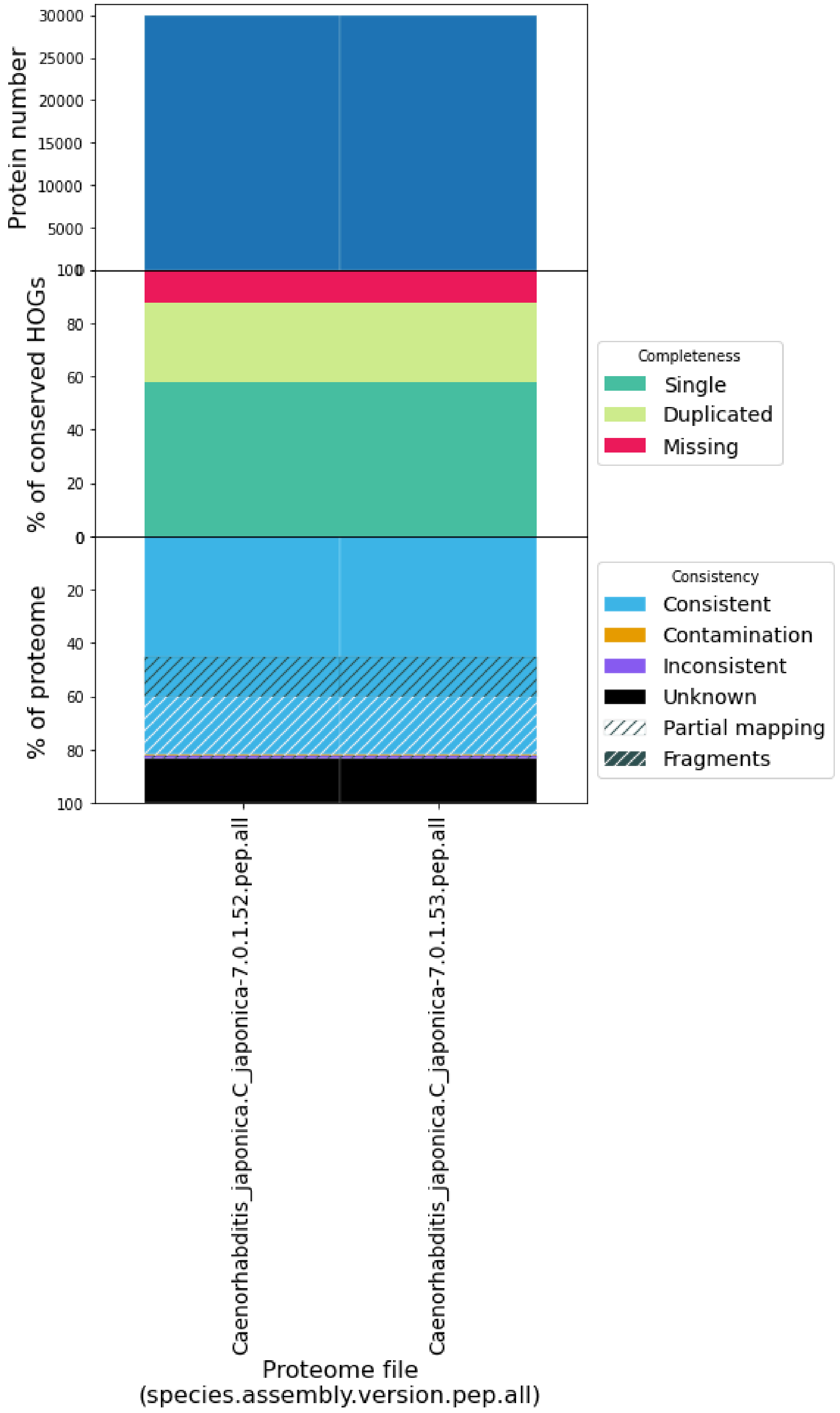

Supplementary Figures 50. OMArk comparison of different versions in *Caenorhabditis japonica* annotation. Left bar plot corresponds to the proteome version in Ensembl Metazoa 52. Right bar plot is the proteome available in Ensembl Metazoa 53 and onward.

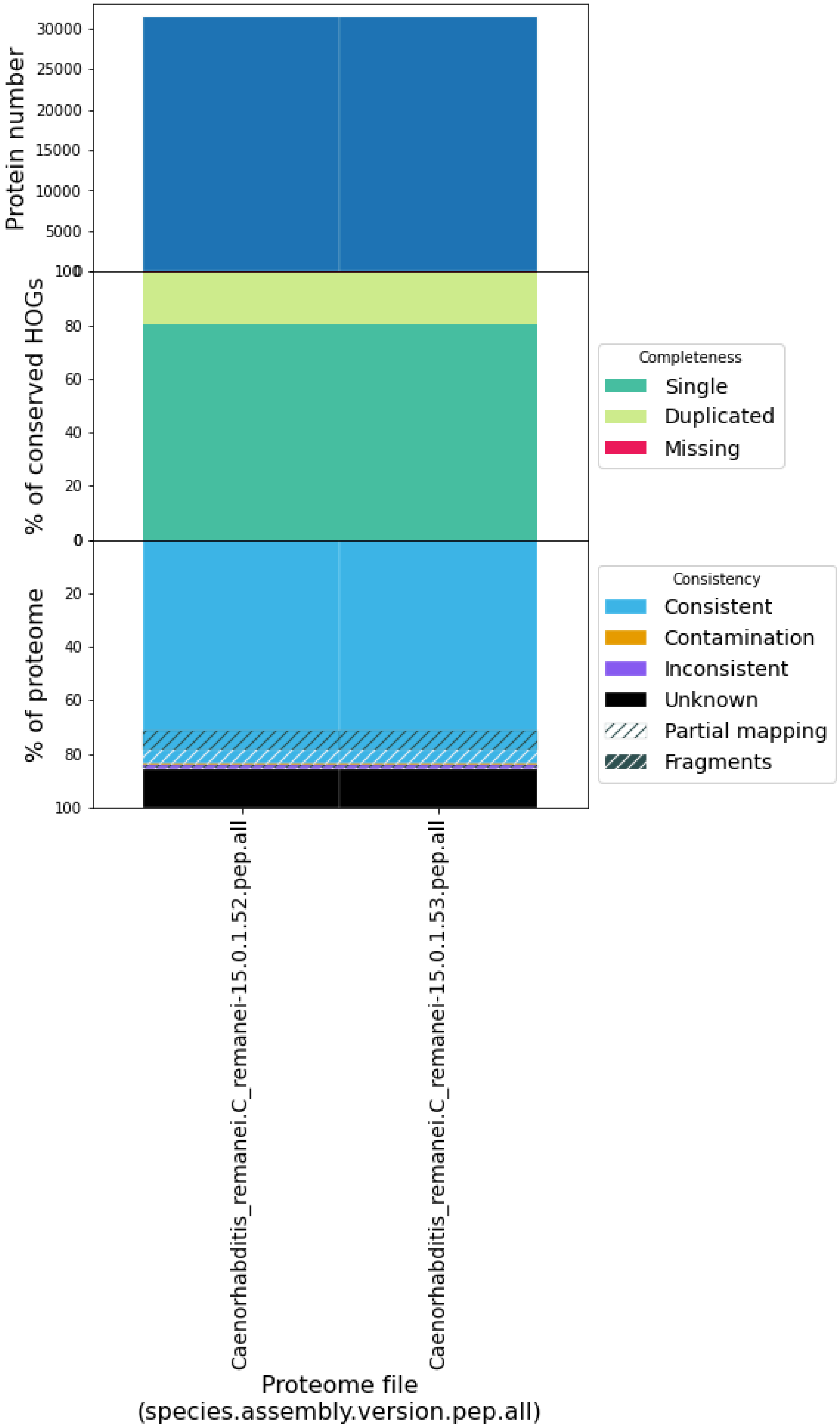

Supplementary Figures 51. OMArk comparison of different versions in *Caenorhabditis remanei* annotation. Left bar plot corresponds to the proteome version in Ensembl Metazoa 52. Right bar plot is the proteome available in Ensembl Metazoa 53 and onward.

Investigation of human missing genes

The aim of the following analysis is to investigate the genes in *Homo sapiens* deemed as missing by OMArk. We sought to validate if they were indeed true missing genes (cannot be found in the assembly), or simply missing from the annotation.

In the latest run of OMArk, there are 354 genes found as missing in human (i.e. ancestral Hominidae HOGs which have no human gene as a member of that HOG). We investigated these genes by taking a closely related gene to the human one, i.e. one that is in the same ancestral HOG which was deemed missing, but in another Hominidae species such as Gorilla. We randomly chose a gene in the same HOG at that taxonomic level. The number of times each species was used as the closely related gene for the 354 missing genes is shown in Supplementary Figure X.

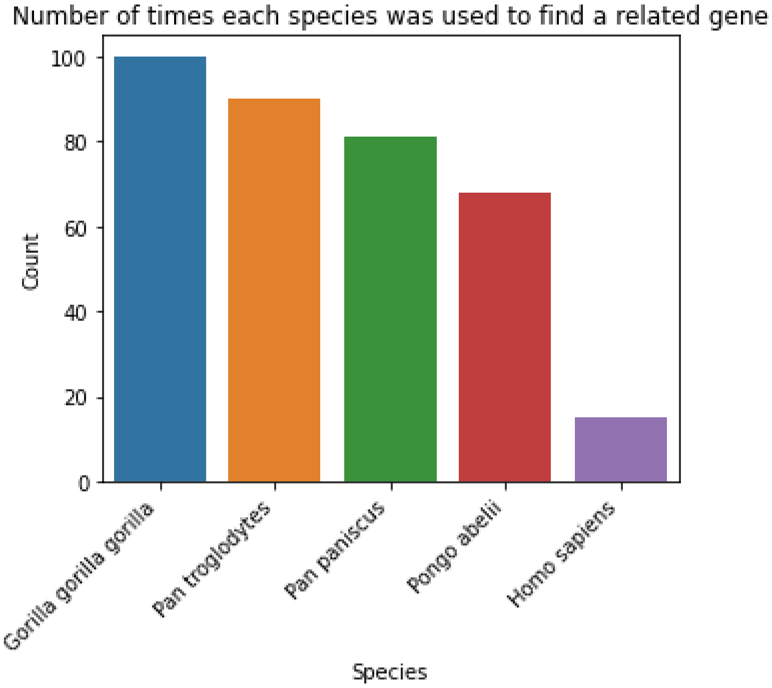

Supplementary Figure 52. Bar chart showing the species from which the closely related gene to the human "missing" gene was taken. Genes were chosen randomly from the HOG at the *Hominidae* taxonomic level.

Note that there are some human genes in this list. That is due to discrepancies between OMAmer and the main OMA database-- OMAmer maps the human query sequence to a different HOG than the main OMA algorithm places the sequence in. This is why occasionally there are human genes in the Missing HOGs.

We then used the cDNA of those genes and made a BLAST search against the latest Hg38 human assembly. The assembly used can be found at 'https://ftp.ensembl.org/pub/release-109/fasta/homo_sapiens/dna/Homo_sapiens.GRCh38.dna_rm.primary_assembly.fa.gz'. We created a blast database using this downloaded assembly. We executed the blastn with the fasta file of closely related genes to those deemed as missing in human against the formatted human assembly.

In order to obtain the latest annotated features from the human genome, we downloaded the GFF file from the Jbrowse2 desktop Version 2.7.0. It is the: feature track

"ncbi_refseq_109_hg38_latest", downloaded from https://s3.amazonaws.com/jbrowse.org/genomes/GRCh38/ncbi_refseq/GRCh38_latest_genomic.sort.gff.gz. This file contains a number of features besides genes, including pseudogenes, mRNA, repeat regions, etc. Using Bedtools 2.31.0, we found the intersection of annotated features from the human genome and our blast hits of missing human genes.

We found that 95% of the genes that were deemed as missing by OMArk actually had an overlap with a feature annotated as a gene or pseudogene in the human GFF file. Only 1.5% of the missing HOGs (5 genes) had no blast hit at all, and only 1.12% (4 genes) had a blast hit, but it did not overlap with any feature on the human genome. Note that there was no threshold for significant blast hits applied beyond the default. Therefore, if missing genes belonged to multigene families, it is possible that they may hit one of the paralogs in the genome. However, this means that the missing genes with no blast hits are likely bone fide missing genes.

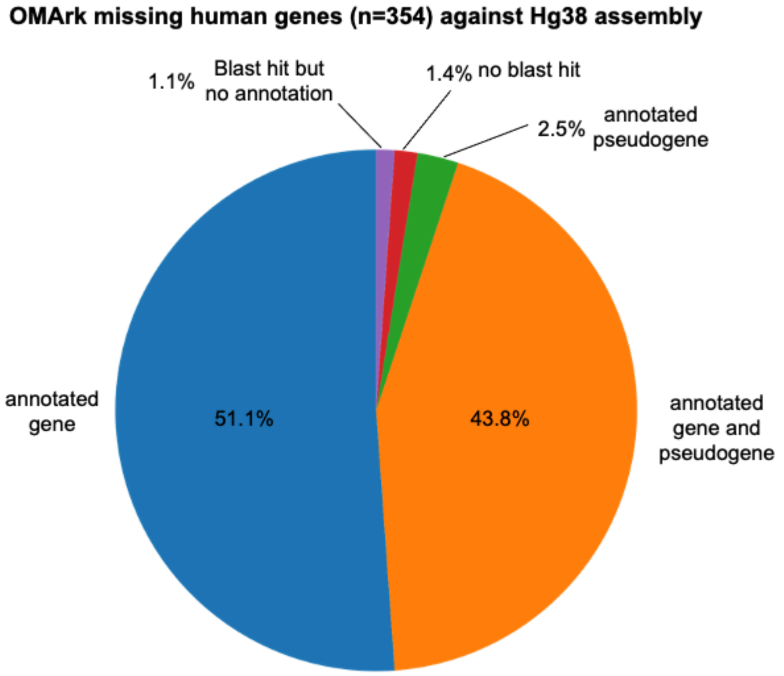

Supplementary Figure 53. Pie chart of the 354 missing genes in human as determined by OMark and the results of blasting agains the human hg38 assembly. 51% had an overlap with an annotated gene feature, 43.8% with an annotated gene and a pseudogene feature, and 2.5% had an overlap with just an annotated pseudogene feature. Only 1.1% had a blast hit but no annotation, and 1.4% had no blast hits at all.

The reasons why the genes are deemed by OMArk as missing can be due to several reasons. First, OMAmer may fail to initially map the human query protein to any HOG in OMArk. Second, there may be a discrepancy against the HOG Omamer maps the query protein to and the HOG which OMA placed the protein in in the main OMA database. Finally, the gene simply may not be found in the human annotation due to it being a pseudogene or a non-coding RNA (see examples in Supplementary Figures 54, 55, and 56).

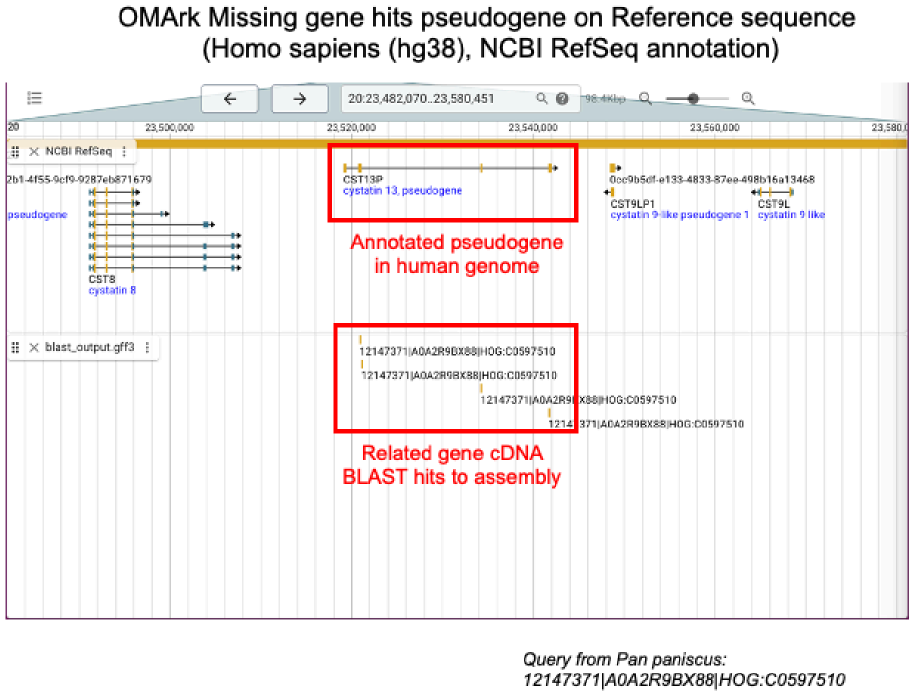

Supplementary Figure 54. One of the genes deemed missing by OMArk (sequence from Pan paniscus gene A0A2R9BX88 in HOG:C0597510). Blastn against the Hg38 assembly shows it overlaps with CST13P pseudogene.

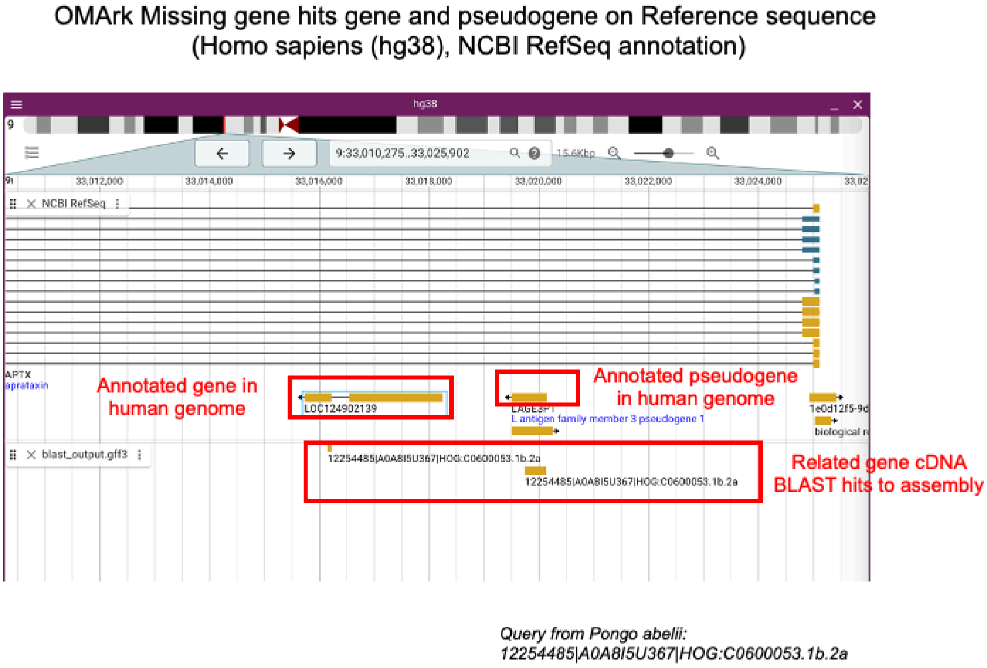

Supplementary Figure 55. One of the genes deemed missing by OMArk (sequence from *Pongo abelii* gene A0A8I5U367 in HOG:C0600053.1b.2a). Blastn against the Hg38 assembly shows it overlaps with LAGE3P1 pseudogene and the LOC124902139 gene.

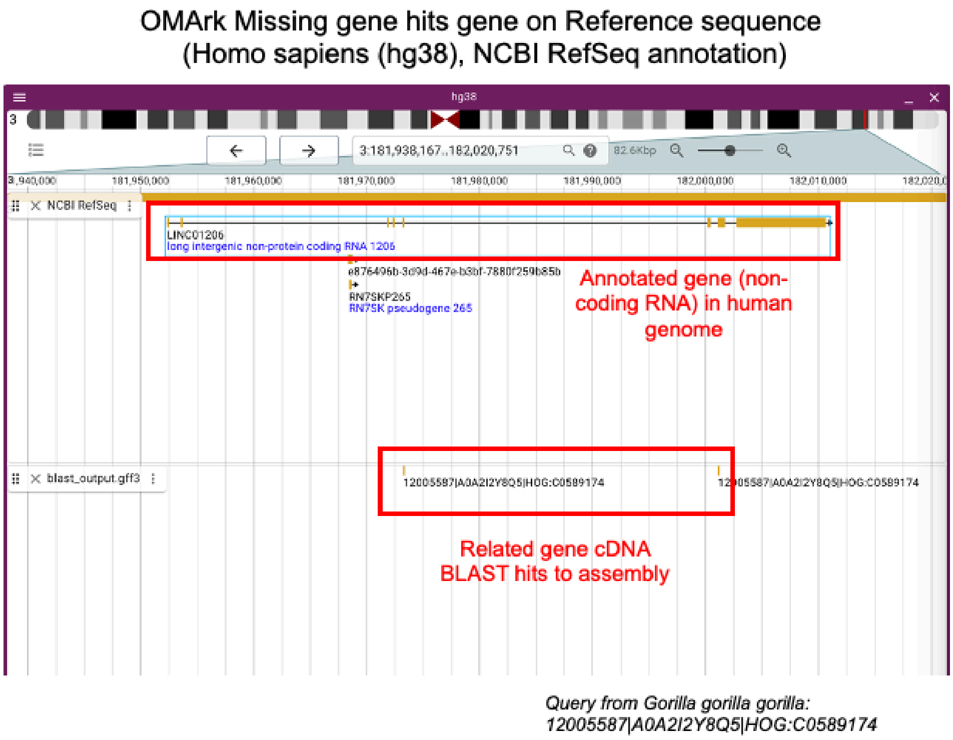

Supplementary Figure 56. One of the genes deemed missing by OMArk (sequence from *Gorilla gorilla* gene A0A2I2Y8Q5 in HOG:C0589174). Blastn against the Hg38 assembly shows it overlaps with LINC01206 long intergenic non-protein coding RNA.

The results of this analysis demonstrate that genes determined as missing may in fact be mostly noise due to discrepancies in annotation. We emphasize the importance of verifying any lost genes with downstream analyses. However, OMArk shines when used to compare with other species, so any proteomes with outlier numbers of missing genes could be considered dubious.

These analyses are detailed in the supplementary material and the notebook is provided (“human_missing_genes.ipynb”).

Supplementary references

1. Manni, M., Berkeley, M. R., Seppey, M., Simão, F. A. & Zdobnov, E. M. BUSCO Update: Novel and Streamlined Workflows along with Broader and Deeper Phylogenetic Coverage for Scoring of Eukaryotic, Prokaryotic, and Viral Genomes. *Mol. Biol. Evol.* **38**, 4647–4654 (2021).
2. Maeda, Y. *et al.* Chromosome scale assembly of allopolyploid genome of the diatom Fistulifera solaris. *bioRxiv* 2021.11.10.468027 (2021) doi:10.1101/2021.11.10.468027.
3. Hunt, H. V. *et al.* Reticulate evolution in Panicum (Poaceae): the origin of tetraploid broomcorn millet, P. miliaceum. *J. Exp. Bot.* **65**, 3165–3175 (2014).
4. Slabodnick, M. M. *et al.* The Macronuclear Genome of Stentor coeruleus Reveals Tiny Introns in a Giant Cell. *Curr. Biol.* **27**, 569–575 (2017).
5. Kim, Y.-M. *et al.* Genome analysis of Hibiscus syriacus provides insights of polyploidization and indeterminate flowering in

woody plants. *DNA Res.* **24**, 71–80 (2017).

6. UniProt Consortium. UniProt: the universal protein knowledgebase in 2021. *Nucleic Acids Res.* **49**, D480–D489 (2021).
7. Dai, Y. *et al.* Genomic Analyses Provide Insights Into the Evolutionary History and Genetic Diversity of Auricularia Species. *Front. Microbiol.* **10**, 2255 (2019).
8. Lhee, D. *et al.* Evolutionary dynamics of the chromatophore genome in three photosynthetic Paulinella species. *Sci. Rep.* **9**, 2560 (2019).
9. Cunningham, F. *et al.* Ensembl 2022. *Nucleic Acids Res.* **50**, D988–D995 (2022).
10. O'Leary, N. A. *et al.* Reference sequence (RefSeq) database at NCBI: current status, taxonomic expansion, and functional annotation. *Nucleic Acids Res.* **44**, D733–45 (2016).
11. Feng, S. *et al.* Dense sampling of bird diversity increases power of comparative genomics. *Nature* **587**, 252–257 (2020).
12. Rhie, A. *et al.* Towards complete and error-free genome assemblies of all vertebrate species. *Nature* **592**, 737–746 (2021).
13. Xu, P. *et al.* The allotetraploid origin and asymmetrical genome evolution of the common carp Cyprinus carpio. *Nat. Commun.* **10**, 4625 (2019).
14. Yang, J. *et al.* The genome sequence of allopolyploid Brassica juncea and analysis of differential homoeolog gene expression influencing selection. *Nat. Genet.* **48**, 1225–1232 (2016).
15. Yates, A. D. *et al.* Ensembl Genomes 2022: an expanding genome resource for non-vertebrates. *Nucleic Acids Res.* **50**, D996–D1003 (2022).
