# Supplementary material for: Quality assessment of gene repertoire annotations with OMArk
Source: Nat Biotechnol. 2024 Feb 21;43(1):124–33. doi: 10.1038/s41587-024-02147-w (PMC11738984; doi:10.1038/s41587-024-02147-w)
Supplement: Supplementary file 2 — Reporting Summary [file 41587_2024_2147_MOESM2_ESM.pdf]

nature portfolio

Corresponding author(s): Yannis Nevers

Last updated by author(s): 11 Jan 2024

Reporting Summary

Nature Portfolio wishes to improve the reproducibility of the work that we publish. This form provides structure for consistency and transparency in reporting. For further information on Nature Portfolio policies, see our Editorial Policies and the Editorial Policy Checklist.

Statistics

For all statistical analyses, confirm that the following items are present in the figure legend, table legend, main text, or Methods section.

n/a	Confirmed	
☐	☒	The exact sample size (*n*) for each experimental group/condition, given as a discrete number and unit of measurement
☒	☐	A statement on whether measurements were taken from distinct samples or whether the same sample was measured repeatedly
☐	☒	The statistical test(s) used AND whether they are one- or two-sided<br>*Only common tests should be described solely by name; describe more complex techniques in the Methods section.*
☒	☐	A description of all covariates tested
☒	☐	A description of any assumptions or corrections, such as tests of normality and adjustment for multiple comparisons
☐	☒	A full description of the statistical parameters including central tendency (e.g. means) or other basic estimates (e.g. regression coefficient) AND variation (e.g. standard deviation) or associated estimates of uncertainty (e.g. confidence intervals)
☐	☒	For null hypothesis testing, the test statistic (e.g. *F*, *t*, *r*) with confidence intervals, effect sizes, degrees of freedom and *P* value noted<br>*Give P values as exact values whenever suitable.*
☒	☐	For Bayesian analysis, information on the choice of priors and Markov chain Monte Carlo settings
☒	☐	For hierarchical and complex designs, identification of the appropriate level for tests and full reporting of outcomes
☒	☐	Estimates of effect sizes (e.g. Cohen's *d*, Pearson's *r*), indicating how they were calculated

Our web collection on statistics for biologists contains articles on many of the points above.

Software and code

Policy information about availability of computer code

Data collection	NCBI data were downloaded using the NCBI datasets library version 13.28.1 and Python 3.9 and other proteomes were downloaded via a ftp server through wget verison 1.19.5.
Data analysis	The software for data analysis, OMArk (v0.3.0), is available on GitHub (https://github.com/DesimozLab/OMArk) and as a python package on PyPI. OMArk was run using Python 3.9.5 and installed with PyPI version 22. We used OMAmer v2.0.0 for our protein placement. We used BUSCO v5.2.2 for all BUSCO analyses. The versioned code is also available on Zenodo (doi:10.5281/zenodo.10474466). Additional analyses were perfomed with the same version of Python and Jupyter Notebooks (Jupyter v1.0.0). Figures were generated with Matplotlib (v3.4.2) and Seaborn (v0.11.2). All Jupyter Notebooks used for the analysis are made available through Zenodo (doi:10.5281/zenodo.10034236).

For manuscripts utilizing custom algorithms or software that are central to the research but not yet described in published literature, software must be made available to editors and reviewers. We strongly encourage code deposition in a community repository (e.g. GitHub). See the Nature Portfolio guidelines for submitting code & software for further information.

Data

Policy information about availability of data

All manuscripts must include a data availability statement. This statement should provide the following information, where applicable:

- Accession codes, unique identifiers, or web links for publicly available datasets
- A description of any restrictions on data availability
- For clinical datasets or third party data, please ensure that the statement adheres to our policy

UniProt Reference proteomes were downloaded from UniProtKB on February 1st 2022 (version 04/2021) through their ftp server. Ensembl Metazoa proteomes were downloaded from their ftp website from version 52, 53 and 54. NCBI proteomes were downloaded in August 2023 through the NCBI datasets python library, and proteomes from Ensembl 110 and Ensembl databases 57 were downloaded through their respective ftp websites. All datasets used and generated during the study and Supplementary Table files are made available through Zenodo (doi:10.5281/zenodo.10034236). Precomputed results for UniProt, GenBank and Ensembl are made available through the OMArk webserver (https://omark.omabrowser.org).

Human research participants

Policy information about studies involving human research participants and Sex and Gender in Research.

Reporting on sex and gender	N/A
Population characteristics	N/A
Recruitment	N/A
Ethics oversight	N/A

Note that full information on the approval of the study protocol must also be provided in the manuscript.

Field-specific reporting

Please select the one below that is the best fit for your research. If you are not sure, read the appropriate sections before making your selection.

☒ Life sciences ☐ Behavioural & social sciences ☐ Ecological, evolutionary & environmental sciences

For a reference copy of the document with all sections, see nature.com/documents/nr-reporting-summary-flat.pdf

Life sciences study design

All studies must disclose on these points even when the disclosure is negative.

Sample size	Sample size for the simulation analyses was 9 for the 'Model' dataset and 16 for the 'Representative' dataset. The Model dataset is a manually curated dataset based on available model species in OMA, and the Representative dataset was manually curated to represent diverse species not available in OMA. The sample size for the UniProt Reference Proteome analysis was 1,805, using all available Reference Proteomes in the UniProt database. The sample size for the Ensembl annotation and assembly comparison was 18, based on the list of species with either an annotation or assembly change for the two previous releases of Ensembl metazoa (53 and 54). The sample size for the Ensembl and NCBI comparison was 1,200, i.e. proteomes for which we could find an annotation version in both the NCBI database and in the Ensembl database.
Data exclusions	No data was excluded from the analysis.
Replication	Data used in all their analyses were downloaded from public databases and not generated for this study, thus preventing possibility of replicates.
Randomization	Randomization is not applicable as no experiments were carried out for this study, which only uses publicly available data.
Blinding	Blinding is not applicable as no experiments were carried out for this study, which only uses publicly available data.

Reporting for specific materials, systems and methods

We require information from authors about some types of materials, experimental systems and methods used in many studies. Here, indicate whether each material, system or method listed is relevant to your study. If you are not sure if a list item applies to your research, read the appropriate section before selecting a response.

Materials & experimental systems

n/a	Involved in the study
☒	☐ Antibodies
☒	☐ Eukaryotic cell lines
☒	☐ Palaeontology and archaeology
☒	☐ Animals and other organisms
☒	☐ Clinical data
☒	☐ Dual use research of concern

Methods

n/a	Involved in the study
☒	☐ ChIP-seq
☒	☐ Flow cytometry
☒	☐ MRI-based neuroimaging
